# Rare Diseases, Spotlighting Amyotrophic Lateral Sclerosis, Huntington’s Disease, and Myasthenia Gravis: Insights from Landscape Analysis of Current Research

**DOI:** 10.1021/acs.biochem.4c00722

**Published:** 2025-04-01

**Authors:** Kavita A. Iyer, Rumiana Tenchov, Janet M. Sasso, Krittika Ralhan, Jyotsna Jotshi, Dmitrii Polshakov, Ankush Maind, Qiongqiong Angela Zhou

**Affiliations:** † CAS, A Division of the American Chemical Society, Columbus, Ohio 43210, United States; ‡ ACS International India Pvt. Ltd., Pune 411044, India

**Keywords:** rare disease, amyotrophic lateral sclerosis, Huntington’s disease, myasthenia gravis, gene, pathogenesis, neurodegeneration, autoimmunity

## Abstract

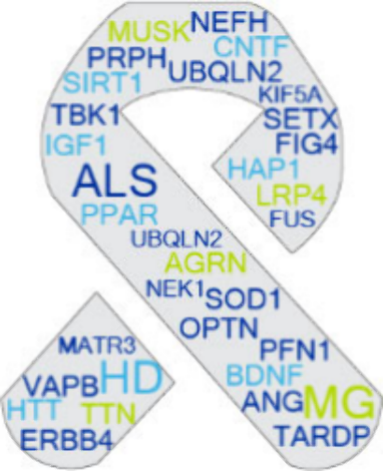

Rare diseases are a diverse group of disorders that, despite each individual condition’s rarity, collectively affect a significant portion of the global population. Currently approximately 10,000 rare diseases exist globally, with 80% of these diseases being identified as having genetic origins. In this Review, we examine data from the CAS Content Collection to summarize scientific progress in the area of rare diseases. We examine the publication landscape in the area in an effort to provide insights into current advances and developments. We then discuss the evolution of key concepts in the field, genetic associations, as well as the major technologies and development pipelines of rare disease treatments. We focus our attention on three specific rare diseases: (i) amyotrophic lateral sclerosis, a terminal neurodegenerative disease affecting the central nervous system resulting in progressive loss of motor neurons that control voluntary muscles; (ii) Huntington’s disease, another terminal neurodegenerative disease that causes progressive degeneration of nerve cells in the brain, with a wide impact on a person’s functional abilities; and (iii) myasthenia gravis, a chronic autoimmune synaptopathy leading to skeletal muscle weakness. While the pathogenesis of these rare diseases is being elucidated, there is neither a cure nor preventative treatment available, only symptomatic treatment. The objective of the paper is to provide a broad overview of the evolving landscape of current knowledge on rare diseases and specifically on the biology and genetics of the three spotlighted diseases, to outline challenges and evaluate growth opportunities, an aim to further efforts in solving the remaining challenges.

Rare diseases, also known as orphan diseases, are a diverse group of disorders that, despite each individual condition’s rarity, collectively affect a significant portion of the global population. The exact definition of a rare disease varies from country to country, but generally, a disease is considered rare when it affects a limited number of people within a specific region or population. The World Health Organization (WHO) defines a rare disease as one that strikes fewer than 65 per 100,000 people.^1^ According to the National Organization of Rare Disorders of the USA,^2^ a rare disorder is a disease or condition that affects fewer than 200,000 Americans (i.e., less than ∼60 per 100,000).^3,4^ In the European Union, a rare disease is one that affects no more than 1 person in 2000^5^ (i.e., <50 per 100,000). According to Global Genes, a global nonprofit advocacy organization for individuals fighting rare and genetic diseases,^6^ currently approximately 10,000 rare diseases exist globally, with new ones being continually discovered. About 80% of these diseases have been identified as of genetic origins,^7^ and they often manifest in diverse and unpredictable ways.

Despite their individual rarity, rare diseases collectively impact millions of individuals worldwide – approximately 4–6% of the worldwide population, equivalent to from 300 to over 400 million people.^1,8,9^ These conditions often present unique challenges due to their unfamiliarity, limited treatment options, and the difficulties associated with diagnosis. One of the primary challenges associated with rare diseases is their diagnosis. Due to their rarity and often complex clinical presentations, rare diseases are frequently misdiagnosed or undiagnosed altogether. This diagnostic struggle can be emotionally and financially difficult for patients and their families, leading to delays in appropriate treatment and care.

Moreover, the limited understanding of many rare diseases poses significant obstacles to the development of effective therapies. With only a handful of patients available for clinical trials, research into these conditions is often underfunded and progresses at a slower pace compared to more common diseases. Consequently, individuals living with rare diseases may have few, if any, treatment options available to them. As rare diseases each individually affect a small number of individuals, they have been ‘orphaned’ by the pharmaceutical industry, which has promoted the use of the term ‘orphan disease’ when referring to these conditions.^1^

Another critical issue facing the rare disease community is the lack of specialized healthcare providers and support services. Many rare diseases require multidisciplinary care from experts in various medical specialties, but access to such expertise can be limited, particularly in rural or underserved areas. Globally, less than 10% of patients with rare diseases receive disease-specific treatment.^1^

However, recent years have seen a growing awareness of rare diseases, leading to increased efforts to address the unmet needs of those affected. Since most rare diseases are genetic in their etiology, systematic research on them starts with efforts to identify genetic variants causative for each particular disease, with links between genetic mutations and diseases identified.^10,11^ Advances in genomic sequencing technologies and precision medicine hold promise for improved diagnosis and targeted treatments for many rare diseases. Additionally, initiatives such as orphan drug legislation^12−14^ and incentives for rare disease research^15−17^ have motivated pharmaceutical companies to invest in the development of therapies for these often-neglected conditions.

In this paper, we examine data from the CAS Content Collection.^18^ The CAS Content Collection is the largest human-curated collection of published scientific information, supporting comprehensive quantitative analysis of global research across parameters including time, geography, scientific discipline, application, disease, chemical composition, etc. Covering scientific literature published around the world in more than 50 languages, the CAS Content Collection encompasses data and discoveries published in more than 50,000 scientific journals and by over 100 patent offices. A major advantage provided by the CAS Content Collection is that, along with the standard reference information, it also provides human curated data on major substances and concepts explored in the scientific publications. The CAS REGISTRY,^19^ the authoritative source for information on more than 250 million unique organic and inorganic substances and 70 million protein and nucleic acid sequences, is part of the CAS Content Collection. The CAS Content Collection is broadly accessible through CAS solutions including CAS SciFinder^20^ and CAS STNext.^21^

Here we discuss the evolution of key concepts in the field as well as the major technologies and the development pipelines of rare disease treatments. We focus our attention on three specific rare diseases to perform a deeper dive into the research outlook in order to identify and understand obscure connections in these topics, namely: (i) amyotrophic lateral sclerosis (ALS), a terminal neurodegenerative disease affecting the central nervous system (CNS) resulting in progressive loss of motor neurons that control voluntary muscles; (ii) Huntington’s disease (HD), another terminal neurodegenerative disease that causes progressive degeneration of nerve cells in the brain, with a wide impact on a person’s functional abilities; and (iii) myasthenia gravis (MG), a chronic autoimmune synaptopathy leading to skeletal muscle weakness.

The objective of the paper is to provide a broad overview of the evolving landscape of current knowledge on rare diseases, to outline challenges, and evaluate growth opportunities, all with an aim to further efforts in solving the problems that still plague the field. The novelty and merit of the article stem from the extensive, wide-ranging coverage of the most up-to-date scientific information accumulated in the CAS Content Collection, allowing unique, unmatched breadth of landscape analysis and in-depth insights. We hope this report can serve as a useful resource for understanding the current state of knowledge and the importance of raising awareness in the field of rare disease research and development.

## Landscape Analysis of Rare Diseases Research: Publication and Patent Trends from the CAS Content Collection

In this section we present our findings from a comprehensive analysis of more than 530,000 publications (journals and patents) in the field of rare diseases sourced from the CAS Content Collection. Our aim for this analysis was to identify interesting trends in the field such as leading research organizations and scientific journals, as well as identify leading rare diseases in terms of commercial exploration. In addition, we have focused on three of the most voluminous (in terms of journal and patent publications) rare diseases – ALS, HD and MG. Finally, we leveraged CAS REGISTRY, the CAS substance collection, to identify substances across different substance classes co-occurring with the three chosen rare diseases.

**Figure 1:**
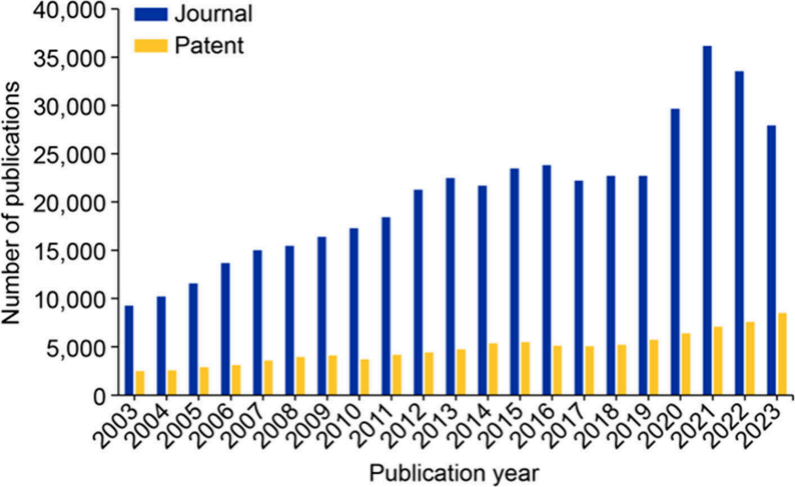
Publications in the field of rare diseases from the CAS Content Collection for the period 2003 to 2023.

To fully capture the field, our subject matter experts utilized more than >650 search terms to ensure both identification of relevant publications in the field as well as capture a wide breadth of pertinent information. The last two decades have seen an increasing interest in rare diseases as shown by the steady increase in journal publications, with a marked and steep increase between 2019 and 2021. Patent publications on the other hand, have increased consistently but at a much more moderate pace (Figure 1).

**Figure 2:**
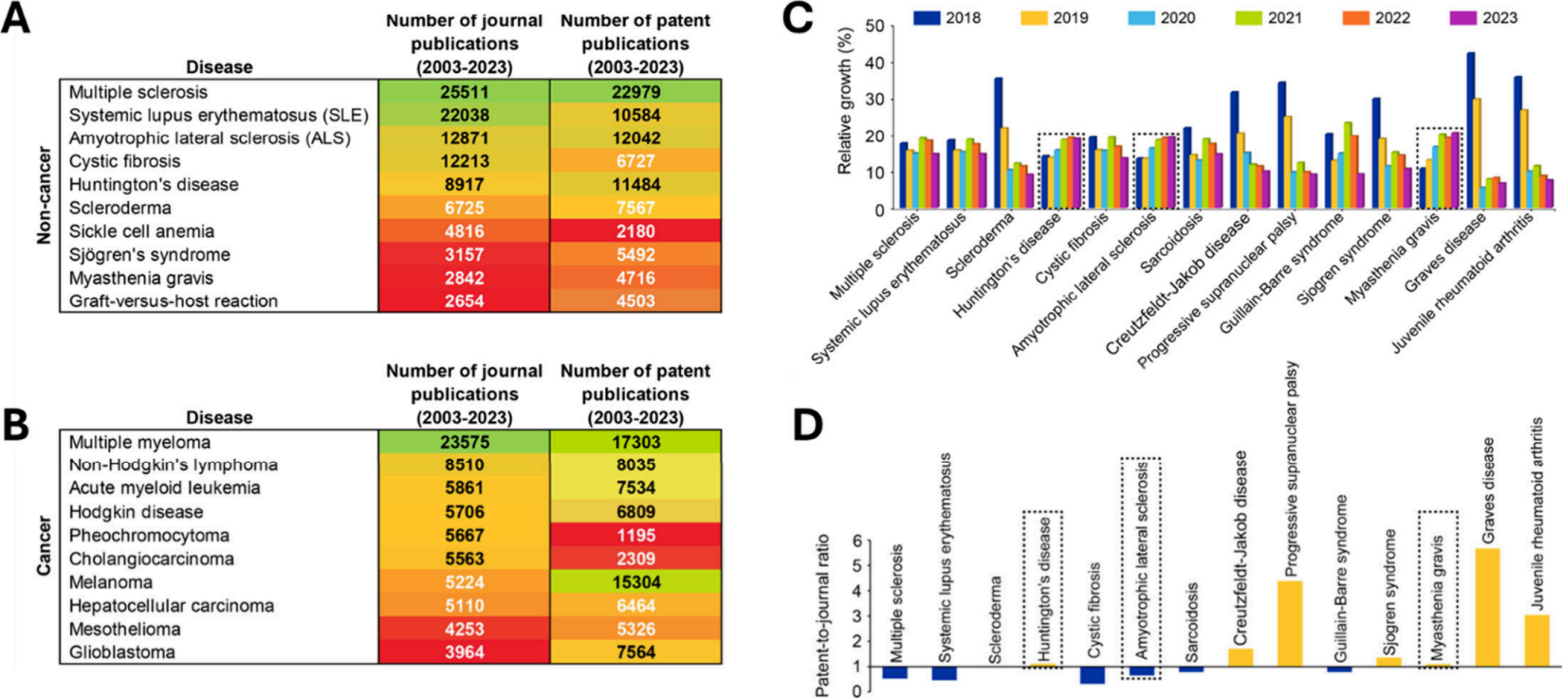
Left panel: Heat maps depicting number of journal and patent publications for leading (A) rare diseases and (B) rare cancers in our data set of rare diseases. Right panel: (C) Number of publications (journal and patent) and (D) patent-to-journal ratios for selected leading rare diseases in our data set. Highlighted in dashed black boxes are the three rare diseases that we analyzed in detail. Data includes journal publications extracted from the CAS Content Collection for the period 2003–2023.

In our data set we identified leading rare diseases and rare cancers based on the number of documents (journals and patents) associated with them using the robust data curation performed by CAS and are shown as heat maps in Figure 2A and 2B – ALS, HD, and MG all feature in the list. Other well-studied rare diseases include autoimmune diseases such as multiple sclerosis,^22^ systemic lupus erythematosus (SLE),^23^ scleroderma,^24^ Sjögren’s syndrome,^25^ and inherited disorders such as cystic fibrosis^26^ and sickle cell anemia.^27^ Among the top 10 well-studied rare cancers, ∼30% are hematological malignancies (blood cancers) such as multiple myeloma,^28^ non-Hodgkin’s lymphoma,^29^ and acute myeloid leukemia (AML).^30^ The list is also populated with rare cancers such as pheochromocytoma, type of neuroendocrine tumor;^31^ cholangiocarcinoma, cancer of the bile duct,^32^ and melanoma, a type of skin cancer.^33^ Other examples of leading rare cancers include hepatocellular carcinoma,^34^ a type of liver cancer, mesothelioma^35^ which is cancer occurring in the tissue surrounding internal organs (mesothelium), and an aggressive form of brain cancer called glioblastoma.^36^

Having identified leading rare diseases and rare cancers in our data set, we wanted to see how interest in these, and other rare diseases have played out over the past few years. To determine this, we plotted the relative increase in publications (journal and patent) for the period 2018–2022 normalized with respect to total publications for that period for each disease (Figure 2C). HD, ALS, and MG show a clear, steady, and consistent increase in publications over the 5-year period. This increase is perhaps most evident for MG with publications nearly doubling between 2018 and 2022. A few rare diseases such as scleroderma,^24^ an autoimmune disease resulting in hardening of skin, Creutzfeld-Jakob disease,^37^ an aggressive neurodegenerative disorder resulting in death, and progressive supranuclear palsy,^38^ neurodegenerative disorder affecting muscles and movement, show waning publications over the years. Sjögren’s syndrome,^25^ Graves’ disease,^39^ and juvenile rheumatoid arthritis^40^ also show a downward trend in publications indicative of decrease in interest from the scientific community. Multiple sclerosis, SLE, sarcoidosis,^41^ and Guillain-Barre syndrome,^42^ all of which involve the body’s immune system, show more or less consistent number of publications with minor fluctuations. A selection of notable patents on ALS, HD, and MG are included in the Supporting Information, Table S5.

Calculating the patent-to-journal ratios for these diseases splits them more or less evenly – with 7 out of 14 analyzed rare diseases having a patent-to-journal ratio greater than 1, indicative of greater commercial interest. Out of the remaining 7, scleroderma has a patent-to-journal ratio of 1 while the rest six have a patent-to-journal ratio of less than 1 indicative of not as much commercial interest (Figure 2D). Out of the three rare diseases that we chose to focus on – HD and MG exhibit a patent-to-journal ratio greater than 1 by a modest extent. On the other hand, ALS has a patent-to-journal ratio of 0.7 indicating greater interest from the scientific community that is yet to translate to commercial prospects – this might also be attributable to the increased interest in ALS following the viral ice bucket phenomenon translating as increased journal publications as a result of increased funding. Among the 14 rare diseases analyzed, progressive supranuclear palsy and Graves disease lead in terms of having more than 4 times as many patents as journal publications (Figure 2D).

Similar analysis with respect to rare cancers reveals increase in publications across the board with all selected rare cancers showing clear, consistent, and rapid increase in publications (Supporting Information, Figure S6A). Of special note are Kaposi’s sarcoma,^43^ cancer affecting the lining of blood vessels and lymph nodes, glioblastoma,^36^ cancer of the brain and/or spinal cord, and thyroid cancer^44^ showing the greatest increase with publications more than doubling between 2019 and 2022 (Supporting Information, Figure S6A). The patent-to-journal ratios indicate seemingly high commercial interest for the analyzed rare cancers with >70% exhibiting patent-to-journal ratios greater than 1 (Supporting Information, Figure S6B). The exceptions to this are multiple myeloma, esophagus cancer^45^ and hepatocellular carcinoma,^34^ a type of liver cancer, with low patent-to-journal ratios (Supporting Information, Figure S6B).

For detailed insights on publication and substance-related analysis please see Figures S1–S9 in Supporting Information.

## Amyotrophic Lateral Sclerosis (ALS), Huntington’s Disease, Myasthenia Gravis

### Amyotrophic Lateral Sclerosis

Amyotrophic lateral sclerosis (ALS), also known as motor neuron disease or Lou Gehrig’s disease, is a rare progressive neurodegenerative disorder affecting the nerve cells in the brain and spinal cord that control voluntary muscle movement.^46−51^ Global estimates of ALS range from 1.9 per 100,000 to 6 per 100,000.^52−54^ ALS shot to public attention because of the viral “ice bucket challenge” in 2014.^55^ Though the exact impact of the viral challenge remains unknown^56−58^ with opinions divided on whether the challenge truly had a real world impact, it is undeniable that it catapulted the disease to the public’s attention. Individuals well-known in their respective fields have been afflicted by this disorder, perhaps one of the most famous ones being the theoretical physicist Stephen Hawking.^59^

An overview and details on the pathogenesis of ALS are included in the Supporting Information.

The genetic background of ALS is multifaceted, involving both familial and sporadic forms of the disease. While the majority of ALS cases are sporadic, meaning they occur without a clear family history, approximately 5–10% of cases are familial and have a known genetic component.^60−65^

Familial ALS (fALS) accounts for a small percentage of ALS cases and is characterized by a clear family history of the disease. In these cases, ALS is inherited in an autosomal dominant manner, meaning that a mutation in a single copy of a specific gene is sufficient to cause the disease.^66^ Several genes have been implicated in fALS, including:

- SOD1 (Superoxide Dismutase 1): Mutations in the SOD1 gene were the first identified genetic cause of ALS. SOD1 encodes an enzyme involved in antioxidant defense, and mutations in this gene can lead to protein misfolding and aggregation, mitochondrial dysfunction, and motor neuron degeneration.^67−69^
- C9orf72 (Chromosome 9 Open Reading Frame 72): Expansion of a hexanucleotide repeat sequence (GGGGCC) within the C9orf72 gene is the most common genetic cause of ALS and frontotemporal dementia (FTD). The exact mechanism by which the repeat expansion leads to neurodegeneration is not fully understood but likely involves RNA toxicity, protein aggregation, and impaired nucleocytoplasmic transport.^70−74^
- TARDBP (TAR DNA-Binding Protein): Mutations in the TARDBP gene, which encodes the TDP-43 protein involved in RNA processing and metabolism, have been identified in fALS cases. Abnormal accumulation of TDP-43 protein aggregates is a pathological hallmark of ALS.^75−77^
- FUS (Fused in Sarcoma): Mutations in the FUS gene, which encodes a DNA/RNA-binding protein involved in RNA processing and transport, have also been implicated in fALS. Like TDP-43, abnormal accumulation of FUS protein aggregates is observed in ALS.^78−80^
- Other Genes: Mutations in other genes, such as VCP (valosin-containing protein), OPTN (optineurin) and SQSTM1 (sequestosome 1) have been identified in rare fALS cases, although their contribution to disease pathogenesis is less well understood.^81−84^

Sporadic ALS (sALS) occurs in individuals with no family history of the disease and is thought to result from a combination of genetic susceptibility and environmental factors. While specific genetic mutations are less common in sALS compared to fALS cases, genome-wide association studies (GWAS) have identified common genetic variants associated with an increased risk of developing ALS. These variants are often found in genes involved in neuronal function, inflammation, and other biological pathways implicated in ALS pathogenesis.^85,86^

**Figure 3:**
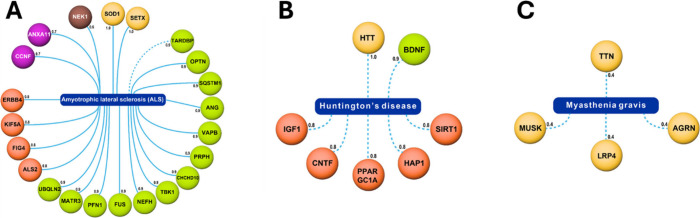
(A) Genes associated with amyotrophic lateral sclerosis (ALS) based on data from the CAS Content Collection. Only genes with an association score of greater than 0.6 and with at least 10 records are shown here. Color corresponds to association score – yellow (1.0), green (0.9), orange (0.8), purple (0.7) and brown (0.6); (B) Genes associated with Huntington’s disease (HD) based on data from the CAS Content Collection. Only genes with an association score of greater than 0.8 and with at least 10 records are shown here. Color corresponds to association score – yellow (1.0), green (0.9) and orange (0.8); (C) Genes associated with myasthenia gravis based on data from the CAS Content Collection. Only genes with an association score 160 of greater than 0.4 and with at least 10 records are shown here. Color corresponds to association score with yellow corresponding to association scores of 0.4. The nature of the line indicates association source with dashed lines indicating a majority of records resulting from text mining.

Genes associated with ALS based on data from the CAS Content Collection are explored in Figure 3A to facilitate the identification of potential therapeutic targets and biological mechanisms underlying disease processes. A comprehensive table, including extensive details on genes, related proteins, expression profiles, function, and other related information, as well as multiple examples, is included in the Supporting Information, Table S1.

ALS exhibits significant genetic heterogeneity, with different genetic mutations associated with distinct clinical phenotypes and disease progression. Additionally, genetic modifiers and environmental factors may influence the penetrance and expressivity of ALS-associated mutations, leading to variability in disease onset, severity, and progression among affected individuals.^65,87,88^ Understanding the genetic basis of ALS is critical for elucidating disease mechanisms, identifying potential therapeutic targets, and developing personalized treatment approaches for individuals with ALS. Ongoing research efforts continue to uncover novel genetic risk factors and pathways underlying ALS pathogenesis, with the ultimate goal of improving outcomes for patients with this devastating neurodegenerative disorder.

#### *The Field of ALS Research Is Experiencing a Surge in Novel Developments*(89−96)

**Gene therapy** targets specific genetic forms of ALS by introducing healthy copies of genes or using tools to silence mutated genes. Although gene therapy is still in early stages for ALS, it holds promise for personalized medicine in the future.

Similar to gene therapy, **antisense oligonucleotides (ASOs)** target mutant RNA transcripts associated with ALS. These short DNA or RNA molecules bind to specific RNA sequences, preventing them from being translated into harmful proteins. Several ASOs are undergoing clinical trials for the treatment of ALS, with some showing early signs of effectiveness in slowing disease progression.

**RNA editing** is an emerging technique, which involves using tools like CRISPR-Cas9 to directly edit mutated genes that cause ALS. While still in preclinical stages, RNA editing has the potential to be a game-changer for ALS if safety and efficacy can be established.

Since the abnormal clumping of proteins in motor neurons is a hallmark of ALS, it is in the focus in ALS treatment. Researchers are exploring various strategies to prevent protein misfolding or clearing these aggregates, potentially slowing neurodegeneration. This includes drugs targeting specific proteins and potential gene therapies aimed at reducing mutant protein production.

**Neuroprotective therapies** aim to protect motor neurons from damage caused by the disease process. Some approaches focus on reducing oxidative stress, inflammation, or other factors contributing to neurodegeneration. Several neuroprotective agents are undergoing clinical trials, but finding effective medications with manageable side effects remains a challenge.

Cerebral dopamine neurotrophic factor (CDNF) is an ER-resident protein expressed in the CNS. A single dose of a novel CDNF variant can improve motor coordination and increase survival in ALS animal models. It can also delay onset of symptoms and protect motor neurons in the spinal cord.^89,90^
A novel treatment strategy uses a small molecule linker, S-XL6, to prevent the separation of the SOD1 protein. The experiments confirmed that this treatment method works in mice for a specific mutation of the SOD1 protein associated with fALS.^91,92^
Neurofilament light chain (NfL) is being investigated as a potential biomarker for ALS. A new drug is under review by the U.S. Food and Drug Administration (US FDA) that is based on its effects on this molecule. The level of NfL in blood and cerebrospinal fluid has been shown to correlate with the speed and severity of ALS progression.^93−96^

The main focus of the ALS research lies on: (i) addressing the underlying genetic causes through gene therapy and RNA editing; (ii) targeting protein misfolding and aggregation to protect motor neurons; (iii) developing neuroprotective therapies to slow disease progression.

### Huntington’s Disease

Huntington’s disease (HD), also known as Huntington’s chorea, is a rare hereditary neurodegenerative disorder characterized by progressive motor dysfunction, cognitive decline, and psychiatric symptoms (the term ‘chorea’ refers to the jerky, unpredictable movements of the muscles in the face, arms and legs that is a prevalent symptom of Huntington’s disease).^97−103^ The estimates are that the global prevalence of HD is ∼2.7 per 100,000 persons.^104^

An overview and details on the pathogenesis of HD are included in the Supporting Information.

The genetic background of HD is primarily characterized by a mutation in the HTT gene, which is located on the short arm of chromosome 4 (4p16.3).^105,106^ The genetic mutation responsible for HD involves an abnormal expansion of a trinucleotide repeat sequence, known as CAG, within the HTT gene. The CAG repeat encodes a polyglutamine tract in the huntingtin protein. In individuals with HD, the CAG repeat is expanded beyond a certain threshold, resulting in an increased number of glutamine residues in the huntingtin protein.^107−110^

HD follows an autosomal dominant pattern of inheritance, meaning that a person who inherits a single copy of the mutant HTT gene from one parent will develop the disease, regardless of whether the other parent carries the mutation. Each child of an affected individual has a 50% chance of inheriting the mutated gene.^105,111^ A notable feature of HD inheritance is anticipation, where the age of onset of symptoms tends to decrease and the severity of symptoms tends to increase in successive generations. This phenomenon is thought to be due to further expansion of the CAG repeat during transmission from one generation to the next.^112,113^ While the presence of an expanded CAG repeat in the HTT gene is necessary for the development of HD, the age of onset and severity of symptoms can vary widely among individuals with the mutation. Some individuals may carry the mutation but never develop symptoms (referred to as incomplete penetrance), while others may have an earlier onset and more severe disease.^114−116^

In addition to the CAG repeat expansion in the HTT gene, other genetic factors may influence the age of onset and progression of HD. Genetic modifiers, such as variations in other genes or nongenetic factors, may interact with the mutant HTT gene to modify disease onset, severity, and progression. Research into genetic modifiers may provide insights into the variability of HD phenotypes and identify potential targets for therapeutic intervention.^117−120^

While the role of HTT in HD is well-established,^121−124^ we wished to collate a comprehensive list of other genes that might play a role in the development of HD. Results from our anlysis are visualized in Figure 3B. Besides HTT, other genes that might play a crucial role in the eitology of HD include BDNF, SIRT1, HAP1, PPARGC1A, CNTF, and IGF1. A comprehensive table, including extensive details regarding genes, related proteins, expression profiles, function, and other diseases, as well as multiple examples, is included in the Supporting Information, Table S2)

Genetic testing for the CAG repeat expansion in the HTT gene can confirm the diagnosis of HD in individuals with symptoms or a family history of the disease. Predictive genetic testing can also be offered to individuals who are at risk of inheriting the mutation but do not yet have symptoms. Genetic counseling is recommended for individuals considering testing to discuss the implications of the test results and the potential impact on themselves and their families.^125−127^ Understanding the genetic basis of HD is essential for accurate diagnosis, genetic counseling, and the development of targeted treatments for this devastating neurodegenerative disorder.

#### *Huntington’s Disease Research Is Exciting and Constantly Evolving*(128−132)

**Gene therapy and gene editing represent** a promising avenue that aims to modify the faulty gene that causes HD. Gene therapy involves introducing healthy copies of the huntingtin gene or tools to silence the mutant gene. Gene editing techniques like CRISPR-Cas9 are being explored to directly edit the mutated gene. These approaches are still in early stages of clinical trials, but they hold immense potential for future treatment.^130^

**RNA silencing** focuses on using small molecules called interfering RNA (RNAi) to silence the mutant huntingtin gene. RNAi essentially targets the mutant mRNA (mRNA) and prevents it from being translated into the harmful protein. Similar to gene therapy, RNA silencing is undergoing clinical trials to assess its safety and effectiveness.^131^

**Antisense oligonucleotides (ASOs)** are another strategy to target mutant huntingtin mRNA. These are short, single-stranded pieces of DNA designed to bind to the mutant mRNA and prevent its translation into protein. ASOs have shown promise in early studies, and researchers are exploring their potential for HD treatment.^130−132^

Researchers are investigating various cellular processes involved in HD progression. This includes studying the role of protein aggregation, oxidative stress, and inflammation. By targeting the **specific molecular** pathways with medications, they hope to slow down neurodegeneration. Several drugs targeting these pathways are undergoing clinical trials.

A novel mouse model expressing the full-length human HTT gene with expanded CAG repeats has been developed. This model accurately replicates the progressive and age-dependent phenotypes of HD, making it a valuable tool for studying disease mechanisms and testing new therapies.^130^
New HD Integrated Staging System groups individuals with HD based on their biological, clinical, and functional characteristics. It is the first staging system developed for a genetic neurological condition and aims to improve the precision of clinical trials and patient care.^130^

**The main focus of novel developments in Huntington’s disease is on:** (i) intervening at the genetic level to silence or modify the mutant gene; (ii) developing medications that can slow or halt disease progression by targeting specific cellular pathways.

### Myasthenia Gravis

Myasthenia gravis (MG) is a rare chronic neuromuscular disorder characterized by weakness and rapid fatigue of voluntary muscles.^133−135^ With the earliest accounts dating back to the late 1600s,^136^ MG is described as an autoimmune disorder affecting neuromuscular junctions.^137^ Thought to arise as a result of the body generating antibodies against the acetylcholine receptor (AChR) or muscle specific kinase,^137,138^ resulting in the immune system mistakenly attacking receptors on muscle cells, particularly at the neuromuscular junction where nerve impulses stimulate muscle contractions,^139−141^ MG leads to muscle weakness and a host of other symptoms.^142,143^ During the Covid-19 pandemic, reports have emerged of onset of MG after SARS-Cov-2 infection.^144,145^ Global prevalence rates range from 150 to 200 cases per 1,000,000 people. The prevalence of MG in the United States is estimated at 14 to 20 cases per every 100,000 people or between 36,000 and 60,000 cases. In Europe, an estimated 56,000 to 123,000 individuals live with MG.^146,147^

An overview and details on the pathogenesis of MG are included in the Supporting Information.

Myasthenia gravis has a complex genetic background, but it is not typically considered a purely genetic disorder like some other conditions. Instead, MG is primarily regarded as an autoimmune disease with genetic predispositions. While the exact cause of MG is unknown, there is evidence suggesting a genetic predisposition to the disease. Certain genetic variations or polymorphisms have been associated with an increased risk of developing MG. These variations are often related to genes involved in immune system function, such as genes encoding human leukocyte antigens (HLAs), specifically the HLA-B8 and HLA-DR3 alleles.^148−154^

MG can sometimes run in families, indicating a potential genetic component.^155^ Family studies have shown that first-degree relatives of individuals with MG have a higher risk of developing the condition compared to the general population. However, the inheritance pattern is usually not straightforward, suggesting the involvement of multiple genetic and environmental factors.^155,156^

Certain HLA alleles, particularly those within the major histocompatibility complex (MHC) region, have been consistently linked to an increased risk of MG.^157^ For example, the HLA-DR3 allele has been associated with MG, especially in individuals with early onset disease and thymic abnormalities.^153^ However, HLA associations alone are not sufficient to explain the development of MG, indicating the involvement of other genetic and environmental factors. In addition to HLA alleles, studies have identified other genetic factors that may contribute to the risk of MG. These include genes involved in immune regulation, such as those encoding cytokines, chemokines, and components of the complement system. Variations in these genes may affect immune function and predispose individuals to autoimmune diseases like MG.^148,158,159^

Using data from the CAS Content Collection, we have put together genes known to have an association with MG (Figure 3C). These genes include AGRN, LRP4, MUSK, and TTN. A comprehensive table, including extensive details regarding genes, related proteins, expression profiles, function, and other diseases, as well as multiple examples, is included in the Supporting Information, Table S3).

It is important to note that the development of MG likely involves complex interactions between genetic susceptibility factors and environmental triggers.^153^ Environmental factors such as infections, medications, and hormonal changes may play a role in triggering the autoimmune response in genetically susceptible individuals. While genetic factors contribute to the susceptibility to MG, the disease’s development is likely multifactorial, involving a combination of genetic predisposition, environmental triggers, and immune dysregulation. Further research is needed to elucidate the specific genetic mechanisms underlying MG and their interactions with environmental factors.^160−162^

#### *Myasthenia Gravis Research Is Actively Exploring New Treatment Options beyond Traditional Medications*(163−166)

**Immunotherapies** target specific aspects of the immune system involved in MG pathogenesis.

- **Complement inhibitors:** The complement system is part of the immune response. These drugs block specific proteins in this cascade, preventing damage to the neuromuscular junction. Examples include eculizumab (CAS RN: 219685–50–4) and ravulizumab (CAS RN: 1803171–55–2), which work by inhibiting the complement system that plays a role in the autoimmune attack on the neuromuscular junction.
- **FcRn blockers:** FcRn receptors are involved in antibody recycling. Blocking these receptors leads to a decrease in circulating antibodies, including those targeting the neuromuscular junction. Efgartigimod (CAS RN: 1821402–21–4) and rozanolixizumab (CAS RN: 1584645–37–3) are new therapies that block the neonatal Fc receptor, reducing the levels of pathogenic antibodies in the blood.
- **B-cell depleting therapies:** B cells are immune cells that produce antibodies. These therapies target and deplete B cells, reducing antibody production associated with MG. One such therapy, rituximab (CAS RN: 174722–31–7), is a medication used for some MG patients, and other B-cell targeting drugs are being investigated.

**Targeted therapies** aim to address specific mechanisms involved in neuromuscular dysfunction:

- **Proteasome inhibitors:** Proteasomes are cellular structures that break down proteins. MG-causing antibodies can impair this process. Drugs like carfilzomib (CAS RN: 868540–17–4) are being explored to see if they can improve neuromuscular function.

**Several other approaches** are under investigation:

- **Autologous stem cell and CAR-T cell therapy:** This involves collecting a patient’s stem cells, treating them, and reintroducing them, potentially resetting the immune system.
- **Thymus-specific therapies:** As the thymus gland may play a role in MG for some patients, researchers are exploring ways to target it more precisely than with thymectomy surgery.

Genes associated with the three spotlighted rare diseases with the association type as causal or contributing, as presented in Figure 3, are summarized in Table 1. Comprehensive Tables, including extensive details regarding genes, related proteins, expression profiles, function, and other diseases, as well as multiple examples for the three diseases, are included in the Supporting Information, Tables S1–S3).

**Table 1:** Genes Associated with ALS, HD, and MG, as Presented in Figure 3^a^

**Disease**	**Gene**
Amyotrophic lateral sclerosis (ALS)	SOD1, SETX, TARDBP, OPTN, SQSTM1, ANG, VAPB, PRPH, CHCHD10, TBK1, NEFH, FUS, PFN1, MATR3, UBQLN2, ALS2, FIG4, KIF5A, ERBB4, CCNF, ANXA11, NEK1
Huntington’s disease (HD)	BDNF, SIRT1, HAP1, PPARGC1A, CNTF, IGF1
Myasthenia gravis (MG)	AGRN, LRP4, MUSK, TTN

a Comprehensive tables for each of the three diseases, including extensive details on the genes and related information, as well as multiple examples, are included in the Supporting Information, Tables S1–S3.

### Landscape Analysis of Data on ALS, HD, and MG in the CAS Content Collection

We focused our attention to the three rare diseases, ALS, HD, and MG, to perform a deeper dive into the publication landscape as reflected in the CAS Content Collection, in order to identify and understand indistinct connections and correlations in these areas.

#### Documents Yearly Growth and Geographic Distribution

**Figure 4:**
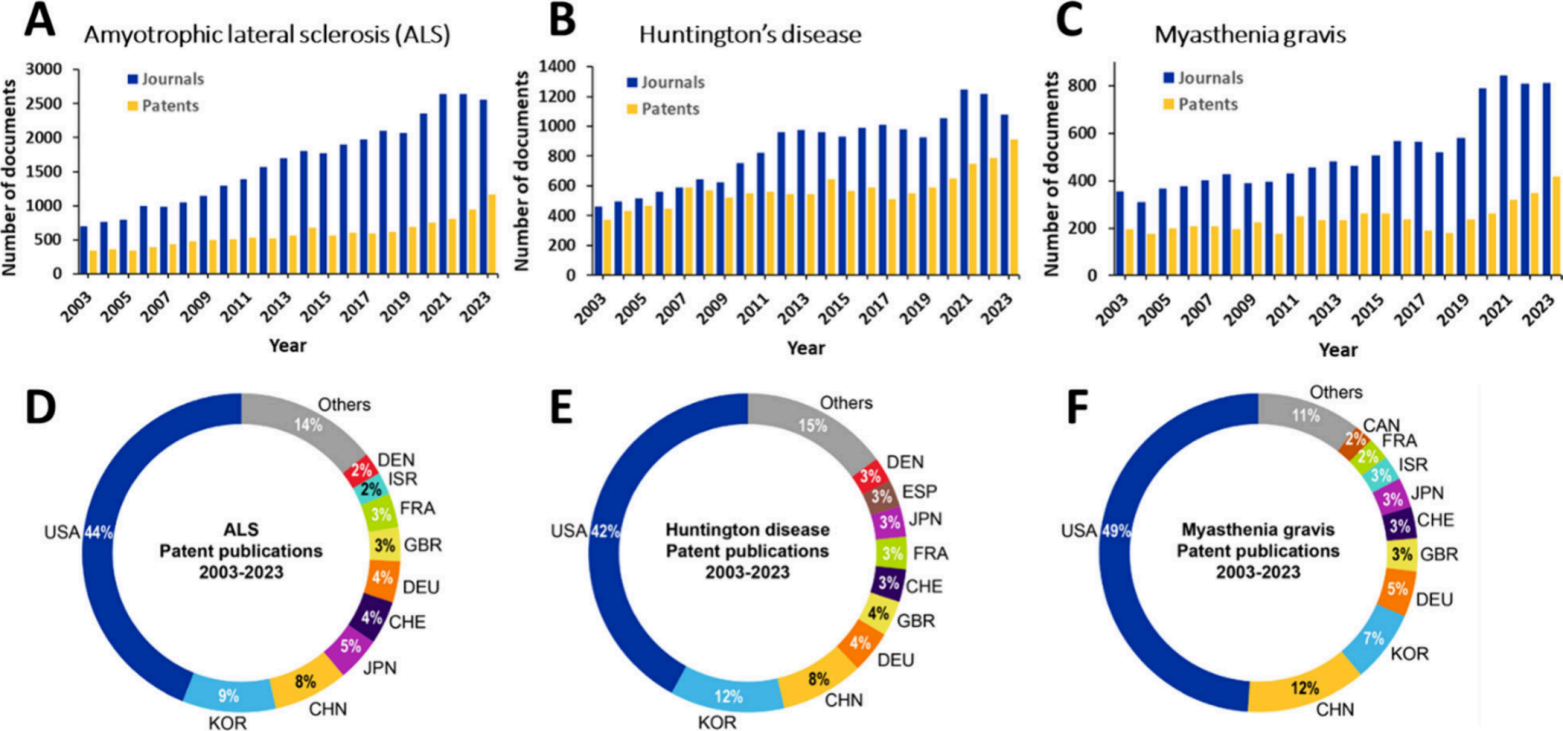
Publications for specific rare diseases: (A) amyotrophic lateral sclerosis (ALS), (B) Huntington’s disease (HD) and (C) myasthenia gravis (MG). Donut graphs showing geographical distribution of patent assignees (both noncommercial and commercial organizations) for each of three diseases: (D) ALS, (E) HD and (F) MG. Countries are indicated by their standard three letter codes – United States (USA), South Korea (KOR), China (CHN), Japan (JPN), Switzerland (CHE), Germany (DEU), United Kingdom (GBR), France (FRA), Israel (ISR), Denmark (DEN), Spain (ESP), Canada (CAN). Data includes journal and patent publications related to each individual rare disease sourced from the CAS Content Collection for 2003–2023. The relative growth in the number of documents related to the three rare diseases in the CAS Content Collection in the last two decades (2003–2023) is shown in the Supporting Information, Figure S9. While MG exhibited the fastest growth in the years 2003–2006, ALS took the lead since 2014, with nearly 8% relative growth in 2023. This increase coincides with and therefore might be attributed to the viral “ice bucket” challenge that started in 2014.

A look at journal and patent publication trends specific to the three diseases in focus indicates the following:

- Publications related to ALS show a steady incline over the last two decades with journal publications increasing by over 20% and patent publications by an impressive ∼70% over 2019 to 2022 (Figure 4A).
- Growth in journal publications related to HD exhibit an upward trend increasing consistently until 2012, followed by a few years of pleateauing and growth in the period 2019–2021. Patent publications grow consistently after 2018 (Figure 4B).
- For MG, journal publications exhibit slow but generally consistent growth. Patent publications on the other hand appear to grow consistently after 2018, after years of pleateauing (Figure 4C).
- For all three diseases journal publications outnumber patent publications.
- Comparison of geographical distribution of patent assignees in the three diseases areas indicate a high degree of overlap, with countries such as the United States (USA), China (CHN), South Korea (KOR), Japan (JPN), Germany (DEU), Switzerland (CHE), the United Kingdom (GBR), and France (FRA) being common among the three. Denmark (DEN) shows up in the top 10 countries or regions for patent assignees in ALS and HD while Israel (ISR) features in the top 10 for ALS and MG (Figure 4D-F).

**Figure 5:**
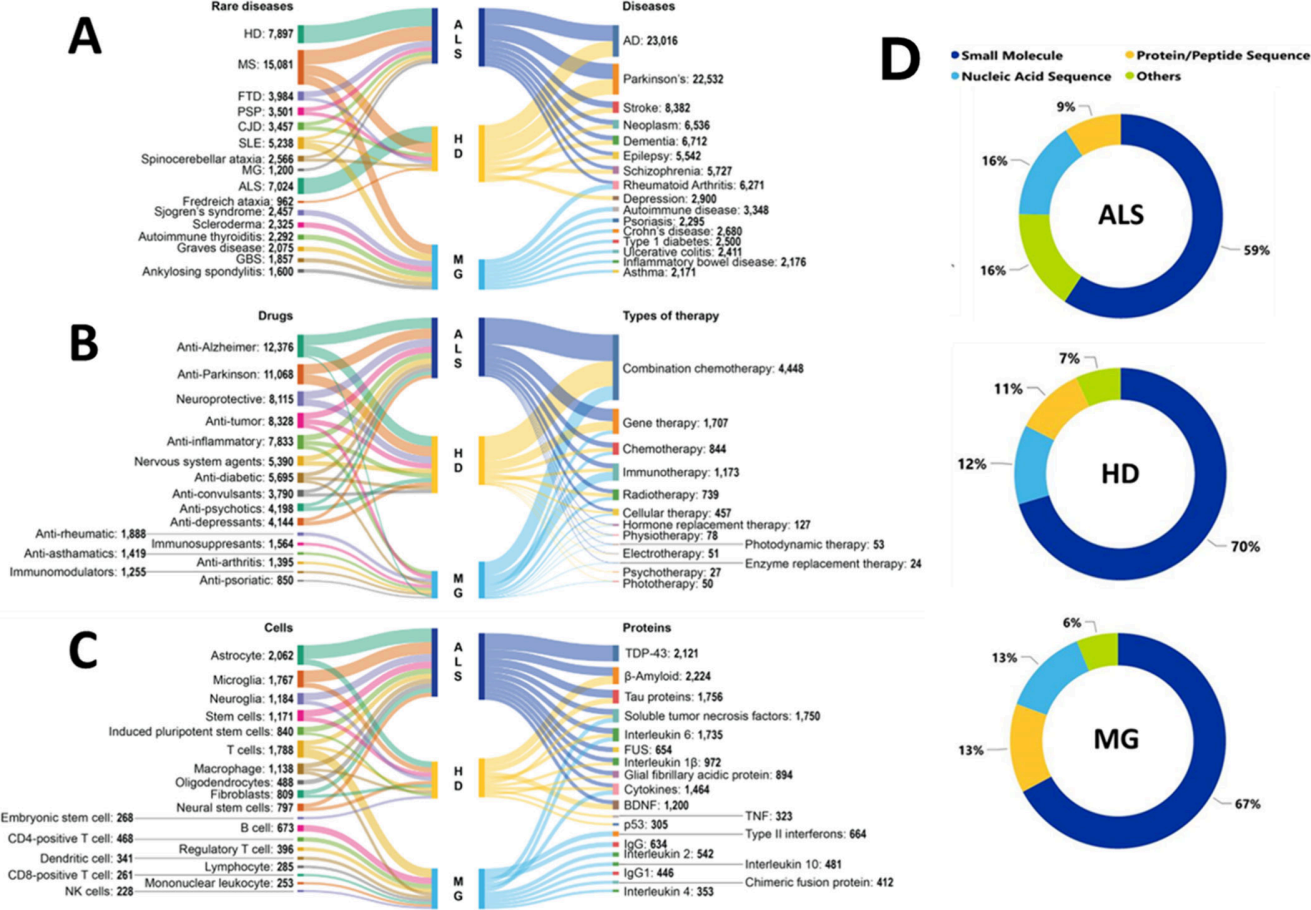
Co-occurrences of amyotrophic lateral sclerosis (ALS), Huntington’s disease (HD), and myasthenia gravis (MG) with various concepts such as (A) other rare and nonrare diseases, (B) types of therapy and drugs used to treat symptoms and (C) cells and proteins. (D) Substance data from the CAS REGISTRY associated with ALS, HD, and MG. Data includes both patent and journal publications sourced from the CAS Content Collection for 2003–2023 in the field of rare diseases. Annual growth for individual classes of substances is provided in Supporting Information Figure S7.

Using our access to robust CAS indexing data, we further explored co-occurrences of the three rare diseases – ALS, HD, and MG, with a host of concepts such as other rare and nonrare diseases (Figure 5A), therapy types and drugs (Figure 5B), as well as proteins and cells (Figure 5C).

#### Disease Comorbidities

##### Amyotrophic Lateral Sclerosis (ALS)

Hypertension and dyslipidemia are the most commonly reported comorbidities.^167^ These results are of particular interest considering the debate related to the protective role of hypertension and other cardiovascular disorders for the prognosis and survival of ALS.^168,169^ The occurrence of autoimmune diseases in ALS patients is frequently reported, but little is known about the related clinical phenotype.^170^ Association of ALS and cancer (overall cancer, as well as certain specific cancers) has been examined, and the results have been ambiguous and inconsistent regarding the risk of cancer in general, and of specific cancers.^171−174^ A study examined co-occurrences of ALS and multiple sclerosis, both associated with upper motor neuron degeneration, checking the possibility for common biological pathways. The study concluded though that rather than a shared biology, the co-occurrences are random, although a common risk factor cannot be excluded.^175^

##### Huntington’s Disease

Examination of comorbidities associated with HD showed depression as the most common, affecting nearly 43% of patients, with females more frequently affected than males.^176^ Other reported comorbidities of HD include dementia (∼38% of patients), urinary incontinence (over 32% of patients), extrapyramidal and movement disorders (over 30% of patients), dysphagia (nearly 30% of patients), and disorders of lipoprotein metabolism (over 28% of patients).^176^ Another study found that the prevalence of comorbidities, especially in the musculoskeletal, cardiovascular, and psychiatric diseases, was higher in patients with HD than in a control group of healthy individuals.^177^ The observed psychiatric comorbidities comprise obsessive-compulsive disorder, depression, insomnia, bipolar affective disorders, dementia, and neurosis.^177^ A recent study reported higher incidence rate of multiple comorbidities, such as obsessive-compulsive disorder, psychosis, communication disorders, depression, anxiety, dementia, and others, in individuals with adult-onset HD than in controls; in patients with juvenile-onset HD, the incidence rates of epilepsy, and acute respiratory symptoms have been found to be higher.^178^ Reports exist of the co-occurrence of HD with ALS.^179^ The number of reported cases of co-occurrence are low, which obstruct systematic observational studies or clinical trials. A single case of multiple sclerosis with comorbid HD has been reported.^180^

##### Myasthenia Gravis

Patients with MG may be associated with autoimmune as well as nonautoimmune comorbidities.^181,182^ Autoimmune comorbidities such as autoimmune thyroiditis, followed by SLE, and rheumatoid arthritis have been reported as the most frequent comorbidities of MS.^183^ Co-occurrence of thymoma MG and late-onset MG with cardiomyositis and subclinical cardiac dysfunction have been reported, however, these conditions have not been considered a significant risk. Lymphomas and some other cancers have been documented with a slightly higher frequency, autoimmune MG does not appear to be a separate cancer risk factor.^183^ MG has been reported in 0.2% of the diagnosed cases of autoimmune thyroid disease. Pernicious anemia, psoriasis, systemic vasculitis, and other disorders have also been reported.^181−184^ The prevalence of autoimmune comorbidity is different for MG subtypes: individuals with early onset MG are more likely to develop an additional autoimmune disease than those with late-onset MG.^183^ Another group of diseases co-occurring frequently with MG are cardiovascular diseases. Arterial hypertension is noted to prevail in patients with MG.^184^ With respect to cancers – lymphoma, breast cancer, and lung cancer have been found more common in the group of MG patients.^182,184^ Mental health disorders such as depression and anxiety are frequent among MG patients. Concurrent MG and ALS have also been registered in many cases.^182,184^

The results of examination of co-occurrences of ALS, HD, and MG terms with other rare and nonrare diseases in the CAS Content Collection are illustrated in Figure 5A. ALS and HD most frequently co-occurs with Alzheimer’s, and Parkinson’s diseases, and multiple sclerosis, as well as between themselves. MG most frequently co-occurs with multiple sclerosis, rheumatoid arthritis, and systemic lupus erythematosus (Figure 5A).

#### Types of Therapy

Combination therapies are the most common type of therapy for the three rare diseases, according to the co-occurrence analysis on data from the CAS Content Collection (Figure 5B). Current research indicates that drugs acting at a single target may be insufficient for the treatment of multifactorial neurodegenerative diseases such as HD and ALS, typified by the coexistence of multiple etiopathologies including oxidative stress, protein misfolding and aggregation, mitochondrial dysfunction, inflammation, and metal accumulation at the sites of neurodegeneration. Clearly, combination drug therapy of neurodegenerative diseases with multifunctional remedies exhibiting diverse biological properties is supposed to have distinct advantage.^185^

ALS and HD frequently co-occur with gene therapy, while for MG, immunotherapy is the second most frequently occurring after combination therapy (Figure 5B). Indeed, with respect to ALS, over 50 genes have been identified as either cause or modifier in ALS and ALS/FTD spectrum disease. Substantial effort has been made to discover pathways underlying the pathogenesis of these gene mutations. Accordingly, targeting etiologic genes to suppress their toxic impacts have been investigated widely, with the major strategies including: removal or inhibition of abnormally transcribed RNA using miRNA or antisense oligonucleotides (ASOs); degradation of abnormal mRNA using RNA interference (RNAi); decrease or inhibition of mutant proteins by using, e.g., antibodies against misfolded proteins; and/or DNA genome editing with methods such as CRISPR or CRISPR/Cas.^186^ The favorable results of these studies have resulted in application of some of these strategies in clinical trials for ALS, especially for C9orf72 and SOD1.^186−189^ Regarding HD, gene therapies are being explored using genetic material to ramp up expression of genes whose functions are declined or are damaged over the course of the disease, to boost the brain and body’s natural resilience against disease progression.^190^ As a monogenic disease, HD is a good target for gene therapy approaches, including the use of programmable endonucleases. A protocol for HTT gene knockout using a modified Cas9 protein (nickase, Cas9n) has been recently tested with promising results.^191^

Regarding MG, immunotherapeutic biologics are emerging as important therapeutic tools. The monoclonal antibody eculizumab (CAS RN: 219685–50–4) has been approved by the US FDA for refractory MG on the basis of a Phase III trial.^192^ Another monoclonal antibody, rituximab (CAS RN: 174722–31–7), is in advanced stage of clinical trials. A selection of newer anti-CD20 antibodies such as ocrelizumab (CAS RN: 637334–45–3), ofatumumab (CAS RN: 679818–59–8), obinutuzumab (CAS RN: 949142–50–1), ublituximab (CAS RN: 1174014–05–1) or inebilizumab (CAS RN: 1299440–37–1) are also being tested.^192^ Enhanced availability of new biologics provides targeted immunotherapies and the chance to develop more specific therapies for MG.

##### Drugs

A co-occurrence search in the CAS Content Collection showed the highest frequency of co-occurrence of ALS and HD with anti-Alzheimer and anti-Parkinson drugs (Figure 5B).

For example, ropinirole (CAS RN: 91374–21–9), a drug used to treat Parkinson’s disease, has showed potential in delaying the progression of ALS.^193^ The multifunctional brain permeable iron chelator M30 was shown to possess neuroprotective activities against various neurodegenerative diseases, such as Alzheimer’s and Parkinson’s disease, and ALS.^194,195^ Recently, allopurinol (CAS RN: 315–30–0) and carvedilol (CAS RN: 72956–09–3), medications used to manage gout and high blood pressure, respectively, were reported to significantly reduce the risk of developing ALS, Alzheimer’s, or Parkinson’s disease.^196,197^

MG frequently co-occurs with immunosuppressant and antirheumatic drugs (Figure 5B). Immunosuppressant medications act to lessen the immune system’s response in order to avoid the immune attacks on NMJs, thereby limiting muscle fatigue. Common immunosuppressive medications used in treating MG include prednisone (CAS RN: 53–03–2), azathioprine (CAS RN: 446–86–6), cyclophosphamide (CAS RN: 50–18–0), methotrexate (CAS RN: 59–05–2), tacrolimus (CAS RN: 104987–11–3), and mycophenolate mofetil (CAS RN: 128794–94–5).^198−202^ Patients with rheumatoid arthritis exhibit high prevalence of MG compared to the general population.^203^ The rheumatoid arthritis drug abatacept (CAS RN: 332348–12–6) has been reported to prevent MG in clinical trial.^202^ Another drug used in the treatment of rheumatoid arthritis, rituximab (CAS RN: 174722–31–7), has been also shown able to reduce the risk of deterioration in MG.^204^

Since there is no cure for these rare diseases, drug repurposing studies have been intensely searching to identify existing drugs that could be repositioned to be used as viable treatment options. As the pharmaceutical development process is both time-consuming and costly, drug repurposing provides a chance to accelerate it by exploring the beneficial effects of agents approved for other disorders. These drugs have established safety profiles, pharmacokinetic description, formulations, dosages, and manufacturing procedures. Recently, *in silico* pharmacology has been widely applied and various computer applications including machine learning and artificial intelligence approaches have been explored in identifying potential drugs for repurposing to various diseases.

There have been number of studies on the potential of old drugs for the treatment of neurodegenerative diseases, including HD and ALS.^205^ Tetrabenazine (CAS RN: 58–46–8) has been developed as antipsychotic drug but has been later repurposed for diseases involving abnormal, involuntary hyperkinetic movements, such as HD.^206^ Another antipsychotic, tiapride (CAS RN: 51012–32–9), has been also tested for the treatment of HD.^207^ Olanzapine (CAS RN: 132539–06–1),^206,208^ risperidone (CAS RN: 106266–06–2),^209^ and quetiapine (CAS RN: 111974–69–7)^210^ are also antipsychotic drugs widely prescribed for the treatment of the motor and behavioral symptoms of HD.^205^ Other examples of drugs under clinical trial to be repurposed for the treatment ALS include the anticancer masitinib (CAS RN: 790299–79–5), the anti-inflammatory ibudilast (CAS RN: 50847–11–5), the antiretroviral triumeq (CAS RN: 1319715–09–7), the anticonvulsant retigabine (CAS RN: 150812–12–7), and the antiestrogen tamoxifen (CAS RN: 10540–29–1).^205^

##### Proteins

A search for co-occurrence with proteins showed that ALS exhibits the highest co-occurrence with TDP-43 (Figure 5C). TDP-43 (TAR DNA-binding protein 43) is a key pathological hallmark associated with ALS and related motor neuron diseases.^211^ Loss of TDP-43 from the nucleus and abnormal accumulation of TDP-43 aggregates in the cytoplasm of affected neurons is a prominent pathological feature observed in the majority (>97%) of cases, particularly in sALS.^212−215^ TDP-43 pathology correlates with disease severity and progression, suggesting a central role in ALS pathogenesis.^216^

While HD etiology has been traditionally associated with abnormalities in the huntingtin protein, recent research has explored the involvement of β-amyloid and tau (τ) protein, typically associated with Alzheimer’s disease, in HD pathology, as reflected by the frequent co-occurrence of these proteins with HD in the CAS Content Collection (Figure 5C). Studies have shown the presence of β-amyloid deposits as well as elevated levels of phosphorylated tau in the brains of HD patients, particularly in regions affected by neurodegeneration.^217−220^

##### Cells

Astrocytes and microglial cells are the most frequently co-occurring cell types with ALS and HD in the rare diseases-related documents of the CAS Content Collection, while MG exhibits high co-occurrence with T-cells and B-cells (Figure 5C). While ALS is traditionally considered a motor neuron disease and HD – a neuronal disorder caused by mutant huntingtin protein (mHTT), emerging evidence suggests that non-neuronal cells, particularly astrocytes, microglia, and other types of neuroglia, play crucial roles in ALS and HD pathogenesis.^221−226^

Astrocytes, the most abundant glial cells in the CNS, are implicated in ALS and HD through various mechanisms.^221,227,228^ Reactive astrocytes can secrete toxic molecules, such as pro-inflammatory cytokines and reactive oxygen species (ROS), contributing to neuronal damage and death. Dysfunctional astrocytes also fail to provide adequate support to neurons, impairing their survival and function. Furthermore, mutations in genes like SOD1 and C9orf72, associated with fALS cases, have been shown to induce astrocyte dysfunction, exacerbating disease progression.^229−231^ Reactive astrocytes in HD exhibit altered morphology and dysregulated function, including impaired glutamate uptake and disrupted calcium signaling.^232−234^ Dysfunction of astrocytes contributes to excitotoxicity, oxidative stress, and neuronal dysfunction in HD. Additionally, astrocytes expressing mHTT can release toxic factors, exacerbating neuronal damage and disease progression.^235−237^

Microglia, the resident immune cells of the CNS, play dual roles in ALS and HD pathology.^221,225,238−240^ While initially recruited to sites of neuronal injury to clear cellular debris and promote tissue repair, microglia can become chronically activated and contribute to neuroinflammation and neurotoxicity. Dysregulated microglial responses, characterized by the release of pro-inflammatory cytokines and neurotoxic factors, have been implicated in motor neuron degeneration. Modulating microglial activation and promoting their neuroprotective functions represent potential therapeutic strategies for ALS and HD.

Other neuroglial cells, such as oligodendrocytes and NG2 glia, may also contribute to ALS and HD pathology.^224,241−243^ Oligodendrocyte dysfunction can disrupt myelination and impair neuronal signaling, contributing to cognitive and motor impairments. NG2 glia, also known as oligodendrocyte progenitor cells, respond to injury and participate in remyelination processes. Dysregulation of these neuroglial cell types may exacerbate neuronal dysfunction and degeneration in ALS and HD.

### Overview of Substance Data

We leveraged our access to the CAS REGISTRY, consisting of data for >250 million substances, and examined substances belonging to diverse classes such as small molecules, protein/peptide sequences, nucleic acid sequences, and others, explored in the documents related to ALS, HD, and MG in the CAS Content Collection (Figure 5D). For all three diseases small molecules constituted the largest fraction of explored substances (Figure 5D). Analysis of the annual growth of the individual substance classes is illustrated in the Supporting Information (Figure S7); while for journal publications the growth in the number of explored substances does not exhibit any particular trend, for patents there is clear upward trend in the number of substances as seen by their relative growths. All three classes of substances (small molecules, protein/peptide sequences, and nucleic acid sequences) show an increase in patent publications indicative of commercial interest in developing these substances as therapeutics. In particular, the nucleic acid sequence and small molecule subclass of substances exhibit a marked increase around 2018–2019 for ALS and HD, respectively. Some specific representative substances from the protein/peptide classes are displayed in Figure S8 in the Supporting Information and include immunosuppressive agents such as cyclosporin (CAS RN: 59865–13–3), chemotherapeutic agents such as actinomycin D (CAS RN: 50–76–0) and peptide hormone amylin (CAS RN: 106602–62–4). Other protein/peptide molecules co-occurring frequently with ALS, HD and MG include antibodies such as the CD-52 directed antibody alemtuzumab (CAS RN: 216503–57–0), VEGF-A directed antibody bevacizumab (CAS RN: 216974–75–3), and α4 integrin directed antibody natalizumab (CAS RN: 189261–10–7), among others.

## Therapeutic Development Pipelines

### Commercial Preclinical Development

**Figure 6:**
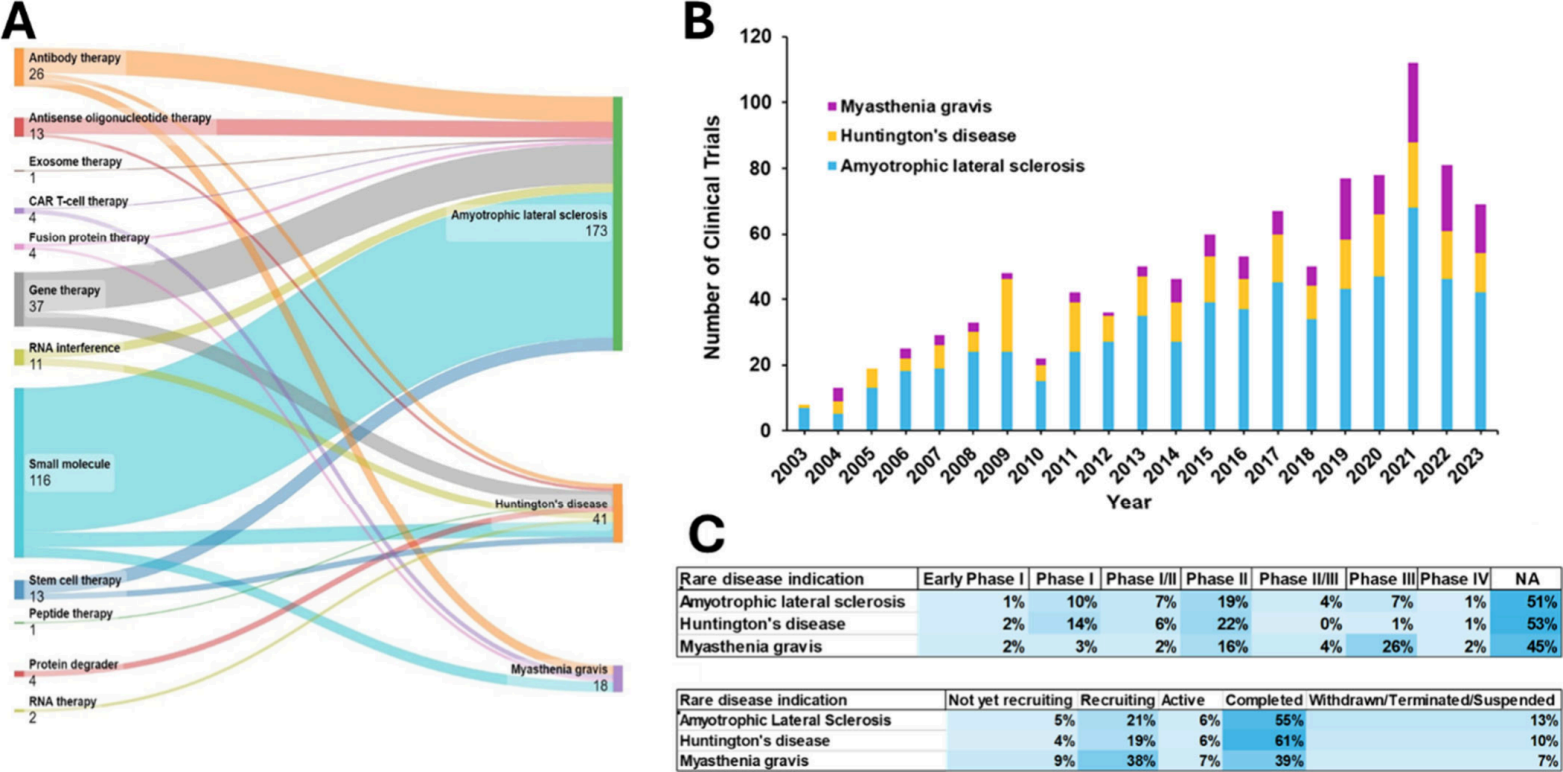
(A) Preclinical drug therapy candidates and their respective rare disease indication currently in the development pipeline; (B) Number of clinical trials by rare disease indications for years 2003–2023; (C) Percentage of rare disease clinical trials in various: phases (upper panel) and statuses (lower panel).

Nearly 250 substances are being researched and developed preclinically for the treatment of ALS, HD, and MG (Table S4 in the Supporting Information lists these compounds, along with their suggestive mechanism of action, therapy type, and the company developing them). The vast majority (74%) of these substances are for the treatment of ALS but therapies for the treatment of HD (18%) and MG (8%) are also in the developmental pipeline (Table S4 in the Supporting Information). A wide range of therapies are being investigated. Small molecule drugs dominate in the total number of drug candidates followed by gene, antibody, RNA, antisense oligonucleotide (ASO), and stem cell therapies, among others (Figure 6A). Small molecule drugs make up about 50% of the drug candidates for ALS and MG, in contrast to only 10% for HD where gene therapy, RNA interference agents, and other biologic treatments dominate (Figure 6A).

### Clinical Trials

Clinical trials researching the treatment of rare diseases ALS, HD, and MG are explored in this section to gain an overall view of the past and current state of clinical development. Over 1,000 clinical trials have been registered on the US National Institutes of Health (NIH) clinical trial website over the last 10 years for these rare diseases. In terms of sheer number, ALS has the highest number of registered clinical trials, followed by HD and MG.^244^Figure 6B shows an oscillating curve for clinical trials of these rare disease, between the years 2013 to 2023. ALS clinical trials gradually increase over the years, while HD stayed more consistent. One note of interest is that the number of clinical trials for MG stay consistent through 2018 and then triple into 2019, maintaining consistency moving forward. One contributing factor to this surge is the US FDA approval of Solaris (eculizumab; CAS RN: 219685–50–4) in 2017, the first for MG, increasing industry-sponsored clinical research to monitor and develop new therapy options for patients with this rare disease.

Further analysis of these rare disease clinical trials reveals that nearly half of all trials for the different indications are not phased (Figure 6C). The phase that contains the next largest group of trials is Phase II studies for ALS and HD and Phase III studies for MG. Nearly half of all clinical trials for ALS, HD, and MG have been completed (Figure 6C). The status with the next largest group of trials is the recruiting status, which is encouraging as new clinical trials are created and carried out to research the treatment of these rare diseases, offering hope to patients worldwide.

Finally, representative clinical trials examining rare disease therapeutics are highlighted in Table 2 categorized by rare disease indication and therapy type. These are examined in further detail below to showcase a variety of promising therapeutic strategies, interventions, and targeted conditions in clinical development along with their status, phase, and any published results.

**Table 2:** Highlighted Rare Disease Therapeutic Clinical Trials

**Therapy type**	**Intervention**	**CAS RN**	**Sponsor, location**	**Status**	**Phase**	**NCT Number**
**Amyotrophic lateral sclerosis**						
Cell therapy	RAPA-501	3037008–26–4	Rapa Therapeutics, USA	Recruiting	Phase II/III	NCT04220190
Gene therapy	AMT-162	2376212–44–9	UniQure Biopharma, Netherlands	Not yet recruiting	Phase I/II	NCT06100276
Small molecule	FB1006	N/A	4B Technologies, China	Recruiting	Phase IV	NCT05923905
Small molecule	Ibudilast (MN-166)	50847–11–5	MediciNova, USA	Completed	Phase I/II	NCT02714036
					Phase II	NCT02238626
				Recruiting	Phase II/III	NCT04057898
Small molecule	Pridopidine	346688–38–8	Prilenia, USA	Recruiting	Phase II/III	NCT04297683
				Completed	Phase II/III	NCT04615923
				Available	Expanded access	NCT05281484
**Huntington’s disease**						
Antisense oligo-nucleotide	WVE-003	3029749–53–6	Wave Life Sciences, USA	Recruiting	Phase I/Phase II	NCT05032196
				Active, not recruiting	Phase II/Phase III	NCT04219241
				Not yet recruiting	Phase III	NCT06097780
Computer based cognitive stimulation	Virtual reality computer simulation	N/A	Santa Creu Hospital, Spain	Active, not recruiting	NA	NCT05769972
Monoclonal antibody	Pepinemab	2097151–87–4	Vaccinex, USA	Completed	Phase II	NCT02481674
Small molecule	SAGE-718	1629853–48–0	Sage Therapeutics, USA	Recruiting	Phase II	NCT05107128
					Phase II	NCT05358821
					Phase III	NCT05655520
Stem cell therapy	NestaCell	N/A	Azidus, Brazil	Active, not recruiting	Phase I	NCT02728115
				Active, not recruiting	Phase II/Phase III	NCT04219241
				Not yet recruiting	Phase III	NCT06097780
**Myasthenia gravis**						
Antigen therapy	CNP-106	N/A	COUR Pharmacetucial, USA	Not yet recruiting	Phase I/II	NCT06106672
Cell therapy	Descartes-08	2784598–58–7	Cartesian Therapeutics, USA	Recruiting	Phase II	NCT04146051
Fusion protein	Telitacicept	2136630–26–5	RemeGen, China	Recruiting	Phase III	NCT05737160
Small molecule	Vemircopan (ALXN2050)	2086178–00–7	Alexion, UK	Active, not recruiting	Phase II	NCT05218096

Therapy types in clinical development highlighted below for the treatment of ALS include cell and gene therapies along with small molecule drugs. An autologous T-cell therapy, RAPA-501 (CAS RN: 3037008–26–4), was developed by RAPA Therapeutics to address the lack of compounds for treatment of neuroinflammation in ALS and is currently recruiting for a Phase II/III clinical trial (NCT04220190). This trial is an expansion cohort that was added to access RAPA-501 cells efficiency in standard-risk ALS patients. In this ongoing clinical trial, RAPA-501 cells were found to be safe, have multiple anti-inflammatory effects, and showed early signs of preserving pulmonary function.^245^ RAPA-501 cells express both the TREG and Th2 transcription factors forkhead box P3 (FOXP3) and GATA-binding protein 3 (GATA3), are enriched for expression of the T-cell homing molecule CD103 and the ATP ectonucleotidase molecules CD39 and CD73.^246^ They also suppress effector T-cell inflammatory molecules and CNS microglial cell inflammatory molecules.^246^ RAPA-501 cells are also available through an expanded access clinical trial (NCT06169176) to patients living with high-risk ALS and are not eligible for other ALS clinical trials. These trials will research therapy feasibility, safety, and efficacy including biomarker measurements for neuroinflammation.

Gene therapy agent, AMT-162 (CAS RN: 2376212–44–9), developed by UniQure Biopharma, is not yet actively recruiting but will soon start a clinical trial, NCT06100276, to research the use of AMT-162 in patients with rapidly progressive ALS and SOD1 mutations. AMT-162, a one-time treatment, is comprised of a recombinant AAVrh10 vector that expresses a miRNA targeting the SOD1 gene. This clinical trial will research the safety and efficacy of AMT-162, evaluating if it will silence the expression of mutant SOD1 and improve the course of ALS.^247^

Small molecule drug FB1006, fully discovered and developed using artificial intelligence (AI), is being advanced as a new potential treatment for ALS. 4B Technologies with collaborative efforts completed the development process of FB1006, from target identification and compound screening to patient enrollment, in less than 2 years.^248^ Phase IV clinical trial NCT05923905 recently completed enrollment of 64 patients which will evaluate the efficacy of safety of FB1006 in the treatment of ALS patients. The trial is being conducted at the Third Hospital of Peking University and is expected to complete double-blind dosing in August 2024, followed by 1-year of clinical observation in February 2025.^248^

The last two small molecules we highlight for the treatment of ALS are both part of the HEALEY ALS Platform trial (NCT04297683). This trial is a perpetual multicenter, multiregimen clinical trial evaluating the safety and efficacy of ALS treatments. There is a single master protocol dictating the conduct of the trials with each regimen sharing placebo patients. The first drug ibudilast (CAS RN: 50847–11–5) is being investigated by MediciNova. A Phase I/II clinical trial NCT02714036 revealed ibudilast to be safe and showed no drug related severe adverse reactions. However, the tolerability was limited due to gastrointestinal side effects, fatigue, and insomnia.^249^ Data from another completed Phase II study NCT02238626 showed ibudilast, when in combination with approved therapy Rilutek (CAS RN: 1744–22–5) showed a marked increase in number of patients who saw no functional decline in six months comparted to Rilutek alone and it also helped increase patient lifespan.^250^ Ibudilast will also be investigated in a Phase II/III clinical trial (NCT04057898) currently recruiting and enrolling up to 230 participants across the USA and Canada to evaluate the efficacy, safety, and tolerability of ibudilast for 12 months followed by a 6-month open-label extension phase. Ibudilast is a glial attenuator that suppresses pro-inflammatory cytokines IL-1β, TNF-α, and IL-6, and may upregulate the anti-inflammatory cytokine IL-10.^251^ It has additionally been shown to be a toll-like receptor 4 antagonist that may contribute to its attenuation of neuroinflammation.^251^ Ibudilast received both fast track^252^ and orphan drug designations^253^ in 2016 from the US FDA for treating ALS.

The second small molecule drug pridopidine (CAS RN: 346688–38–8), also a regimen in the HEALEY ALS Platform trial (NCT04297683), was investigated in a completed Phase II/III clinical trial (NCT04615923). While there were no significant improvements in the primary outcome measures of disease progression and mortality rate, there were positive results reported for secondary outcome measures such as improved respiratory and speech measurements. Improvement in disease progression and neurofilament light levels (biomarker for neuronal injury) for rapidly declining ALS participants who were early in the disease were also seen. Pridopidine is also available through an expanded access clinical trial (NCT06069934) for up to 200 patients with ALS who are ineligible for other clinical trials. Pridopidine is a highly selective sigma-1 agonist. Sigma-1 receptor is highly expressed in the brain and CNS and activation by pridopidine stimulates multiple cellular pathways, including autophagy, which are essential to neuronal function and survival, and may lead to neuroprotective effects.^254^ Prilenia Therapeutics was granted orphan drug designation for pridopidine in 2021 from the US FDA for treating ALS.^255^

Highlighted therapies in clinical development for the treatment of HD include ASOs, cell-based, and monoclonal antibody therapies along with computer based cognitive stimulation and small molecule agents. One such ASO therapeutic is Wave Life Sciences’ gene silencing therapeutic, WVE-003 (CAS RN: 3029749–53–6). Interim results for Phase I/Phase II clinical trial NCT05032196, reveals that a single dose of WVE-003 (30 or 60 mg) led to a mean 35% reduction in mHTT in the cerebrospinal fluid compared to a placebo.^256^ More upcoming trial findings are expected by June 2024. Phase I and Phase II/Phase III clinical trials (NCT02728115 and NCT04219241) assessing the safety and efficacy of Cellavita’s NestaCell, a stem cell therapy derived from immature human dental pulp, are currently active for the treatment of HD. Another Phase III clinical trial (NCT06097780) researching NestaCell is not yet recruiting but has an estimated start date of June 2024 and will also investigate the efficacy and safety of NestaCell. A previous Phase I clinical trial revealed no serious adverse events and improved HD motor symptoms with the use of NestaCell in the treatment of HD.^257^

Another treatment of HD utilizing a computer based cognitive rehabilitation program is being researched by the Santa Cre Hospital in Spain. Researchers are currently recruiting for their study (NCT05769972) to examine the use of this method in patients with HD with expectations that the program will have a greater beneficial effect on the cognitive status of HD patients compared to control modalities such as music therapy. Monoclonal antibody therapeutic Pepinemab (CAS RN: 2097151–87–4), a semaphorin 4D blocking antibody developed by Vaccinex, was researched in a Phase II clinical trial (NCT02481674) to determine safety, tolerability, pharmacokinetics, and efficacy. While this trial did not meet its primary end points, it did have a favorable safety profile, showed a reduction in brain atrophy, and improvement in decline in brain metabolic activity that is typically seen in HD progression.^258^

Lastly for the treatment of HD, Sage Therapeutics’ small molecule drug SAGE-718 (CAS RN: 1629853–48–0) is currently recruiting for their Phase II/Phase III clinical trials. These trials (NCT05107128, NCT05358821, and NCT05655520) will investigate the safety, tolerability, and efficacy of these drugs for the treatment of HD. SAGE-718, a NMDA receptor positive allosteric modulator has completed initial single and multiple ascending dose clinical studies, where it demonstrated efficacy in disease-relevant populations.^259^ In addition, SAGE-718 was granted both FDA Fast track designation^260^ in 2022 and FDA Orphan Drug Designation in 2023.^261^

Therapy types in clinical development highlighted for the treatment of MG include antigen, cell, and fusion protein therapies along with small molecule drugs. COUR Pharmaceutical is developing CNP-106, an antigen specific therapeutic designed to prevent immune mediated neuromuscular destruction and aims to reprogram the immune system to address the immunological root cause of MG.^262^ The not yet recruiting Phase I/II clinical trial (NCT06106672) will enroll up to 54 adult patients and assess the treatment’s safety, tolerability, pharmacological properties, and efficacy. Descartes-08 (CAS RN: 2784598–58–7), a mRNA CAR T-cell therapy expressing a chimeric antigen receptor directed to B-cell maturation antigen is currently recruiting for an ongoing Phase II clinical trial (NCT04146051). Results from the Phase IIa portion of the study revealed that Descartes-08 is well tolerated and participants saw meaningful improvement in MG disease scorings.^263^ Descartes-08 was granted Orphan Drug Designation by the US FDA for the treatment of MG in 2024.^264^

RemeGene is currently recruiting for its Phase III clinical trial (NCT05737160) investigating fusion protein Telitacicept (CAS RN: 2136630–26–5) for the treatment of MG. Telitacicept is constructed with the extracellular domain of the transmembrane activator and calcium modulator and cyclophilin ligand interactor receptor and the fragment crystallizable domain of immunoglobulin G.^265^ Telitacicept targets two cell-signaling molecules critical for B-lymphocyte development: B-cell lymphocyte stimulator and a proliferation inducing ligand, which allows it to effectively reduce B-cell mediated autoimmune responses. Results from a previous Phase II study showed that Telitacicpet improved MG symptoms and had a good safety profile.^265^ Lastly, we look at a small molecule factor D inhibitor developed by Alexion Pharmaceuticals. ALXN2050 (CAS RN: 2086178–00–7) is currently being investigated in an active Phase II clinical trial (NCT05218096) for its ability to improve the disease symptoms and the daily life of people with MG along with its safety. Seventy patients are enrolled with a study completion date of late 2025, results with be forthcoming soon.

### FDA Approved Therapeutic Agents

While there is no current cure for ALS, HD, or MG, there are treatments to slow disease progression and treat symptoms. Table 3 examines the US FDA approved treatments for these rare diseases along with their CAS RN, therapy types, mechanism of action, and company information. Small molecule drugs dominate the approved drugs for both ALS and HD, with biologic therapies such as monoclonal antibodies, antibody fragments, and peptide therapy making up the approved treatments for MG.

**Table 3:** US FDA Approved Drugs for the Treatment of Specified Rare Diseases^a^

**Drug**	**Therapy type**	**CAS RN**	**Rare disease indication**	**Mechanism/notes**	**Company, location**
Exservan (riluzole)	Small molecule	1744–22–5	Amyotrophic lateral sclerosis	Glutamate signaling blocker/oral film formulation	Mitsubishi Tanabe Pharma America, USA
Nuedexta (dextromethorphan hydrobromide and quinidien sulfate)	Small molecule	2445595–41–3	Amyotrophic lateral sclerosis	Sigma-1 receptor agonist, NMDA receptor antagonist	Otsukac America Pharmaceutical, USA
Qalsody (tofersen)	Gene therapy	2088232–70–4	Amyotrophic lateral sclerosis	Targets SOD1 mRNA to reduce SOD1 protein production	Biogen, USA
Radicava (edaravone)	Small molecule	89–25–8	Amyotrophic lateral sclerosis	Free radical scavenger	Mitsubishi Tanabe Pharma America, USA
Relyvrio (sodium phenylbutyrate and taurursodiol)	Small molecule	2436469–04–2	Amyotrophic lateral sclerosis	Small molecule chaperone and Bax inhibitor/withdrawn 2024	Amylyx, USA
Rilutek (riluzole)	Small molecule	1744–22–5	Amyotrophic lateral sclerosis	Glutamate signaling blocker/oral tablet formulation	Sanofi, USA
Tiglutik (riluzole)	Small molecule	1744–22–5	Amyotrophic lateral sclerosis	Glutamate signaling blocker/oral thickened suspension	ITF Pharma, USA
Austedo (deutetrabenazine)	Small molecule	1392826–25–3	Huntington’s disease	VMAT2 inhibitor	Teva Pharmaceutical, Israel
Austedo XR (deutetrabenazine)	Small molecule	1392826–25–3	Huntington’s disease	VMAT2 inhibitor/extended release formulation	Teva Pharmaceutical, Israel
Ingrezza (valbenazine)	Small molecule	1025504–45–3	Huntington’s disease	VMAT2 inhibitor	Neurocrine Biosciences, USA
Xenazine (tetrabenazine)	Small molecule	58–46–8	Huntington’s disease	VMAT2 inhibitor	Lundbeck Pharmaceuticals, Denmark
Rystiggo (rozanolixizumab-noli)	Monoclonal antibody	1584645–37–3	Myasthenia gravis	Targets FcRn to prevent IgG recycling	UCB, USA
Soliris (eculizumab)	Monoclonal antibody	219685–50–4	Myasthenia gravis	Complement factor C5 inhibitor	Alexion, UK
Ultomiris (ravulizumab-cwvz)	Monoclonal antibody	1803171–55–2	Myasthenia gravis	Complement factor C5 inhibitor	Alexion, UK
Vyvgart (efgartigimod alfa-fcab) intravenous injection	Antibody fragment	1821402–21–4	Myasthenia gravis	Fc receptor blocker	Argenx, Netherlands
Vyvgart Hytrulo (efgartigimod alfa and hyaluronidase-qvfc) subcutaneous injection	Antibody fragment	1821402–21–4	Myasthenia gravis	Fc receptor blocker	Argenx, Netherlands
Zilbrysq (zilucoplan)	Peptide therapy	1841136–73–9	Myasthenia gravis	Complement factor C5 inhibitor	UCB, USA

a Source: The CAS Content Collection. For a list of notable patents in the field of rare diseases – ALS, HD and MG – published in recent years (2020 to 2023) please see Table S5 in the Supporting Information.

There are 13 unique drugs currently approved by the US FDA for the treatment of these rare diseases. Three of these compounds have multiple approved formulations, as well. One example, the drug riluzole (CAS RN: 1744–22–5) has three different formulations approved. The first being an oral tablet, there is also an oral film, and a thickened suspension available for patients dealing with muscle tone and swallowing issues. One of the approved drugs for the treatment of ALS, Relyvrio (CAS RN: 2436469–04–2), has been recently discontinued. Amylyx Pharmaceuticals has started the process with the US FDA of discontinuing authorizations for Relyvrio and removing it from the market.^266^ While Relyvrio is generally safe and well tolerated, it unfortunately failed to meet primary and secondary end points in a Phase III clinical trial (NCT05021536).^266^

Capital investment data related to ALS, HD, and MG over the past 10 years are included in the Supporting Information.

## Outlook and Perspectives

In the vast landscape of medical conditions, rare diseases occupy a unique and often overlooked niche. Defined by their low prevalence, these disorders collectively affect millions worldwide,^8,9^ presenting a multitude of challenges to patients, healthcare professionals, and researchers. Moreover, rare diseases offer a window into the diversity of human health and the complexity of biological systems. Each condition represents a unique manifestation of genetic, environmental, or infectious factors, often with distinct clinical presentations and treatment challenges. From rare genetic disorders like cystic fibrosis and HD to autoimmune conditions like lupus, MG, and rare cancers, the spectrum of rare diseases encompasses a broad array of pathologies.

Significant roadblocks remain on the path to progress in rare disease research and care. The small size of patient populations presents challenges for conducting robust clinical trials, leading to limited evidence-based treatment options. Furthermore, the fragmented nature of rare disease research and healthcare delivery can impede collaboration and knowledge sharing. Fragmented approaches to research and care may limit opportunities for interdisciplinary collaboration and hinder the translation of scientific discoveries into clinical practice. Commercial incentives for developing treatments for rare diseases have been relatively low accounting for low interest from pharmaceutical industry.

Despite the challenges, there is reason for optimism in the rare disease landscape. Advances in genomics, molecular biology, and precision medicine hold promise for improved diagnosis and targeted therapies. Technologies such as next-generation sequencing have revolutionized our ability to identify genetic mutations underlying rare diseases. Whole exome sequencing (WES)^267−269^ and whole genome sequencing (WGS)^270,271^ have become indispensable tools for unraveling the genetic basis of rare diseases, facilitating personalized medicine approaches and targeted therapies. Research into rare diseases has uncovered a plethora of novel disease mechanisms, shedding light on fundamental biological processes and pathways underlying human health and disease. Insights gained from studying rare diseases have broad implications for understanding more prevalent disorders and have led to the identification of druggable targets and therapeutic strategies. The general public is also becoming increasingly aware of the existence of these rare diseases, driving the overall interest up.

Despite the inherent challenges in developing treatments for rare diseases, there have been significant advancements in therapeutic innovations. From small molecule drugs to gene and cell-based therapies, researchers are exploring diverse modalities to address the unmet medical needs of individuals with rare diseases. Collaborative initiatives, regulatory incentives, and patient-centered trial designs are accelerating the translation of scientific discoveries into clinically meaningful interventions. AI is also being utilized from drug target discovery to the clinical pipeline, and beyond, to speed pharmaceutical progress and development. The sharing of data and resources through collaborative platforms and consortia has emerged as a cornerstone of rare disease research. Initiatives such as the Global Alliance for Genomics and Health (GA4GH)^272^ and the Undiagnosed Diseases Network (UDN)^273^ facilitate data exchange, harmonize standards, and foster interdisciplinary collaborations, thereby maximizing the impact of research efforts and empowering patients and families with rare diseases.

Table S6 in the Supporting Information summarizes specifically the outlook and perspectives on ALS, HD, and MG.

Looking ahead, continued investment in rare disease research, infrastructure, and policy initiatives is critical for overcoming existing challenges and maximizing the potential of scientific advancements to improve the lives of individuals with rare diseases. Multidisciplinary collaborations and inclusive research practices are needed to effectively address the complex and evolving landscape of rare diseases.

## Supporting Information Available

Additional methodology for genetic assessment and figures and descriptions pertaining to the landscape analysis of rare disease research utilizing the CAS Content Collection. Pathogenesis descriptions of ALS, HD, and MG and a capital investment analysis. Tables consisting of associated genes, therapeutic drug candidates in preclinical development, notable patents, and outlooks and perspectives for ALS, HD, and MG (PDF)
