# Supplementary material for: Rare Diseases, Spotlighting Amyotrophic Lateral Sclerosis, Huntington’s Disease, and Myasthenia Gravis: Insights from Landscape Analysis of Current Research
Source: Biochemistry. 2025 Apr 1;64(8):1698–719. doi: 10.1021/acs.biochem.4c00722 (PMC12004453; doi:10.1021/acs.biochem.4c00722)
Supplement: Supplementary file 1 — bi4c00722_si_001.pdf [file bi4c00722_si_001.pdf]

Supporting Information for:

Rare diseases, spotlighting amyotrophic lateral sclerosis, Huntington's disease, and myasthenia gravis: Insights from landscape analysis of current research

Kavita A. Iyer†[1], Rumiana Tenchov†[1], Janet M. Sasso†[1], Krittika Ralhan[2], Jyotsna Jotshi[2], Dmitrii Polshakov[1], Ankush Maind[2], Qiongqiong Angela Zhou*[1]

[1]CAS, A Division of the American Chemical Society, Columbus, Ohio 43210, United States
[2]ACS International India Pvt. Ltd., Pune 411044, India

† Authors K.A.I., R.T., and J.M.S. contributed equally to this paper
*Corresponding author, email: qzhou@cas.org

Supporting Information

Methodology for genetic assessment of rare diseases utilizing the CAS Content Collection

Data Source

This study utilized the comprehensive CAS Content Collection to access disease-related genes associated with myasthenia gravis (MG), amyotrophic lateral sclerosis (ALS), and Huntington's disease (HD). The database aggregates pertinent information from various sources and provides a repository of biomarkers linked to physiological and pathophysiological conditions. Genetic data retrieval was performed using the database's query functionalities, allowing for targeted selection of genes associated with each disease.X queries can be inserted here, if required.

Selection Criteria

Genes associated with MG, ALS, and HD were selected based on a scoring metric termed the "association score." The association score is characteristic of each association drawn between the disease and mentioned gene. The score ranges from 0 to 1 and considers the number and type of sources and the number of publications supporting the association (as defined by the data source). [1]
Genes with association scores above a predefined threshold were prioritized for further analysis and visualization. The threshold for inclusion was determined based on the distribution of association scores and the desired scope of the study.

Visualization Approach

A visualization approach was employed to provide a comprehensive landscape of the genes implicated in MG, ALS, and HD. Genes meeting the selection criteria were subjected to visualization techniques aimed at representing their associations with the respective diseases. The association scores of selected genes were visually represented to convey the strength of their associations with the respective diseases. This approach allowed for the identification of genes with the highest relevance to disease pathophysiology.

Conclusion

In summary, this study employed a database-driven approach to visualize disease-related genes associated with MG, ALS, and HD. The utilization of association scores and visualization techniques provided a comprehensive landscape of genes implicated in disease pathogenesis, facilitating the identification of potential therapeutic targets and biological mechanisms underlying disease processes.

Landscape analysis of rare diseases research: Publication and patent trends from the CAS Content Collection

Leading scientific organizations

To identify leading research organizations actively involved in publishing scientific content related to rare diseases, we first ranked them by the volume of journal articles followed by calculating the average number of citations per publication, a viable indicator of the scientific impact for published work. The top 100 research organizations in terms of number of journal articles were then ranked by the average number of citations per publication. In terms of geographic distribution, ~67% of the leading 15 research institutions originated from the United States (USA). The remaining 35% consisted of research organizations from Canada (CAN) and the United Kingdom (GBR) accounting for 20% and 13%, respectively. Within the USA, key universities identified were University of Washington, University of Pennsylvania, Johns Hopkins University, University of California, Washington University, and University of Texas. Other important research organizations from the USA were Harvard Medical School, which ranked 1st among the leading 15 organizations, the US government run agency National Institute of Health (NIH), as well as the Mayo Clinic, and Baylor College of Medicine (Figure S1).

Examples of recent journal articles published by researchers at Harvard Medical School include articles related to HD,[2, 3] MG,[4] ALS,[2, 3] as well rare tumors and cancers such as thymomas,[5] multiple myeloma,[4] and thyroid cancer.[6-8] Similarly, examples of research output from other leading US-based organizations revolve around rare diseases such as multiple sclerosis,[9, 10] ALS,[11-13] systemic lupus erythematosus (SLE),[14, 15] sickle cell anemia,[16, 17] among others and rare cancers such as hematological malignancies including multiple myeloma,[5] acute myeloid leukemia (AML),[18, 19] and non-Hodgkin's lymphoma.[20, 21] Research organizations originating in Canada and those originating in the United Kingdom (GBR) also appear to be directing research efforts towards rare diseases such as multiple sclerosis,[22-25] HD,[26-29] SLE,[30-33] and ALS,[34, 35] among others.

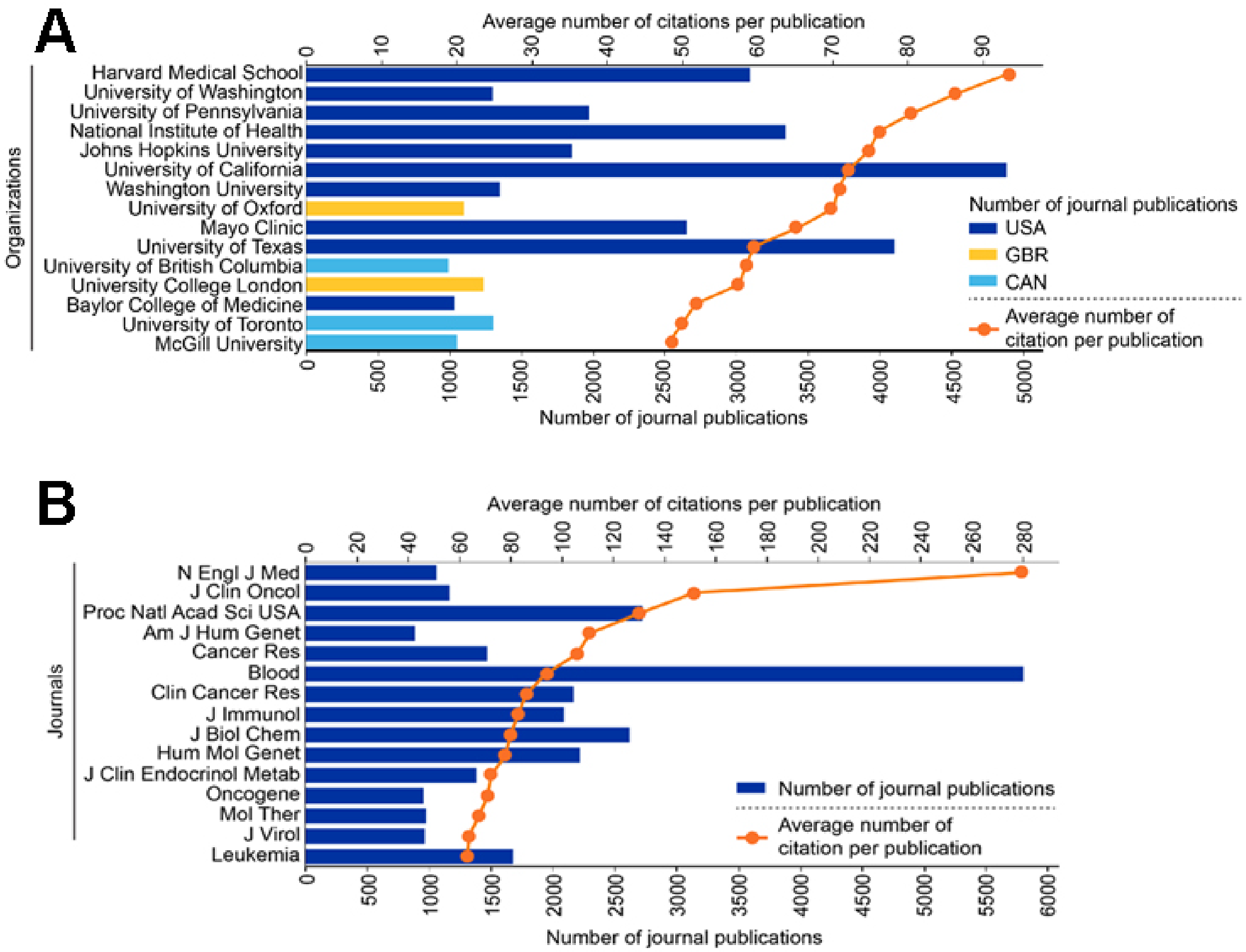

Figure S1. (**A**) Leading research organizations and (**B**) Leading scientific journals in the field of rare diseases based on data from the CAS Content Collection for the period 2003 to 2023. Ranking was based on total number of journal publications and average number of citations per publication, an indicator of publication impact.

Scientific journals

Using a similar methodology, we identified leading scientific journals in the area of rare diseases (Figure S2B). The leading scientific journals identified include the New England Journal of Medicine (*New Engl J Med*), Journal of Clinical Oncology (*J Clin Oncol*), and Proceedings of the National Academy of Sciences (*Pro Natl Acad Sci USA*), among others (Figure S1B). The scientific journal New England Journal of Medicine has half as many journal articles as the Proceedings of the National Academy of Sciences but two times the average number of citations per publication. When looked at from the lens of sheer volume of publications or number of citations a somewhat different group of scientific journals emerge. There is a moderate degree of overlap between the two lists (number of journal publications and number of citations) with scientific journals such as Blood, PLOS One, Proceedings of the National Academy of Sciences, Journal of Biological Chemistry (*J Biol Chem*), and Human Molecular Genetics (*Hum Mol Genet*) featuring in both lists (Figure S1). However, the order of appearance differs, for instance, while the open-access journal PLOS One leads in terms of sheer number of publications (nearly 2X fold higher than the journal Blood, the 2nd leading journal), it is 3rd in terms of number of citations.

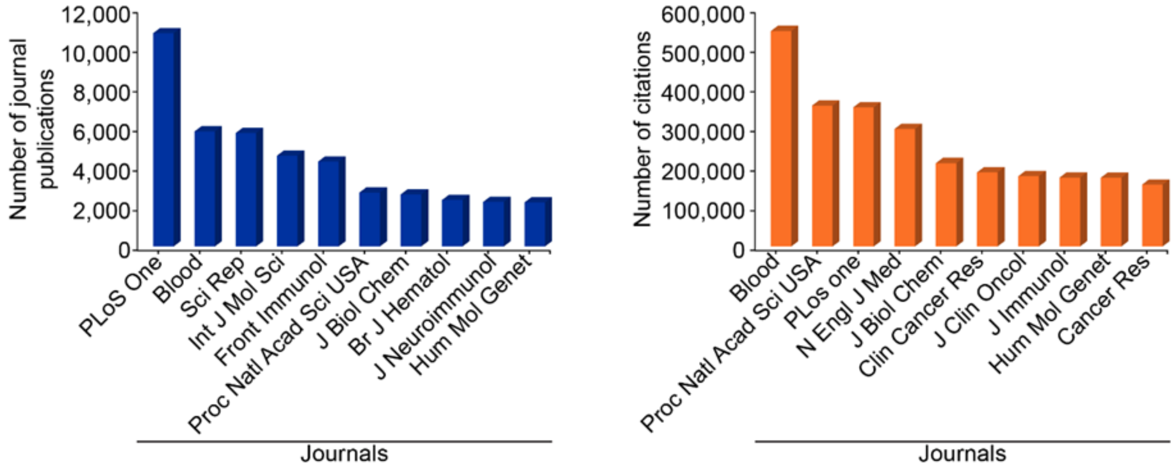

Figure S2. Leading research journals in the field of rare diseases based solely on total number of (**A**) journal publications and (**B**) citations.

A few highly cited journal articles published in the New England Journal of Medicine revolve around rare cancers such as melanoma,[36, 37] multiple myeloma,[38] and AML[36, 37] as well as other rare diseases such as SLE,[38] multiple sclerosis,[39, 40] and cystic fibrosis.[41] The 2019 article titled "Targeting huntingtin expression in patients with Huntington's disease"[42] by researchers in the United Kingdom and published in the New England Journal of Medicine described results of a Phase I/IIa trial for an oligonucleotide designed by Ionis Pharmaceuticals and F. Hoffmann–La Roche to inhibit mRNA of HTT, the main gene responsible for HD and has been cited more than 400 times. Another example of a highly cited article was "Neurotoxic reactive astrocytes are induced by activated microglia" published in 2017 in the journal Nature and described the role of A1 astrocytes in neuronal cell death and their abundance in HD, ALS, and other neurodegenerative disorders.[43] Similar well-cited publications for MG include a review article published in the New England Journal of Medicine.[44]

Using volume of research and number of citations shows a different set of leading journals (Figure S2).

Geographic distribution

Geographical distribution of patent assignees in the field of rare diseases indicates a high degree of overlap in leading countries/regions with 7 out of 10 being common between commercial as well as non-commercial entities – United States (USA), China (CHN), South Korea (KOR), France (FRA), Germany (DEU), Japan (JPN), Denmark (DEN) (Figure S3). Spain (ESP), Italy (ITA), and Belgium (BEL) appear to have greater non-commercial presence while the inverse appears to be true for the United Kingdom (GBR) and Israel (ISR). Overall, the USA contributes 31% and 40% to non-commercial and commercial patents, respectively, the highest by a country/region. This is followed by China (CHN) which appears to have a more comparable commercial and non-commercial contribution.

Among the non-commercial organizations, more than 65% of the leading organizations originate from the United States with the University of California leading overall (Figure S3A). The remaining consist of two research organizations each from South Korea (KOR) and China (CHN) with the L'Institut national de la santé et de la recherche médicale (INSERM) being the only

French organization featuring in the top 15 non-commercial organizations. In recent years, these leading organizations appear to be involved in research across a variety of rare diseases such as SLE,[14] HD,[14] multiple sclerosis,[45-47] and the more obscure ones such as disabling pansclerotic morphea[48] and Von Hippel-Lindau disease.[49] Patents filed by researchers from the University of California in recent years (2015 onwards) cover a diverse group of ailments including developing small molecule inhibitors targeting various proteins and potentially useful in the treatment of ALS (WO2023086603,[50] WO2022104148[51]), using a SARS-Cov-2 pseudoviral delivery system for therapeutic transgene as potential treatment for HD (WO2022165538A1[52]), and development of GABA positive allosteric modulators (WO2018236955[53]) and peptide-based inhibitors for treatment of MG, (WO2018049053[54]).

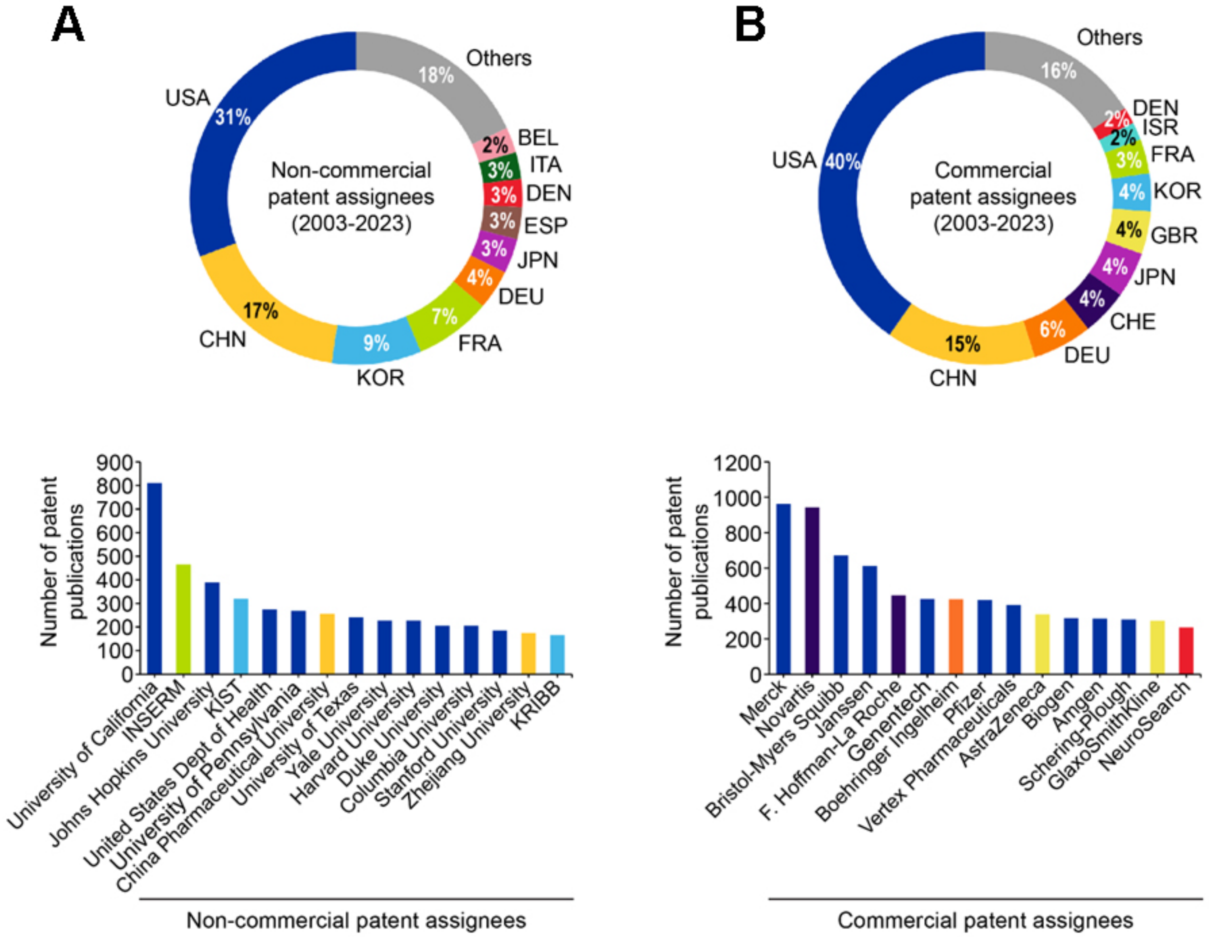

Figure S3. Leading organizations in the field of rare diseases based on patent publication data from the CAS Content Collection for the period 2003 to 2023. (**A**) Donut and (**B**) bar charts showing geographical distribution and leading organizations, respectively. Entities were separated into non-commercial and commercial organizations. Bars colored to correspond to countries or regions shown in the donut charts. Countries or regions represented by their standard three letter codes – United States (USA), China (CHN), South Korea (KOR), France (FRA), Germany (DEU), Japan (JPN), Spain (ESP), Denmark (DEN), Israel (ISR), Italy (ITA), Belgium (BEL), Switzerland (CHE), and United Kingdom (GBR).

Guided by our identification of leading commercial patent assignees we analyzed in greater detail patents by the top 6 companies (Figure S3B) to understand the spread of commercial interest across different rare diseases and shown as heat maps in Figures S4 and S5 based on CAS indexing. Rare diseases such as multiple sclerosis,[55] SLE,[56] scleroderma, and HD appears to have high commercial interest across the six companies we chose to focus on as seen by the higher number of patent publications. In contrast, examples of rare diseases with apparent low commercial interest include iridocyclitis, familial Mediterranean fever,[57] Rett syndrome[58] and pseudoaldosteronism[59] (Figure S4). Similar heat map for rare cancers indicates that among these, kidney cancer,[60] thyroid cancer,[61] melanoma[62] and multiple myeloma,[63] a type of hematological malignancy, appears to a have number of patents filed by all six commercial companies. Examples of rare cancer that continue to be under explored commercially as exhibited by low patent publications include nasopharyngeal carcinoma,[64] blastic plasmacytoid dendritic cell cancer[65] and B-cell prolymphocytic leukemia[66, 67] among others (Figure S5).

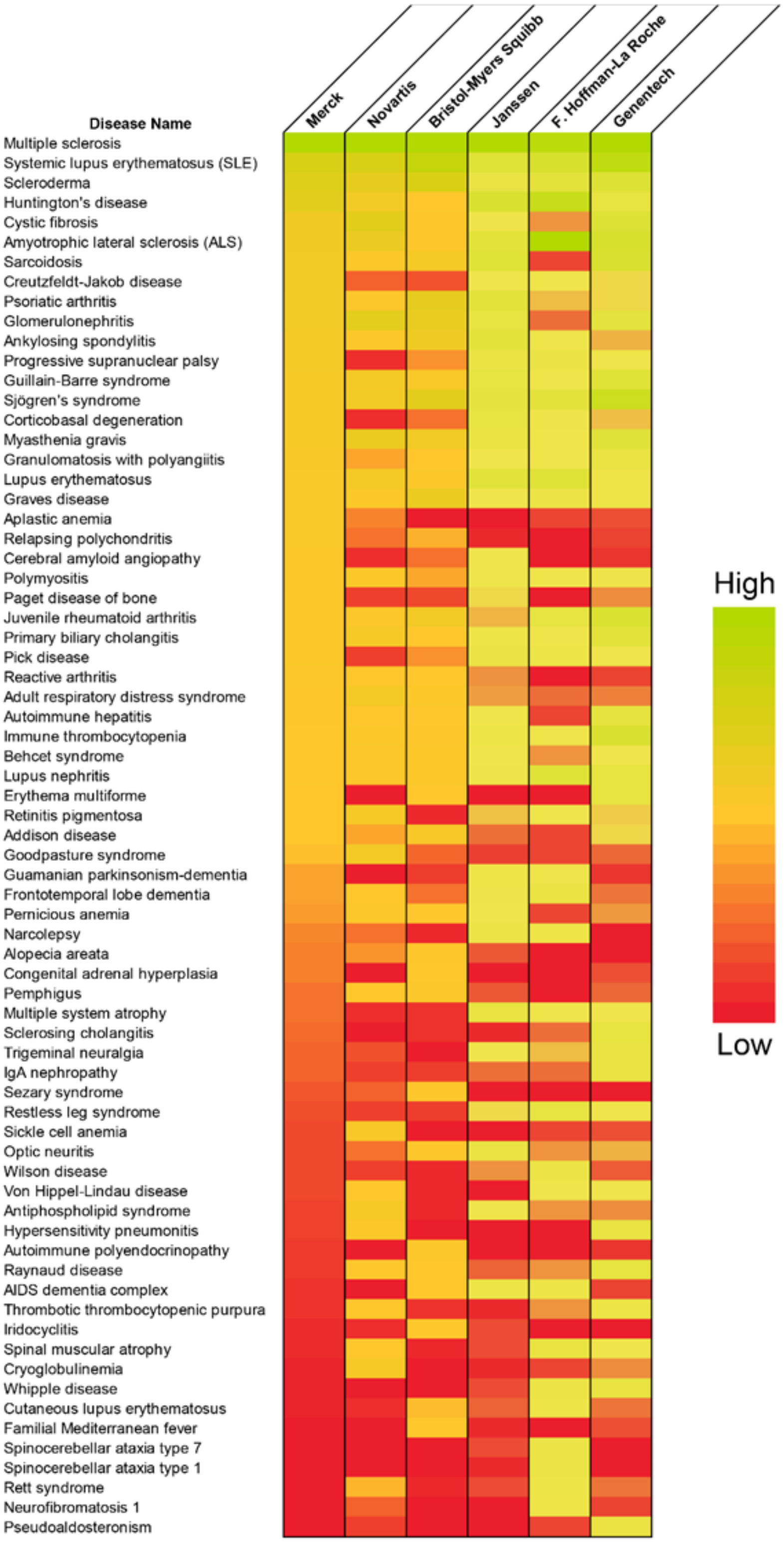

Figure S4. Heat map indicating number of patents filed by commercial organizations with respect to rare diseases. Patent assignees shown here are among the top 6 commercial patent assignees shown in Figure 4. Data includes patent publications in the field of rare diseases from the CAS Content Collection for 2003-2023.

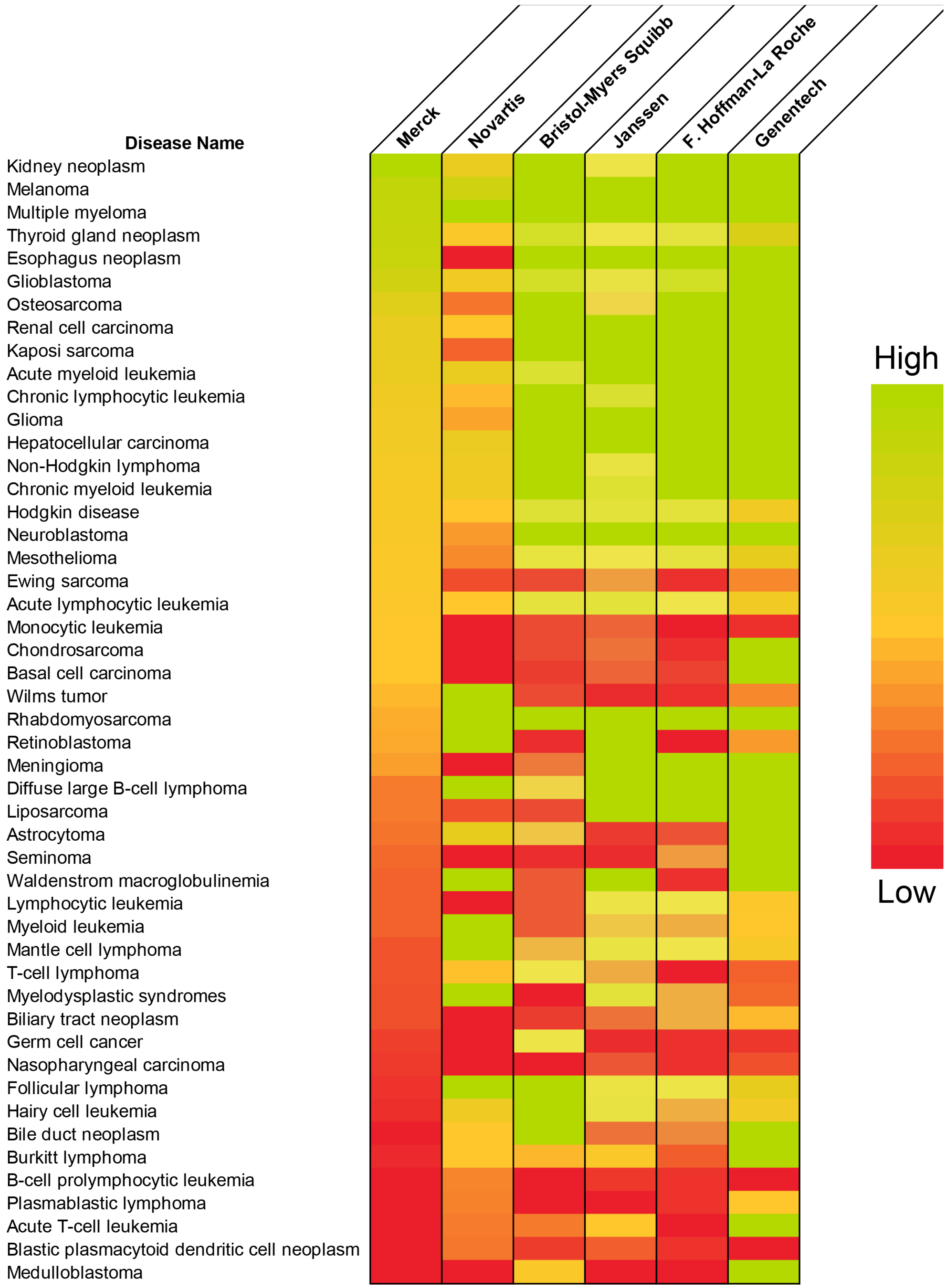

Figure S5. Heat map indicating number of patents filed by commercial organizations with respect to rare cancers. Patent assignees shown here are among the top 6 commercial patent assignees shown in Figure 4. Data includes patent publications in the field of rare diseases from the CAS Content Collection for 2003-2023.

(**A**)

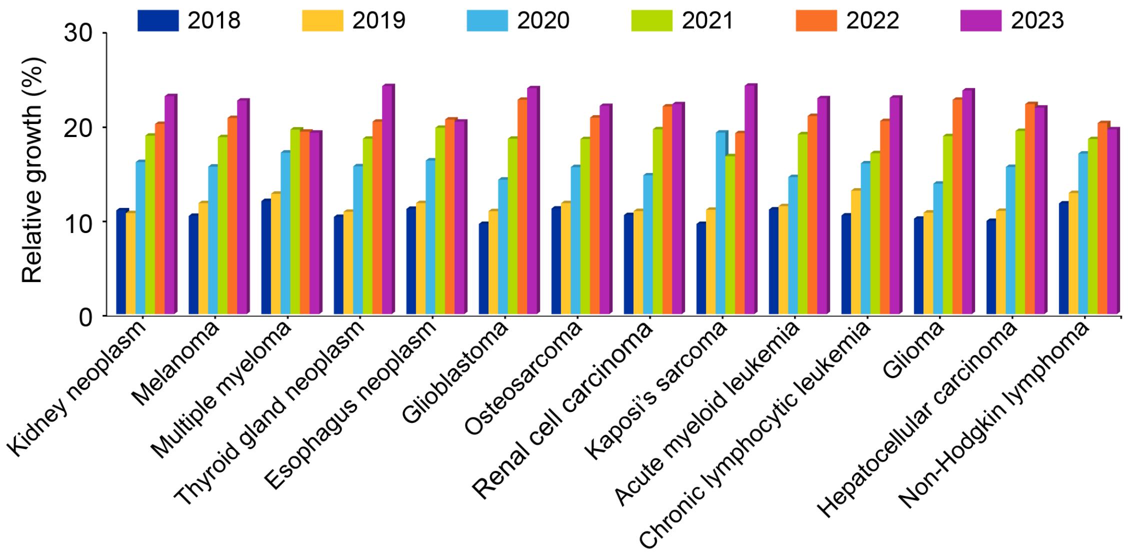

(**B**)

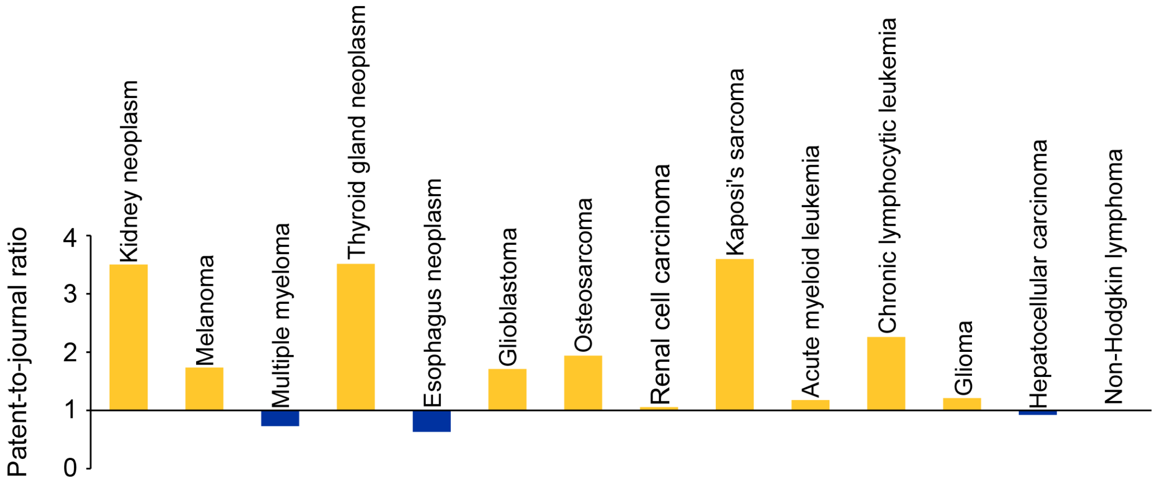

Figure S6. (**A**) Number of publications (journal and patent) and (**B**) patent-to-journal ratios for leading rare cancers in our dataset. Data includes both patent and journal publications sourced/extracted from the CAS Content Collection for the period 2018-2023 in the field of rare diseases.

Overview and pathogenesis of ALS

Overview

ALS leads to the gradual degeneration and death of motor neurons, which are the nerve cells responsible for transmitting signals from the brain to the muscles.[68, 69] As motor neurons deteriorate, voluntary muscle control and movement are progressively impaired, leading to muscle weakness, twitching (fasciculations), stiffness, and eventually paralysis. The symptoms of ALS can vary widely among individuals and may initially affect different muscle groups. Common early symptoms include weakness in the hands, arms, legs, or muscles involved in speech,

swallowing, or breathing. As the disease progresses, muscle weakness and atrophy spread to other parts of the body.[70] ALS can also affect the muscles involved in speech, swallowing, and breathing, leading to difficulties in speaking, chewing, swallowing (dysphagia), and breathing (respiratory compromise). Bulbar symptoms often contribute to significant disability and can pose life-threatening complications in advanced stages of the disease. In some cases, ALS may be associated with cognitive and behavioral changes, including difficulties with executive function, language, decision-making, and emotional regulation. This constellation of symptoms is referred to as frontotemporal dementia (FTD) or ALS-FTD, which represents a spectrum of overlapping neurodegenerative disorders.[68, 71-73]

ALS currently has no cure, and the underlying mechanisms driving the disease remain incompletely understood. Most cases of ALS are sporadic, meaning they occur without a clear family history, while a smaller percentage are inherited (familial ALS) due to genetic mutations. Several genes have been implicated in familial ALS, including C9orf72, SOD1, TARDBP, FUS, and others.[74-76]

Diagnosis of ALS is typically based on a thorough medical history, neurological examination, electromyography (EMG), nerve conduction studies, and exclusion of other possible causes of muscle weakness and motor dysfunction. Magnetic resonance imaging (MRI) and other imaging tests may also be used to rule out other conditions.[77, 78]

While there is no cure for ALS, various treatments and supportive interventions can help manage symptoms, improve quality of life, and prolong survival. These may include medications to alleviate muscle cramps, spasticity, or excessive saliva production, as well as physical therapy, occupational therapy, speech therapy, and assistive devices to maintain mobility and communication abilities.[79] In some cases, non-invasive ventilation or feeding tubes may be necessary to support respiratory and nutritional needs. The US Food and Drug Administration (FDA) has approved a few medications for treating symptoms related to ALS[80, 81] including the benzothiazole Riluzole (Rilutek, Exservan, Tiglutik)[82, 83] in 1995, the antioxidant Edaravone (Radicava)[84] in 2017, and Sodium phenylbutyrate-Taurursodiol (Relyvrio)[85, 86], a combination of a histone deacetylase inhibitor and a bile acid, in 2022. Sadly, Relyvrio has been just reported to have failed Phase III trial.[87] The Phase 3b trial of Radicava has been also discontinued in 2023, as well as its extension study.[88, 89] The prognosis for ALS varies, with most individuals experiencing progressive disability over time. The rate of disease progression and life expectancy can vary widely among individuals, ranging from a few years to more than a decade from the onset of symptoms. Respiratory failure is the most common cause of death in ALS.[90-92].

Pathogenesis

The pathogenesis of ALS is complex and involves a combination of genetic, environmental, and cellular factors. While the exact cause of ALS remains incompletely understood, several key mechanisms have been implicated in the degeneration of motor neurons characteristic of the disease.[73, 74, 93-96]

ALS primarily affects motor neurons, which are the nerve cells responsible for transmitting signals from the brain and spinal cord to the muscles, controlling voluntary muscle movement. The progressive degeneration and death of motor neurons lead to muscle weakness, atrophy, and eventual paralysis.[97, 98] Glutamate is the primary excitatory neurotransmitter in the CNS, including the motor neurons. In ALS, dysregulation of glutamate signaling occurs, leading to excessive levels of glutamate in the synaptic cleft. This excitotoxicity can damage motor neurons,

contributing to their degeneration and death.[99-101] Motor neurons are particularly vulnerable to oxidative stress, which occurs when there is an imbalance between the production of reactive oxygen species (ROS) and the body's ability to neutralize them with antioxidants. Oxidative stress can damage cellular structures, including proteins, lipids, and DNA, leading to motor neuron dysfunction and death.[102, 103] Abnormal protein aggregation is a hallmark feature of ALS pathology. Mutations in genes such as SOD1, TARDBP (encoding TDP-43), FUS, and C9orf72 can lead to the misfolding and aggregation of proteins within motor neurons and surrounding cells. These protein aggregates can disrupt cellular function and contribute to neurodegeneration.[104-107]

Mitochondria are the cellular organelles responsible for energy production and play a crucial role in maintaining neuronal function and viability. Dysfunction of mitochondria, including impaired energy metabolism and increased production of ROS, has been implicated in ALS pathogenesis, contributing to motor neuron degeneration.[108-110] In addition to motor neurons, non-neuronal cells such as astrocytes, microglia, and oligodendrocytes play important roles in ALS pathogenesis. Dysfunctional glial cells can contribute to neuroinflammation, excitotoxicity, and impaired neuronal support and repair mechanisms, further exacerbating motor neuron degeneration.[111-114] While most cases of ALS are sporadic, meaning they occur without a clear family history, approximately 5-10% of cases are familial and are associated with specific genetic mutations. Mutations in genes such as SOD1, TARDBP, FUS, and C9orf72 have been identified in familial ALS and can contribute to motor neuron dysfunction through various mechanisms.[115, 116] While the majority of ALS cases have no known cause, environmental factors such as exposure to toxins, heavy metals, pesticides, and traumatic brain injury have been suggested as potential risk factors for ALS. These factors may interact with genetic susceptibility to contribute to disease onset and progression.[117-119] Thus, ALS is likely a multifactorial disease involving interactions between genetic susceptibility, environmental factors, and various cellular mechanisms leading to motor neuron degeneration and progressive muscle weakness and paralysis. The high heterogeneity of ALS and the failures of the efforts to find a singular cure have led some researchers to propose that it may not be a single disease, but rather a miscellany of overlapping conditions that share common characteristics, similarly to cancer. [120, 121] Further research is needed to fully elucidate the complex pathogenesis of ALS and identify effective therapeutic strategies for the treatment of this devastating neurodegenerative disorder.

Overview and pathogenesis of Huntington's disease

Overview

HD is caused by a mutation in the HTT gene, located on chromosome 4, which encodes the huntingtin protein. This mutation involves an expansion of a trinucleotide repeat sequence, known as CAG, within the gene. Individuals with HD inherit an expanded CAG repeat from one of their parents, leading to the production of a mutant huntingtin protein. The length of the CAG repeat is inversely correlated with the age of onset and severity of symptoms, with longer repeats generally associated with earlier onset and more severe disease. [122-125]

HD follows an autosomal dominant pattern of inheritance, meaning that a person who inherits a single copy of the mutant gene from one parent will develop the disease, regardless of whether the other parent carries the mutation. Each child of an affected individual has a 50% chance of inheriting the mutated gene.[126, 127] The mutant huntingtin protein disrupts normal cellular functions and leads to neuronal dysfunction and death, particularly in the basal ganglia and

cerebral cortex of the brain. These regions are involved in movement control, cognition, and emotion regulation. Progressive degeneration of these brain areas results in the characteristic symptoms of HD, which typically worsen over time.[122, 128, 129]

The most prominent early symptoms of HD are often motor-related and may include involuntary movements called chorea, which are rapid, jerky, and random. Other motor symptoms can include dystonia (sustained muscle contractions causing twisting or repetitive movements), rigidity, bradykinesia (slowness of movement), and impaired coordination and balance.[130-132] HD also affects cognitive function, leading to impairments in memory, executive function, attention, and decision-making. As the disease progresses, individuals may experience difficulties with language, planning, problem-solving, and other cognitive tasks. [133, 134] Psychiatric symptoms are common in HD and can precede motor symptoms by several years. These can include depression, anxiety, irritability, apathy, impulsivity, and obsessive-compulsive behaviors. Psychiatric symptoms can significantly impact quality of life for individuals with HD and their caregivers.[135, 136] While most cases of HD typically manifest in adulthood, a small percentage of individuals develop symptoms during childhood or adolescence, known as juvenile-onset HD. Juvenile-onset HD tends to progress more rapidly and may have distinct clinical features compared to adult-onset HD.[130, 137, 138]

Currently, there is no cure for HD, and available treatments focus on managing symptoms and improving quality of life. Medications can help alleviate motor symptoms, psychiatric symptoms, and complications such as chorea and depression. Doctors utilize an arsenal of medications to help alleviate the symptoms of Huntington's disease including antipsychotics and antidepressants. Tetrabenazine (Xenazine)[139] and deutetrabenazine (Austedo)[140, 141] are two US FDA drugs approved in 2008 and 2017, respectively, for treating chorea in Huntington's disease. Physical therapy, speech therapy, and occupational therapy may also be beneficial in managing motor and functional impairments.[124, 142, 143] Genetic testing can confirm the diagnosis of HD in individuals with symptoms or a family history of the disease. Genetic counseling is recommended for individuals considering testing to discuss the implications of the test results and the potential impact on themselves and their families.[130, 144] Ongoing research efforts aim to better understand the underlying mechanisms of HD and develop disease-modifying treatments. Clinical trials are underway to test potential therapies targeting the mutant huntingtin protein, neuroinflammation, and other pathways implicated in HD pathogenesis.[145-147] Despite the challenges posed by HD, advances in research and clinical care offer hope for improved treatments and ultimately a cure for this devastating neurodegenerative disorder.

Pathogenesis

The pathogenesis of Huntington's disease (HD) involves a complex interplay of genetic, molecular, and cellular mechanisms that ultimately lead to neurodegeneration in specific regions of the brain. [125, 148-150]

HD is caused by a mutation in the HTT gene, located on chromosome 4. This mutation involves an abnormal expansion of a trinucleotide repeat sequence, known as CAG, within the gene. The CAG repeat encodes a polyglutamine tract in the huntingtin protein. The mutant huntingtin protein (mHTT) has an expanded polyglutamine tract, which is toxic to neurons and disrupts normal cellular functions.[122, 130, 151] The expanded polyglutamine tract in mHTT leads to the misfolding and aggregation of the protein. These aggregates, known as inclusion bodies,

accumulate within neurons and disrupt cellular processes. Protein aggregation is a hallmark feature of HD pathology and contributes to neuronal dysfunction and death.[152-154]

mHTT disrupts mitochondrial function, leading to impaired energy production, increased oxidative stress, and mitochondrial fragmentation. Mitochondrial dysfunction contributes to neuronal vulnerability and exacerbates neurodegeneration in HD.[155-157] Dysregulation of glutamate signaling and excitotoxicity play a role in HD pathogenesis. mHTT disrupts glutamate homeostasis, leading to excessive glutamate release and activation of NMDA receptors. Prolonged activation of NMDA receptors results in calcium influx, mitochondrial dysfunction, and neuronal damage.[158-161]

mHTT interferes with axonal transport, the process by which cellular components are transported along axons. Impaired axonal transport disrupts the delivery of essential proteins and organelles to synapses, leading to synaptic dysfunction and neuronal degeneration. [162-164] Neuroinflammatory processes contribute to HD pathogenesis and exacerbate neuronal damage. Activation of microglia and astrocytes, the resident immune cells of the CNS, leads to the release of pro-inflammatory cytokines, ROS, and other toxic molecules that contribute to neuronal dysfunction and death. [165-167]

mHTT disrupts gene transcription and expression, leading to widespread alterations in gene expression patterns in affected neurons. Transcriptional dysregulation affects multiple cellular pathways involved in neuronal function, survival, and plasticity, contributing to neurodegeneration in HD.[168-170] HD pathology affects synaptic function and plasticity, leading to impaired neurotransmission and synaptic loss. Synaptic dysfunction contributes to cognitive and motor impairments in HD and is an early feature of the disease.[171, 172] Certain neuronal populations, particularly those in the striatum and cerebral cortex, are selectively vulnerable to the toxic effects of mHTT. The striatum, which plays a crucial role in motor control and cognition, is severely affected in HD, leading to the characteristic motor and cognitive symptoms of the disease.[173-175]

Overview and pathogenesis of myasthenia gravis

Overview

The hallmark symptom of MG is muscle weakness, which typically worsens with activity and improves with rest. This weakness can affect various muscles, including those controlling eye movements, facial expressions, chewing, swallowing, and breathing. Fatigue is also a common feature, with muscles becoming progressively weaker during periods of activity. Weakness in the muscles that control eye movements often leads to double vision or drooping of the eyelids (ptosis). Difficulty swallowing (dysphagia) can occur due to weakness in the muscles involved in chewing and swallowing. In severe cases, weakness of the muscles involved in breathing can lead to respiratory difficulties, which can be life-threatening. Symptoms of MG can vary widely among individuals and may fluctuate over time, making diagnosis challenging. [176-180]

Factors such as stress, illness, fatigue, or certain medications can exacerbate symptoms in people with MG. Diagnosis typically involves a thorough medical history, physical examination, blood tests to check for specific antibodies associated with MG, and specialized tests such as electromyography and nerve conduction studies. While there is no cure for MG, various treatments can help manage symptoms and improve quality of life. These may include medications such as acetylcholinesterase (AChE) inhibitors, immunosuppressants, and

corticosteroids.[181-183] Some individuals may also benefit from procedures such as plasmapheresis or intravenous immunoglobulin therapy. In severe cases, surgical removal of the thymus gland (thymectomy) may be recommended.[184] With appropriate treatment, many people with MG can lead fulfilling lives. However, the course of the disease can be unpredictable, and long-term management often requires close monitoring and adjustments to treatment by developing a personalized treatment plan and receive ongoing support and care.[185-191]

Pathogenesis

The pathogenesis of MG involves an autoimmune response targeting components of the neuromuscular junction (NMJ), where nerve impulses trigger muscle contractions.[192, 193] MG is primarily driven by an autoimmune response, where the body's immune system mistakenly identifies components of the NMJ as foreign and attacks them. The primary target of this autoimmune response is the AChRs on the muscle cell membrane. These receptors normally bind acetylcholine, a neurotransmitter released by motor neurons, to initiate muscle contractions.[194-196]

In MG, the immune system produces autoantibodies called anti-AChR antibodies. These antibodies bind to the AChR on the muscle cell membrane, leading to several effects: (i) some antibodies block the binding sites on the AChR, preventing acetylcholine from binding and initiating muscle contractions; (ii) other antibodies may cross-link adjacent AChR molecules, leading to internalization and degradation of the AChR complex, reducing the number of functional receptors on the muscle cell membrane; (iii) the binding of antibodies to AChR can also activate the complement system, a part of the immune system involved in inflammation and cell destruction. This further contributes to damage and dysfunction at the NMJ.[197-200]

The presence of anti-AChR antibodies and complement activation disrupts neuromuscular transmission, leading to: (i) reduced signal transmission, since with fewer functional AChR available, the binding of acetylcholine released by motor neurons is impaired, resulting in weakened muscle contractions; (ii) endplate destruction, since chronic immune-mediated damage to the NMJ can lead to structural changes, including destruction of the postsynaptic membrane and alterations in the distribution of AChR.[201-203]

The thymus gland plays a role in the development of MG in some individuals.[204] It is commonly associated with thymic abnormalities, such as thymic hyperplasia or thymoma (a tumor of the thymus). The thymus is thought to contribute to the production of autoantibodies and the maturation of autoreactive T cells involved in the autoimmune response seen in MG. In general, the pathogenesis of MG involves a complex interplay between autoantibodies, complement activation, and immune-mediated damage at the neuromuscular junction, leading to impaired neuromuscular transmission and muscle weakness characteristic of the disease.[204-206]

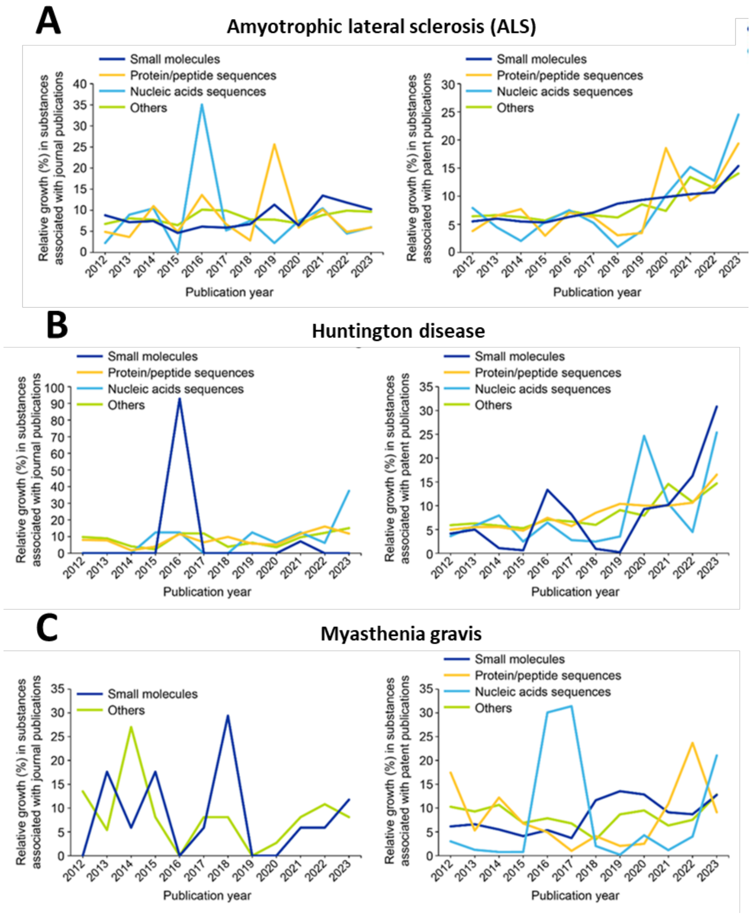

Figure S7. Annual growth of the individual substance classes associated with (**A**) amyotrophic lateral sclerosis (ALS), (**B**) Huntington's disease and (**C**) myasthenia gravis. Data includes substances associated with both patent and journal publications sourced from the CAS Registry and the CAS Content Collection for the period 2012-2023.

ALS

Protein/Peptide sequences	Number of publications (2013-2023)
Cyclosporin	123
Actinomycin D	73
Amylin	70
Alemtuzumab	67
Bevacizumab	64
Natalizumab	62
Adalimumab	61
Trastuzumab	57
Etanercept	52

Huntington's disease

Protein/Peptide sequences	Number of publications (2013-2023)
Cyclosporin	74
Amylin	53
Bevacizumab	47
Natalizumab	43
Alemtuzumab	43
Actinomycin D	37
Trastuzumab	36
Adalimumab	33
Exenatide	31

Myasthenia gravis

Protein/Peptide sequences	Number of publications (2013-2023)
Cyclosporin A	34
Actinomycin D	27
Adalimumab	26
Abatacept	25
Certolizumab	24
Tocilizumab	23
Alemtuzumab	23
Obinutuzumab	22
Natalizumab	22

Figure S8. Leading substances from the CAS Registry associated with amyotrophic lateral sclerosis (ALS), Huntington's disease (HD) and myasthenia gravis (MG) for the protein/peptide sequences subclass of substances. Data includes substances associated with both patent and journal publications sourced from the CAS Registry and the CAS Content Collection for the period 2012-2023.

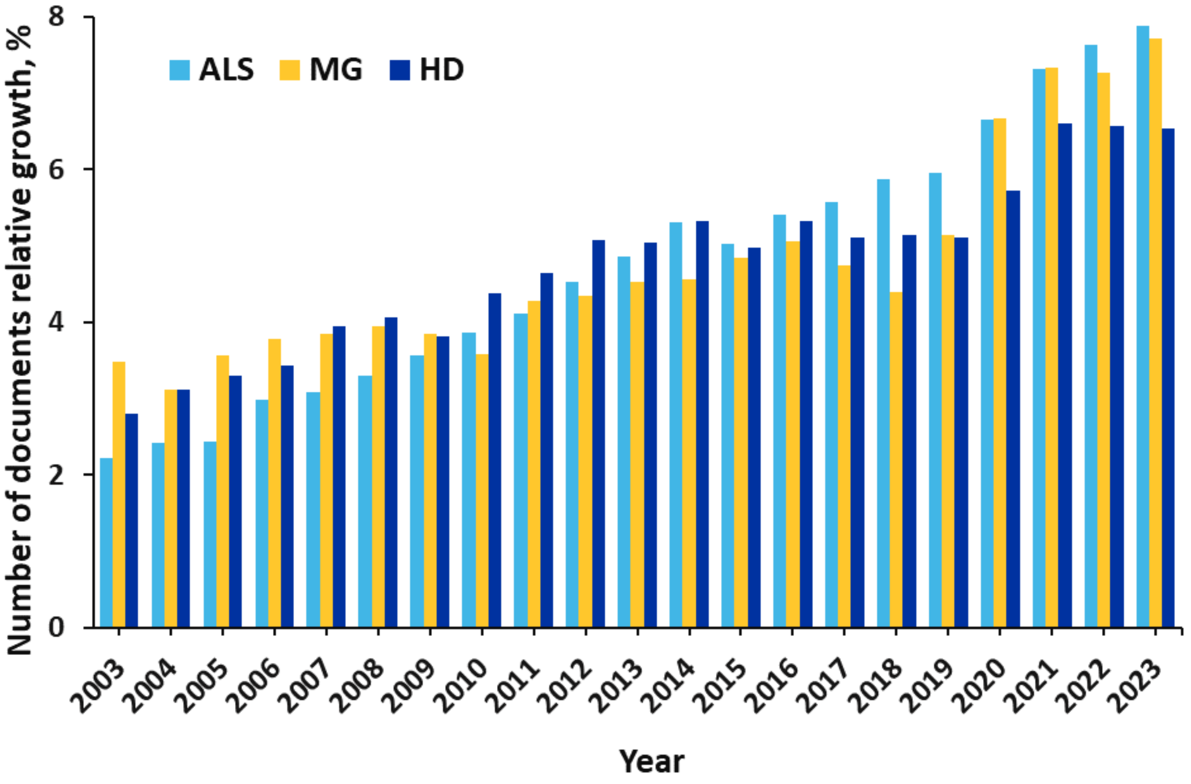

Figure S9. Relative growth in the number of documents related to ALS, MG, and HD in 2003-2023.

Capital investment

Capital investment data from Pitchbook (*Data has not been reviewed by PitchBook analysts) – an online platform for investment data, reveals a more or less consistent level of capital that has been invested in the fields of ALS, HD, and MG over the past 10 years (Figure S10) while the number of deals shows a moderate increase during the same period. Interestingly, there is a mild decline in the amount of money invested in this field over the last 3 years (2021-2023) which could be indicative of a slight decrease in commercial interest but the exact reason for this remains unknown. In terms of the geographical distribution of capital investment in the field of rare diseases, the US led with respect to capital investment from 2013 to 2023, followed by Belgium, the Netherlands, and the UK (Figure S10B). The investment in the US is ~14 times that of the next top contributor - Belgium and ~27 times that of the Netherlands. Growth in the capital investment made from 2013-2023 for the few leading countries/regions shows a moderate overall increase till 2021, post which it shows a minor dip till 2023 (Figure S10). Of note, the capital investment by Belgium and the UK has increased in the last 2 years (2021-2023).

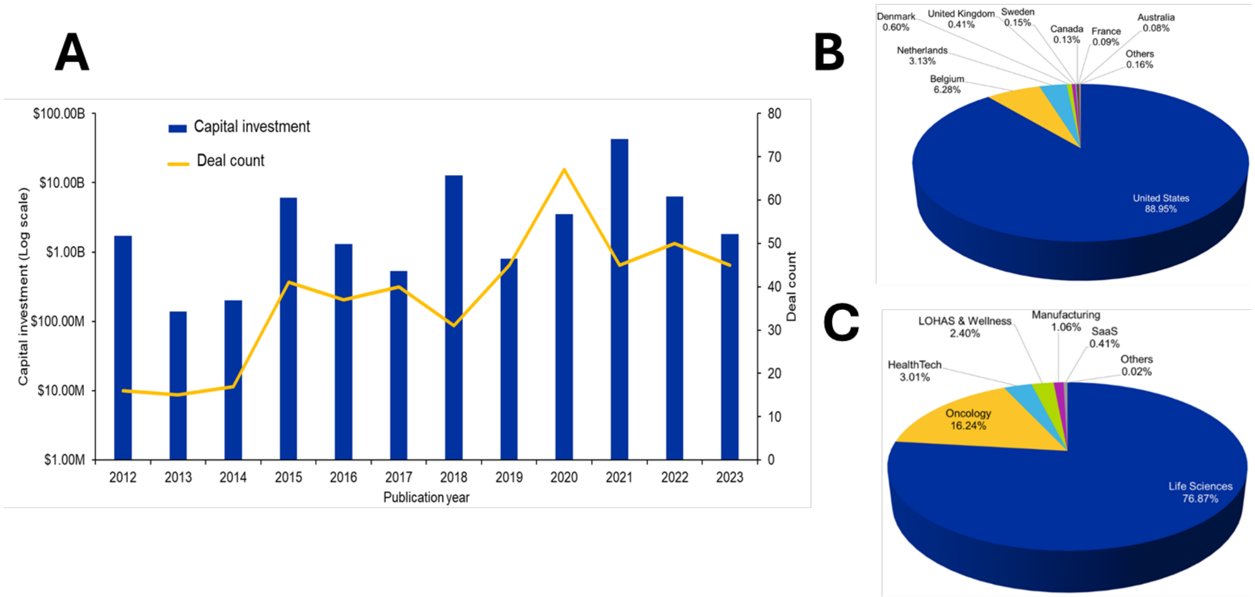

Figure S10. (**A**) Commercial interest in rare diseases (data sourced from PitchBook; *Data has not been reviewed by PitchBook analysts). Capital invested and deals related to rare diseases (ALS, HD, and MG) for the past decade (2012−2023); (**B**) Geographical distribution of the number of companies engaged in the field of rare diseases (ALS, HD, and MG); (**C**) Sector-wise distribution of various industry types investing in the field of rare diseases (ALS, HD, and MG).

A closer look at the data suggests that life sciences-based industries are the top investors followed by oncology-based and health technology industries (Figure 11B). Vaccinex has the maximum number of deals in this field. Vaccinex lead drug candidate, pepinemab (CAS RN: 2097151-87-4), blocks semaphorin 4D (SEMA4D), a key driver of neuroinflammation. This drug has the potential as a disease-modifying treatment for HD, Alzheimer's, and other neurodegenerative diseases and is currently in Phase II clinical trials. [207, 208] Similarly, Cytokinetics is investing in diseases linked to neuromuscular junction such as ALS. [209] Sangamo Therapeutics is working towards the preclinical development of a zinc finger transcriptional repressor targeting the SCN9A gene as a novel therapy for peripheral neuropathic pain which could be effective against ALS. [210] These investment trends indicate the steady interest of companies in the field of rare diseases such as ALS, HD, and MG.

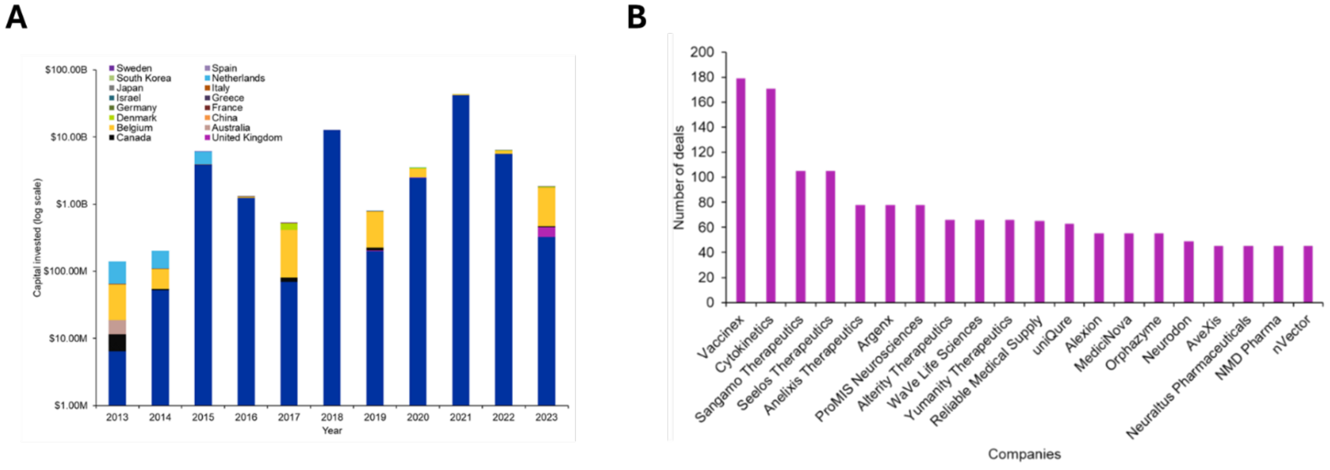

Figure S11. (A) Growth in the number of deals in the field of rare diseases by different countries/regions from 2013 to 2022;) **(B)** Growth in the number of deals in the field of rare diseases by different companies from 2013 to 2022.

Notable patents on ALS, Huntington's disease, and myasthenia gravis

Table S5 shows notable patents in the field of rare diseases such as ALS, HD and MG published in recent years (2020 to 2023). Patents were selected based on relevance, novelty, applicability, and field of study. Most of these involve therapeutic strategies, disease markers and recent advancements in these disease areas.

For instance, a recently published patent CN117050134 describes the synthesis and characterization of a novel oleanamide derivative for activating KEAP/NRF2/ARE signaling pathway which is a signature of oxidative stress. This treatment activates NRF2 transcription factor that can help in preventing and treating various neurological disorders including ALS. In context of HD, WO2023099648 by AstraZeneca AB, Sweden, describes pyrazolo- and triazolo-azinone compounds that inhibits receptor-interacting protein kinase 1 (RIPK1) and can be used in the treatment of neurological disorders including HD. In another example, patent application WO2023236967A1 by RemeGen Co., Ltd., China, describes the development of the drug, a dosage regimen, an administration interval, and a mode for treating MG using - Telitacicept (TACI-Fc fusion protein). It is shown that this formulation exhibits good clinical efficacy and safety in the treatment of MG patients. These patents higlight the constant research endevours in the feild of rare diseases

Table S1. List of genes associated with amyotrophic lateral sclerosis (ALS), as shown in Figure 3A, with the association type as causal or contributing.

Gene	Association score	Protein	Expression profile, function and other diseases	Amyotrophic lateral sclerosis (ALS)
SOD1[211]	1.0	Superoxide dismutase 1	An enzyme that scavenges superoxide radicals, superoxide dismutase 1 is crucial in converting ROS to molecular oxygen (O_2) and hydrogen peroxide (H_2O_2).[212, 213] Also referred to as copper-zinc superoxide dismutase (Cu/Zn SOD1, CuZn SOD1) due to the presence of a copper and Zn ion in the enzyme. [213] Superoxide dismutase are expressed in the neurons of CNS and are linked to neurological disorders.[214]	Mutations in superoxide dismutase 1 are thought to result in protein misfolding leading to ALS.[215, 216] In silico studies have tried to decipher changes in structure of mutant superoxide dismutase 1 resulting in "pathogenic conformational states" that might promote aggregation.[217] Researchers have also found a disulfide bond (C57-C146) to be critical for the normal functionality of superoxide dismutase 1.[218, 219] Research continues to be conducted to understand the exact effect of a mutation on protein structure using molecular modeling and simulation studies.[220]
SETX[221]	1.0	Senataxin	An enzyme classified as a superfamily 1B helicase, senataxin plays a critical role in resolving R-loops acting as a RNA:DNA helicase during DNA damage repair.[222] Mutations in SETX have been found in ataxia with oculomotor apraxia (AOA2). [223-226]	Mutations in senataxin have been linked to ALS4 (juvenile amyotrophic lateral sclerosis-4 with onset occurring before the age of 25[227]).[228, 229] These mutations are thought to interfere with senataxin's ability to regulate RNA polymerase II, control R loops, and binding to other protein partners among other things.[230, 231]
TARDBP[232]	0.9	Transactive response (TAR) DNA-binding Protein 43 kDa (TDP-43)	Protein composed of ~400 amino acid belonging to the heterogeneous nuclear ribonucleoprotein family.[233] TDP-43 is largely composed of 4 domains[234] and plays a role in RNA regulation.[233] Primarily a nuclear protein, TDP-43 shuttles between the nucleus and cytoplasm.[235] TDP-43 has been implicated in neurological disorders such as Alzheimer's,[236-239] Parkinson's [240] disease,	Mutations in TDP-43 have been observed in ALS.[244-247] These mutations can lead to formation of amyloid-like fibrils or cause TDP-43 to localize in the cytoplasm instead of the nucleus affecting RNA regulation.[235, 248] Recently researchers have determined that the aggregated forms of TDP-43 exhibit distinct folds depending on the type of disease (ALS [249] vs. type B FTLD-TDP

Gene	Association score	Protein	Expression profile, function and other diseases	Amyotrophic lateral sclerosis (ALS)
			as well as frontotemporal dementia (FTD).[241-243]	[250]). A functional dimer, monomerization of TDP-43 has been shown to be important in ALS.[251]
OPTN[252]	0.9	Optineurin	A ~570 amino acid cytosolic protein that is expressed in a variety of tissues including the brain, heart, skeletal muscle, and eye among others.[253] Optineurin is involved in vesicular trafficking[254] and immune signaling.[255] Optineurin aggregates have been reported in Parkinson's disease[256] and its expression altered in a Parkinson's disease model.[257] Optineurin has a possible role in promoting aggregation of mutant huntingtin.[258] On the other hand, optineurin might have a beneficial role in Alzheimer's disease.[259]	Mutations in optineurin have been reported in ALS[260-265] with mutations affecting optineurin's ability to interact with mitochondria and affecting mitophagy.[253, 266] OPTN gene therapy is being explored as possible treatment option for ALS.[267]
SQSTM1[268]	0.9	Sequestosome 1	Also referred to as p62, sequestosome 1 is a selective autophagy receptor.[269] Composed of multiple protein-protein interaction motifs, sequestosome 1 functions to eliminate misfolded and aggregated protein.[270] In addition, sequestosome 1 is important in aging and age-related diseases.[269] Besides ALS, SQSTM1 has been implicated in FTD[271-274] and Alzheimer's disease[275] among other neurological disorders.	Similar to the other genes listed in this table, mutations in sequestosome 1 have been identified in individuals suffering from ALS.[276-279] For some mutations in sequestosome 1 its ability to bind to other protein binding partners is impaired leading to reduced autophagy.[280] Therefore, compounds enhancing autophagy may be a potential treatment strategy.[281]
ANG[282]	0.9	Angiogenin	As the name suggests, angiogenin is a small (~120 amino acid residues) protein that is crucial in angiogenesis (the formation of new blood vessels).[283, 284] Besides angiogenesis, other processes involving angiogenin include rRNA and mRNA transcription, tRNA metabolism[285] as well as playing a role in immune response.[286] Angiogenin belongs to the ribonuclease A superfamily.	Mutations in ANG, often loss-of-function, have been reported in ALS.[287, 288] Research is ongoing to determine the exact effect of reported/known ALS mutations on the structure and ribonuclease A activity of angiogenin.[289, 290] In a recent report, the role of angiogenin in ALS was shown to be more complex than was earlier thought.[291]
VAPB[292]	0.9	Vesicle-associated membrane protein	VAPB is a type IV membrane protein consisting of ~243 amino acids associated	Mutations in VAPB have been detected in ALS patients.[298, 299] One mutation in

Gene	Association score	Protein	Expression profile, function and other diseases	Amyotrophic lateral sclerosis (ALS)
		(VAMP)-associated protein B and C (VAPB)	with the endoplasmic reticulum.[293] Known to interact with a wide variety of partners[294] often (but not always) via interaction between its MSP domain and a FFAT motif of the binding partner.[295] VAPB has been implicated in a number of neurological disorders including Alzheimer's and Parkinson's disease.[294] In addition, the role of VAPB in cancer is being actively studied.[296, 297]	particular appears to have been studied extensively in the context of ALS – a Pro-to-Ser (P56S) mutation.[300-307] The P56S mutation appears to a toxic gain-of-function mutation and number of mechanisms have been proposed including affecting autophagy,[307] causing endoplasmic reticulum stress,[304, 306] changing the morphology of endoplasmic reticulum,[308] and diminishing VAPB's ability to mediate unfolded protein response.[300] Other mutations perhaps less well studied include P56H,[309] T46I,[310] V234I,[311] and a deletion mutant (ΔS160).[312]
PRPH[313]	0.9	Peripherin	A type III intermediate filament protein, which are part of the cytoskeleton (other members include desmin, vimentin and glial fibrillary acidic protein).[314] Unlike other type III intermediate filament proteins, peripherin is expressed primarily in the peripheral and CNS.[314, 315] Peripherin is thought to play a role in axonal growth.[316, 317] Researchers are exploring peripherin as potential biomarker for motor neuron diseases[318] and axonal damage.[319]	Upregulation of peripherin has been reported in ALS[320] with this upregulation proposed to have a detrimental effect as observed in mouse models.[321] Others have reported mutations in peripherin in ALS patients.[322] Peripherin splice variants prone to aggregation have also been reported.[320, 321]
CHCHD10[323]	0.9	Coiled-coil-helix-coiled-coil-helix domain containing protein 10	A small protein (~149 amino acid residues) associated with the intermembrane space of mitochondria,[324] coiled-coil-helix-coiled-coil-helix domain containing protein 10 is also known as protein N27C7-4.[325] Like other members of the CHCHD-containing protein family, CHCH10 protein contain $(CX_9C)_2$ motifs.[324] Appears to play a role in mitochondrial structure and function.[324] Other diseases associated with CHCHD10 include late onset spinal motor	Mutations in CHCHD10 have been reported in ALS patients.[328-331] Overexpression of a mutant CHCHD10 was associated with abnormalities in mitochondria[329] and could underlie pathogenesis of ALS and FTD.

Gene	Association score	Protein	Expression profile, function and other diseases	Amyotrophic lateral sclerosis (ALS)
			neuronopathy[326] and mitochondrial myopathy.[327]	
TBK1[332]	0.9	TANK binding kinase 1	A serine/threonine protein kinase composed of ~729 amino acid residues, TBK1 plays a role in innate immunity, autophagy and apoptosis among other cellular functions.[333] TBK1 binds to and interacts with a wide variety of protein partners utilizing different domains present in its structure.[333-335] Activation of TBK1 requires autophosphorylation at Ser172.[334, 336] Diseases that involve TBK1 include cancer[333, 337, 338] and autoimmune disorders[339, 340] with TBK1 inhibitors being explored as a viable strategy.[339, 341, 342]	Among the many binding partners of TBK1 are optineurin and sequestosome 1/p62. Mutations in TBK1 have been reported in ALS[343-345] with mutations thought to interfere with autophagic function of TBK1[346] by affecting kinase activity.[347] Besides ALS, TBK1 mutations are also implicated in FTD.[348, 349]
NEFH[350]	0.9	Neurofilament heavy chain	Neurofilaments are heavily expressed in the central and peripheral nervous system.[351] They are made up of three subunits based on molecular weight, light, medium and heavy (NEFH).[352] They are also involved with cytoskeletal functions such as the regulation of axon diameter and growth.[353] Besides ALS, the other disease associated with NEFH includes Charcot-Marie-Tooth disease.[354-356]	Mutations in NEFH proteins have been reported to be associated with ALS such as those within the KSP repeat region of the tail domain.[357-359] Another study reports variants in NEFH associated with sALS such as the NEFH Ser787Arg.[360]
FUS[361]	0.9	Fused in sarcoma (FUS) RNA binding protein	A DNA/RNA binding protein, FUS is composed of ~506 amino acids.[362] FUS is expressed both in the nucleus and cytoplasm [362] and participates in a number of important cellular processes such as DNA repair[363] including mitochondrial DNA repair[364] and mRNA transport,t[364] among others. Some other names that FUS is known by include translocated in liposarcoma (TLS) and heterogeneous nuclear ribonucleoprotein P2 (HNRNPP2). Besides ALS, FUS has also been linked to FTD.[365, 366]	Mutations in the FUS gene have been linked to ALS over the last two decades.[367-374] Some ALS-associated mutations have been found in the nuclear localization signal domain dampening the ability of FUS to bind transportin 1, a nuclear import receptor,[375] resulting in accumulation of mutant FUS in the cytoplasm.[376] An article published in 2018 describes FUS R495X ALS-associated mutation resulting in alterations in the mitochondria.[377]

Gene	Association score	Protein	Expression profile, function and other diseases	Amyotrophic lateral sclerosis (ALS)
PFN1[378]	0.9	Profilin 1	Profilin 1 is a small actin binding protein composed of ~139 amino acid residues with a wide expression profile.[379] Profilin 1 helps in actin polymerization[380] by accelerating nucleotide exchange or by lowering actin concentration required to jump start polymerization.[379] Besides actin polymerization, profilin 1 appears to be important for a number of cellular processes including cell division[381] and membrane trafficking.[382, 383] In the last decade, profilin 1's role in cancer has investigated[384] with dysregulation of profilin 1 observed in breast cancer,[385] endometrial cancer,[386] as well as non-small cell lung cancer,[387] among others.	Mutations in profilin have been observed in ALS[388-390] – aggregation of mutant profilin 1 often alongside TDP-43 is thought to be a contributing factor to ALS. Mutation in the phosphorylation site of profilin 1, important for actin polymerization, has also been reported.[391] Molecular modeling and dynamic studies indicate that some mutations in profilin1 might affect the stability and functionality of the protein.[392]
MATR3[393]	0.9	Matrin 3	A DNA/RNA binding nuclear protein, matrin 3 is composed of ~847 amino acid residues [394] organized into several domains such as RNA recognition motifs, zinc finger domain, and intrinsically disordered regions.[395, 396] The various functions that matrin 3 is involved in includes mRNA stabilization,[397] alternative splicing regulation,[398] DNA repair,[399] etc.[396]	Matrin 3 binds to and interacts with a number of different proteins including TDP-43 and FUS, which are also implicated in ALS.[396] Mutations in MATR3 has been detected in ALS patients.[400-404]
UBQLN2[405]	0.9	Ubiquilin 2	A ubiquitin-like protein, ubiquilin 2 is an important component of the ubiquitin-proteosome system helping in the degradation of misfolded proteins.[406] The ubiquitin-like domain and ubiquitin-associated domains of ubiquilin 2 interact with the proteosome and the polyubiquitin chains on proteins, respectively. [407]	Mutations in UBQLN2 linked to ALS[408-410] possibly by hampering protein degradation by affecting its interaction with binding partners/other proteins.[411, 412]
ALS2[413]	0.8	Alsin	Composed of ~1657 amino acids arranged into four domains, alsin is a large protein that acts as a guanine nucleotide exchange factor for Rac1 and Rac5[414] and plays a role in membrane trafficking.[414, 415] Alsin expression has been observed in the CNS in mice[416] and studies with alsin knockdown	Mutations in alsin have been detected in ALS patients.[418-421] Some ALS-associated mutations result in expression of short and long form of alsin lacking crucial domains, diminishing alsin's ability to effectively function as a guanine nucleotide exchange factor.[418]

Gene	Association score	Protein	Expression profile, function and other diseases	Amyotrophic lateral sclerosis (ALS)
			and Rac1 mutants indicate that alsin-Rac1 signaling is important for axonal growth in motor neurons.[417]	
FIG4[422]	0.8	Polyphosphoinositide phosphatase	Also known as SAC domain-containing protein 3 (Sac3) and is composed of ~907 amino acid residues.[423, 424] An enzyme, polyphosphoinositide phosphatase cleaves the phosphatase group from the 5-position of the inositol ring of phosphatidylinositol 3,5-bisphosphate (PI(3,5)P_2).[424, 425] PI(3,5)P_2 is a lipid signaling molecule and thought to be important for development of the nervous system.[426] Mutation in FIG4 and deficiency of FIG4 are associated with Charcot-Marie-Tooth disease,[427] an inherited genetic condition affecting peripheral nerves,[428] and lysosomal storage disorder.[429]	FIG4 mutations have been observed in ALS patients.[430-436] While the exact mechanism underlying ALS-associated disease mutations remains under investigation, evidence from FIG4's involvement in Charcot-Marie-Tooth disease indicates disruption of intracellular trafficking due to excessive vacuoles could be a possible reason.[437] [437]
KIF5A[438]	0.8	Kinesin family member 5A	Kinesin family member 5A belongs to the kinesin family of ATP-driven motor proteins.[439] Also referred to as kinesin-1, they are expressed in neurons[440] and are engaged in intracellular transport of protein complexes, mRNAs and others[441] as well as cell division.[442] Composed of ~1039 amino acid residues, kinesin family member 5A appears to be involved in trafficking of GABA receptors.[443] Under physiological conditions, kinesin family member 5A are autoinhibited [444] rendering them incapable of interacting with cargo and only upon binding of two partners – c-Jun N-terminal kinase–interacting protein 1 (JIP1) and fasciculation and elongation protein ζ1 (FEZ1) – is the autoinhibition lifted allowing kinesin family member 5A to carry out transport.[445] Besides ALS, expression levels of KIF5A appear to be altered in Alzheimer's disease[446, 447] and	In the last few years, there have been reports of KIF5A mutations observed in ALS patients.[403, 451-456] Recently, Baron et al.[457] elucidated the mechanism underlying an ALS-associated mutations in KIF5A – as per their explanation, the mutation rendered the autoinhibition of kinesin family member 5A ineffective resulting in increased mitochondrial transport. A research group from Tohoku university showed that an ALS-associated mutant of KIF5A had increased propensity to form aggregates.[458] Other ALS-associated mutations in KIF5A have also been identified.[459]

Gene	Association score	Protein	Expression profile, function and other diseases	Amyotrophic lateral sclerosis (ALS)
			mutations in KIF5A appear to be associated with spastic paraplegia.[448-450]	
ERBB4[460]	0.8	Erb-b2 receptor tyrosine kinase 4	A member of the epidermal growth factor receptor family, ErbB4 is a type of receptor tyrosine kinase.[461] Also referred to as HER4, ErbB4 is classified as a type I single transmembrane protein and is composed of ~1300 amino acid residues with a large extracellular and intracellular domain.[462] ErbB4 has been implicated in various types of cancer including breast, lung, and colorectal cancer, among others.[463, 464]	Mutations in ERBB4 have been reported in ALS patients.[465-468] ErbB4 binds to and interacts with a number of partners, one of which is neuregulin, small cell signaling protein molecules,[469] and undergo dimerization accompanied by autophosphorylation.[470] Mutations associated with ALS in the tyrosine kinase or C terminal domain of ErbB4 was accompanied by reduced autophosphorylation.[466]
CCNF[471]	0.7	Cyclin F [471]	Cyclins are proteins that regulate the activity of cyclin-dependent protein kinases (CDK).[472] Described first in 1994 by Bai et al,[472] cyclin F is composed of ~786 amino acid residues and is structurally similar to other cyclins (especially A and B) with a wide distribution profile. Cyclin F is a part of the Skp1–Cul1–F-box (SCF) E3 ubiquitin ligase complex and is involved in ubiquitination of proteins[473] with ubiquitinated protein eventually being degraded by the ubiquitin-proteasome system. Cyclin F has been implicated in cancer.[474-476]	Similar to other proteins important for autophagy such as TDP-43 and p62/ sequestosome 1, mutations in CCNF has been observed in and linked to ALS.[403, 477-480] Mutant CCNF results in abnormal ubiquitination, and this is thought to play a role in ALS.[477, 478] Another mechanism/pathway that may be important in ALS involves mutant cyclin F increasing the ATPase activity of valosin-containing protein eventually resulting in TDP-43 aggregation.[481]
ANXA11[482]	0.7	Annexin A11	Annexin A11 is a ~504 amino acid residue long Ca^{2+}-dependent membrane protein that is wide expressed.[483] Annexin A11 is thought to be important for vesicle trafficking by aiding in stabilization of Sec31A, a component of the coat protein complex II.[484] In 2023, the full-length structure of annexin A11 was published[485] and is bound to help in understanding how disease causing mutations affect the protein. Annexin A11 appears to play a role in sarcoidosis, an inflammatory disorder.[483, 486] Another disease	As with other genes noted here, mutations in ANXA11 have been reported in the context of ALS.[489-493] The various mechanisms by which mutant annexin A11 results in ALS included formation of insoluble aggregates, disruption of Ca^{2+} homeostasis,[494, 495] and interference with RNA transport.[496] Structural studies of annexin A11 including crystal structure[497] have been reported that suggest possible effects of

Gene	Association score	Protein	Expression profile, function and other diseases	Amyotrophic lateral sclerosis (ALS)
			with possible annexin A11 involvement is immunoglobulin G4-related disease.[487, 488]	mutation on the structure and functionality of annexin A11.[498]
NEK1[499]	0.6	NIMA related kinase 1	The never in mitosis A (NIMA)-related kinase 1 is a serine/threonine kinase.[499] It is highly expressed in neuronal and germ cells[500, 501] and plays a role in several cellular processes such as cell cycle regulation, cell death, cilia formation, and DNA damage response.[502, 503] NEK1 mutations appear to be associated with Mohr syndrome,[504] short rib-polydactyly syndrome, Majewski type,[505] and axial spondylometaphyseal dysplasia.[506]	Different NEK1 mutations have been discovered and associated with ALS in various cohorts.[507] Two main mutations, NEK1 loss-of-function variants and missense variants such as p.Arg261His have been discovered and are believed to be associated with ALS.[508-511]

Table S2. List of genes associated with Huntington's disease (HD), as shown in Figure 3B, with the association type as causal or contributing.

Gene	Association score	Protein	Expression profile, function and other diseases	Huntington's disease (HD)
BDNF[512]	0.9	Brain derived neurotrophic factor	Brain derived neurotrophic factor are small proteins expressed widely through various parts of the brain[513, 514] and important for synaptic plasticity.[514] Implicated in psychiatric illness.[515]	Reduction in levels of BDNF are observed in HD.[516, 517] Targeting BDNFs is also a potential therapeutic strategy in HD. [518, 519] Increasing BDNF levels are indirectly potentially beneficial in treating HD.[520, 521] There are conflicting reports about the utility of BDNF as a potential biomarker for HD.[522-524]
SIRT1[525]	0.8	Sirtuin 1	Sirtuin is a type of deacetylase that is dependent on nicotinamide adenosine dinucleotide (NAD)[526] with a wide expression profile (brain as well as other organs such as kidney, liver, pancreas, spleen).[527] Sirtuin 1 appears to have a role in obesity [528], metabolic disorders including diabetes,[529, 530] as well as immune response.[531]	Sirtuin 1 appear to have a neuroprotective role in HD.[532, 533] Modulation of sirtuin 1 activity is being explored as viable therapeutic strategy[533-536] with arguments being made for sirtuin 1 activation [537] as well as inhibition.[538] Selisistat (CAS RN: 49843-98-3) was being developed as a small molecule inhibitor of sirtuin 1 for the treatment of HD.[539, 540]
HAP1[541]	0.8	Huntingtin associated protein 1	Huntingtin associated protein 1 is expressed in various areas of the CNS including amygdala and hypothalamus among others. [542] HAP1 found to be important for trafficking of cellular components.[543, 544] HAP1 is also implicated in various neurodegenerative diseases such as Alzheimer's, Parkinson's as well as rare diseases such as ALS. [545]	HAP1 protein has been shown to interact with mutant huntingtin protein.[545] The HAP1-mHTT association impairs normal/physiological cellular trafficking carried out by HAP1 in conjunction with other proteins.[546, 547] There are contradictory studies indicating HAP1 has a bearing on age of onset.[548, 549]
PPARGC 1A[550]	0.8	Peroxisome proliferator-activated receptor (PPAR)-γ coactivator 1 α (PGC-1α)	PGC-1α acts as a coactivator to PPARα and plays a role in mitochondrial energy metabolism.[551] PGC-1α has been studied in the context of physical fitness especially in athletes. [552-556] Recently, PGC-1α has been linked to Parkinson's disease. [557, 558]	Impaired levels of PGC-1α are observed in HD[559] with overexpression of PGC-1α allowing rescue in HD mice.[560] Mutant huntingtin protein is shown to inhibit PGC-1α expression.[561] Studies suggest that PGC-1α may have an impact on age of onset of HD.[562, 563]

Gene	Association score	Protein	Expression profile, function and other diseases	Huntington's disease (HD)
CNTF[564]	0.8	Ciliary neurotrophic factor	Ciliary neurotrophic factor is a cytokine belonging to the IL-6 family. [565] Expressed in Schwann cells as well as T cells. [566] The are also important for the survival of oligodendrocytes [567] and have been implicated in CNS diseases involving demyelination [568-570] as well as motor neuron disease. [571]	CNTF appears to have a neuroprotective role in HD.[572] Administration of CNTF has been explored as a potential treatment for HD with varying results.[573-576]
IGF1[577]	0.8	Insulin-like growth factor 1	Also referred to as somatomedin C or somatomedin 1,[578] IGF1 is a small protein of 70 amino acid residues[579] secreted primarily by the liver.[580] Acts as a mediator of growth hormone and is critical in somatic growth.[580] Elevated IGF1 levels has been linked to rare disorders such as gigantism and acromegaly.[581] IGF1 has also been studied in the context of aging.[582, 583]	IGF1 has been associated with declining social cognition in HD[584] with IGF1 levels as potential indicators.[585] Targeting IGF1 has also been explored as a therapeutic strategy in HD with both IGF1 inhibitors[586] as well as administration of IGF1 itself having been studied.[587]

Table S3. List of genes associated with myasthenia gravis (MG), as shown in Figure 3C, with the association type as causal or contributing.

Gene	Association score	Protein	Expression profile, function and other diseases	Myasthenia gravis (MG)
AGRN [588]	0.4	Agrin	Agrin is a glycosylated protein involved in activation of MuSK and the development of NMJs. [589] Two isoforms of agrin exist – neural agrin and muscle agrin. [589] Abnormalities in agrin have been observed in Alzheimer's [590, 591] and Parkinson's [592] diseases and agrin targeted therapy has also been suggested for treatment of cardiac injury. [593] Agrin's role in immune system has also been explored. [594]	Autoantibodies against agrin have been detected in individuals suffering from MG. [589, 595-597] Mice immunized with agrin generated agrin antibodies and exhibited reduced muscle weakness along with morphological changes in NMJs. [598]
LRP4 [599]	0.4	Low-density lipoprotein (LDL) receptor related protein 4	LDL receptor related protein 4 is a receptor with a single transmembrane domain that binds to neural agrin and induces activation of MuSK leading to development of NMJs. [589] LRP4 has been implicated in neurological disorders. [600]	Similar to agrin, autoantibodies against LRP4 have been detected in individuals with MG [597, 601-603] – especially true of individuals suffering from double-seronegative myasthenia gravis.
MUSK [604]	0.4	Muscle associated receptor tyrosine kinase (MuSK)	The agrin-LRP4 complex binds to and activates MuSK, a receptor tyrosine kinase, which in turn interacts with other proteins (including docking-protein 7 and tumorous imaginal disc 1) to eventually cause clustering of acetylcholine receptors in the NMJs. [605, 606] Similar to agrin, MuSK is also a single transmembrane domain protein. [607] Involvement of MUSK has been studied in the context of muscle disorders including congenital myasthenic syndrome [608, 609] as well as fetal akinesia deformation sequence (FADs) [610, 611] a rare lethal disorder affecting infants. [612, 613]	MuSK-MG is a subtype of MG characterized by an acute onset [607, 614] and seemingly appear to be associated with worse outcomes than AChR antibody MG. [615] MuSK-MG tends to show greater prevalence among females [616] with recent studies published by researchers in China showing similar trends. [617, 618] Autoantibodies against MuSK prevent binding of LRP4 to MuSK disrupting NMJs. [619]
TTN [620]	0.4	Titin	Titin is one of the largest proteins in the human body and is composed of 27,000-34,000 amino acid residues. [621] Along with actin and myosin, titin is integral for muscle contraction. [622] Diseases arising due to mutations in the TTN gene are referred to as titanopathy and characterized by muscle weakness. [623-625]	Autoantibodies against titin have been reported in MG patients with thymoma. [626-628]

Table S4. Rare disease therapeutic drug candidates in commercial preclinical development (Source: https://clinicalintelligence.citeline.com/)

Therapy type	Drug	Mechanism	Company, location
**Amyotrophic lateral sclerosis**			
Antibody therapy	VTX-001	Protein degrader, targeting oxidized phosphatidylcholines	VectoryTx, Netherlands
	VTX-002	Protein degrader; TDP-43 inhibitor	VectoryTx, Netherlands
Antisense oligonucleotide therapy	ALS therapy, AcuraStem	PIKFYVE inhibitor	AcuraStem, USA
	ALS therapy, AcuraStem	SYF2 pre-mRNA splicing factor antagonist	AcuraStem, USA
	ALS therapy, AcuraStem	Unc-13 homolog A agonist	AcuraStem, USA
	ALS therapy, Genetic Leap-1	Targeting FLCN	Genetic Leap, USA
	ALS therapy, Maze Therapeutics	UNC13A inhibitor, Protein synthesis inhibitor	Maze Therapeutics, USA
	ALS therapy, Regulus	MicroRNA inhibitor	Regulus Therapeutics, USA
	AMX-114	Calpain 2 inhibitor	Amylyx Pharmaceuticals, USA
	AS-202	Phosphatidylinositol 3-phosphate 5-kinase inhibitor; TDP-43 inhibitor	AcuraStem, USA; Takeda, Japan
	BMD-001	MicroRNA inhibitor	Biorchestra, South Korea
	QRL-202	TDP-43 inhibitor	QurAlis, USA
	QRL-204	Undisclosed	QurAlis, USA
CAR T-cell therapy	ALS therapy, PolTREG	T cell stimulant	PolTREG, Poland
Cognition enhancer, neuroprotectant	ADV-368	Sigma 1 receptor agonist, sigma 2 receptor antagonist	Advantx Pharmaceuticals, USA
	AGS-499	Telomerase stimulant	Neuromagen Pharma, Israel
	Alpha-cyclodextrin derivatives	Arachidonic acid inhibitor, down-regulates the activity of the phosphatidylinositol cycle	ASDERA, USA

Therapy type	Drug	Mechanism	Company, location
	ALS therapy, Myrobalan Therapeutics	Colony stimulating factor 1 receptor antagonist	Myrobalan Therapeutics, USA
	ALS therapy, Myrobalan Therapeutics	G protein-coupled receptor 17 antagonist	Myrobalan Therapeutics, USA
	ALS therapy, Nevrargenics	RAR modulators	Nevrargenics, UK
	ALS therapy, WaveBreak	TDP-43 inhibitor	WaveBreak, USA
	ALTA-808	TDP-43 inhibitor	Alteron Therapeutics, USA
	ALS therapy, Sharp Therapeutics	Undisclosed	Sharp Therapeutics, USA
	ALS therapy, Yumanity Therapeutics	Undisclosed	Kineta, USA
	ALS therapy, Yumanity Therapeutics	Undisclosed	Merck & Co., USA
	ALS therapy, Yumanity Therapeutics	Undisclosed frontotemporal lobar dementia therapy	Kineta, USA
	Anhydrous enol-oxaloacetate, Metvital	Undisclosed	MetVital, USA
	Antroquinonol	Ras inhibitor	Golden Biotechnology, Taiwan
	AS-201	Phosphatidylinositol 3-phosphate 5-kinase inhibitor	AcuraStem, USA
	BLX-0279	Undisclosed	Biolexis Therapeutics, USA
	BrAD-R13	TrkB tyrosine kinase stimulant	Shanghai Braegen Pharmaceutical, China
	BSC-3301	Receptor-interacting serine/threonine kinase 1 inhibitor	BiSiChem, South Korea
	FB101	Abl receptor tyrosine kinase inhibitor	1st Biotherapeutics, South Korea
	FB418	Abl receptor tyrosine kinase inhibitor	1st Biotherapeutics, South Korea

Therapy type	Drug	Mechanism	Company, location
	GP-119	Undisclosed	GliaPharm, Switzerland
	INF-11	Undisclosed	Beijing Joekai Biotechnology, China
	IRX-4204	Retinoid X receptor agonist	Io Therapeutics, USA
	Mitometin	Carnitine palmitoyltransferase 1 inhibitor	2N Pharma, Denmark
	NB-001	Parkin E3 ubiquitin ligase stimulant	NysnoBio, USA
	NMRA-CK1d	Casein kinase 1 inhibitor	Neumora Therapeutics, USA
	NVG-300	Undisclosed	NervGen Pharma, Canada
	NX-210c	Beta 1 integrin agonist	Axoltis Pharma, France
	Resveratrol	Apoptosis stimulant; Reducing agent	Jupiter Neurosciences, USA
	Rifampicin	DNA directed RNA polymerase inhibitor	Medilabo RFP, Japan
	Progranulin	Undisclosed	Alpha Cognition, Canada
	Pur XX-01	Beta-N-acetylglucosaminidase inhibitor	PurMinds NeuroPharma, Canada
	SEL-148,742	Undisclosed	Sharp Therapeutics, USA
	SLS-009	Ubiquitin ligase E3 stimulant; Protein degrader	Seelos Therapeutics, USA
	SMB-001	Undisclosed	Symbinas Pharmaceuticals, USA
	SRT-055 series	Mitogen-activated protein kinase kinase kinase 5 inhibitor	Seal Rock Therapeutics, USA
Exosome therapy	AB-126	Reduces neuroinflammation, promotes neuroprotection, and stimulates neuroregeneration	Aruna Bio, USA
Fusion protein therapy	PF-1802	Undisclosed	Immunoforge, South Korea
Fusion protein therapy; Cytokine therapy	COYA-302	CD80 antagonist; CD86 antagonist; Immune checkpoint modulator; Interleukin 2 receptor agonist; T cell inhibitor; T cell stimulant	Coya Therapeutics, USA; Dr. Reddy's Laboratories, India
Gene therapy	ABO-201	Genome editing, C9orf72 inhibitor	Arbor Biotechnologies, USA

Therapy type	Drug	Mechanism	Company, location
	ABO-202	Genome editing, stathmin 2 agonist	Arbor Biotechnologies, USA; 4D Molecular Therapeutics, USA
	ABO-204	Genome editing, superoxide dismutase-1 inhibitor	Arbor Biotechnologies, USA
	ALS therapy, AviadoBio	Gene supplementation, PGRN	AviadoBio, UK
	ALS therapy, Hui-Gene Therapeutics	Genome (DNA) editing	HuidaGene Therapeutics, China
	ALS therapy, Korro Bio	Genome editing, TDP-43 stimulant	Korro Bio, USA
	ALS therapy, Maze Therapeutics	Ataxin 2 inhibitor, Protein synthesis inhibitor	Maze Therapeutics, USA
	ALS therapy, NeuShen Therapeutics	SOD1 silencing	NeuShen Therapeutics, China
	ALS therapy, NeuShen Therapeutics	Undisclosed	NeuShen Therapeutics, China
	ALS therapy, Prime Medicine	Genome editing	Prime Medicine, USA
	ALS therapy, Sangamo Therapeutics	C9orf72 inhibitor, ZFP-TF gene therapy	Alexion, UK; Sangamo Therapeutics, USA
	ALS therapy, Scribe Therapeutics	CRISPR-Cas9 Genome editing	Biogen, USA; Scribe Therapeutics,USA
	AMA-007	Undisclosed	Amarna Therapeutics, Netherlands
	ANEW-202	Undisclosed	Anew Medical, USA
	ANL-303	Undisclosed	ANLBIO, South Korea
	CTx-1000	TDP-43 inhibitor	Celosia Therapeutics, Australia
	CTx-2000	TDP-43 inhibitor	Celosia Therapeutics, Australia
	CTx-TFEBx	Undisclosed	Coave Therapeutics, France
	ET-101	Caveolin stimulant	Eikonoklastes Therapeutics, Japan
	NM-301	Undisclosed	Helixmith, South Korea; Neuromyon, South Korea
	NM-302	Undisclosed	Helixmith, South Korea

Therapy type	Drug	Mechanism	Company, location
	NXL-003	Undisclosed	NeuExcell Therapeutics, USA
	PBAL-05	Undisclosed	Passage Bio, USA
	SNUG-01	Unidentified pharmacological activity	Sineugene, China
	SOL-257	TDP-43 inhibitor	SOLA Biosciences, USA
	SOL-258	Undisclosed	SOLA Biosciences, USA
	VY-SOD102	Superoxide dismutase-1 inhibitor	Voyager Therapeutics, USA
Monoclonal antibody	BT-3814	TDP-43 inhibitor	BioArctic Neuroscience, Sweden
	LP-005	C5 and C3 inhibitor	LongBio Pharma, China
	ND-3014	TDP-43 inhibitor	BioArctic Neuroscience, Sweden
	PAS-003	Undisclosed	Pasithea Therapeutics, USA
	PMN-267	TDP-43 inhibitor	ProMIS Neurosciences, Canada
	Anti-TDP-43 antibody, Prothena	TDP-43 inhibitor	Bristol-Myers Squibb, USA; Prothena, Ireland
Monoclonal antibody, humanized	ALS therapy, Mabylon	Undisclosed	Mabylon, Switzerland; SciNeuro Pharmaceuticals, USA
	ARGX-119	Muscle-specific receptor kinase stimulant	Argenx, Netherlands
	EME-023	Undisclosed	Epsilon Molecular Engineering, Japan
	Foralumab	CD3 antagonist	Tiziana Life Sciences, UK
	Hu-GNK-301	Undisclosed	GeNeuro, Switzerland
	NC-B8	IgG4 inhibitor	ENCEFA, France
	NI-308	Immunostimulant	Neurimmune, Switzerland
Monoclonal antibody, murine	ALS therapy, ProMIS Neurosciences	Protein kinase C inhibitor, RACK1-targeting antibodies	ProMIS Neurosciences, Canada
	ALS therapy, ProMIS Neurosciences	SOD1-targeting antibodies, Superoxide dismutase-1 inhibitor	ProMIS Neurosciences, Canada
Neuroprotectant	AKV-9	Protein aggregation inhibitor	Akava Therapeutics, USA

Therapy type	Drug	Mechanism	Company, location
	ALS therapy, Amylyx Pharmaceuticals	Bax and Bak protein inhibitors	Amylyx Pharmaceuticals, USA
	ALS therapy, Dewpoint Therapeutics	TDP-43 inhibitor	Dewpoint Therapeutics, USA
	ALS therapy, Genetic Leap-2	RNA binder targeting FLCN	Genetic Leap, USA
	ALS therapy, ImmunoBrain Checkpoint	Immune checkpoint modulator	ImmunoBrain Checkpoint, Israel
	ALS therapy, Miramoon Pharma	Ryanodine receptor antagonist; Oxygen scavenger	Miramoon Pharma, Spain
	ALS therapy, Nine Square Therapeutics	Undisclosed	Nine Square Therapeutics, USA
	ALS therapy, NRG Therapeutics	mPTP inhibitor	NRG Therapeutics, UK
	ALS therapy, Origami Therapeutics	Undisclosed	Origami Therapeutics, USA
	ALS therapy, Path Biotech	Undisclosed	Path Biotech, USA
	ALS therapy, Pikralida	Undisclosed	Pikralida, Poland
	ALS therapy, PrecisionLife	Undisclosed	PrecisionLife, UK
	ALS therapy, reMYND	Undisclosed	reMYND, Belgium
	ALS therapy, Treventis	TDP-43 inhibitor	Treventis, Canada
	ALS therapy, Zhittya Genesis Medicine	Fibroblast growth factor 1 agonist	Zhittya Genesis Medicine, USA
	Amisodin	Superoxide dismutase-1 inhibitor	Pioneer of Rare Genopathies, South Korea
	ALS therapy, Verge Genomics-1	Undisclosed	Eli Lilly, USA
	ALS therapy, Verge Genomics-1	Undisclosed	Verge Genomics, USA
	ATC-104	Protein degrader; Sequestosome 1 stimulant; TDP-43 inhibitor	Autotac Bio, South Korea
	ATH-1105	Undisclosed	Athira Pharma, USA

Therapy type	Drug	Mechanism	Company, location
	BEN-34712	Retinoic acid alpha receptor agonist; Retinoic acid beta receptor agonist	BenevolentAI, UK
	BIOIO-1001	Sirtuin 3 stimulant	BIOIO, USA
	BL-002, Bloom Science	Microbiome modulator, live microorganisms	Bloom Science, USA
	BREN-02	Undisclosed	BrainEver, France
	BT-003	Undisclosed	BAKX Therapeutics, USA
	CB03-154	Potassium channel agonist	Shanghai Zhimeng Biopharma, China
	Donepezil hydrochloride + nebivolol hydrochloride	Acetylcholinesterase inhibitor; Beta 1 adrenoreceptor antagonist	Dr. Noah Biotech, South Korea
	EVT-8683	Protein degrader; Translation initiation factor 2B stimulant	Bristol-Myers Squibb, USA
	EVT-8683	Protein degrader; Translation initiation factor 2B stimulant	Evotec, Germany
	FB-1002	Unidentified pharmacological activity	4B Technologies, China
	FP-802	Transient receptor potential cation channel M antagonist; NMDA receptor antagonist	FundaMental Pharma, Germany
	Glycolic acid + D-lactic acid	Undisclosed	Neurevo, Germany
	HLX-94	Undisclosed	Shanghai Fosun Pharmaceutical, China
	HS-03	Undisclosed	Foshan Rexiu Biotechnology, China
	ILB	Undisclosed	TikoMed, Sweden
	iN3011-K23	Undisclosed	iN Therapeutics, South Korea
	IPG 008	Hydrolase inhibitor	Nanjing Immunophage Biotech, China
	KFRX-03	Tyrosine kinase inhibitor (TKI)	KeifeRx, USA
	LAUR-301	Glial cell derived neurotrophic growth factor agonist	Lauren Sciences, USA
	M-102	Undisclosed	Aclipse Therapeutics, USA
	mecobalamin	Vitamin B12 agonist	Eisai, Japan
	MP-101	Undisclosed	Mitochon Pharmaceuticals, USA

Therapy type	Drug	Mechanism	Company, location
	MT-2	Undisclosed	MimeTech, Italy
	Nibrozetone (RRx-001)	NLRP3 inhibitor and Nrf2 upregulator	EpicentRx, USA
	OC-514	Undisclosed	Oncocross, South Korea
	ORY-4001	Histone deacetylase 6 inhibitor	Oryzon, Spain
	otaplimastat	Undisclosed	Shin Poong Pharmaceutical, South Korea
	P-202320	Undisclosed	EDDC, Singapore
	Pimicotinib	Colony stimulating factor 1 receptor antagonist; Immuno-oncology therapy	Sperogenix Therapeutics, China
	Riluzole	Dopamine receptor agonist; Glutamate antagonist; Voltage-gated sodium channel antagonist	Brain Trust Bio, USA
	RT-1968	Undisclosed	Raya Therapeutic, Canada
	RT-1972	Undisclosed	Raya Therapeutic, Canada
	RT-1978	Undisclosed	Raya Therapeutic, Canada
	RT-1999	Undisclosed	Raya Therapeutic, Canada
	RT-2010	Undisclosed	Raya Therapeutic, Canada
	RVL-027	Undisclosed	Unravel Biosciences, USA
	SAM-001	Transient receptor potential cation channel member 1 agonist	Samsara Therapeutics, UK
	SAM-19272	GTPase stimulant	Samsara Therapeutics, UK
	SNP-210	TDP-43 inhibitor	SciNeuro Pharmaceuticals, USA
	S-oxprenolol	Beta adrenoreceptor antagonist	Actimed Therapeutics, UK
	TDP-43 inhibitors, Aquinnah	TDP-43 inhibitor	Aquinnah Pharmaceuticals, USA; Roche, Switzerland
	WD-920	Undisclosed	Zhejiang Wenda Medical Technology, China
	Y-8	Undisclosed	Neurodawn Pharmaceutical, China
RNA interference	ALN-SOD1	Gene expression inhibitor, Superoxide dismutase-1 inhibitor	Alnylam, USA; Regeneron, USA
	ALS therapy, AviadoBio	Gene expression inhibitor, ATXN2	AviadoBio, UK

Therapy type	Drug	Mechanism	Company, location
	ALS therapy, AviadoBio	Gene expression inhibitor, c9orf72	AviadoBio, UK
	ALS therapy, AviadoBio	Gene expression inhibitor, SOD1	AviadoBio, UK
	AMT-161	C9orf72 inhibitor; Gene expression inhibitor	uniQure, Netherlands
Stem cell therapy	ALS therapy, Neuroplast	Undisclosed	Neuroplast, Netherlands
	ALS therapy, Takeda	Undisclosed	Takeda, Japan
	BMS-xxxx	Not applicable	Bristol-Myers Squibb, USA; Evotec, Germany
	Cognistem	Undisclosed	Amniotics, Sweden
	CUR-201	Undisclosed	Curamys, South Korea
	CUR-202	Undisclosed	Curamys, South Korea
	CUR-204	Undisclosed	Curamys, South Korea
	hNPC-02	Not applicable	Hopstem Biotechnology, China
	NCP-01	Undisclosed	Hemostemix, Canada
**Huntington’s disease**			
Antibody therapy	VTX-003	mHTT protein inhibitor, selectively binds mutant HTT (and not normal HTT) to clear mutant HTT	VectorY Therapeutics, Netherlands
Anti inflammatory	IC 100-05	Inflammasome ASC Inhibitor	ZyVersa Therapeutics, USA
Antisense oligonucleotide therapy	Huntington's disease therapy, Ionis	Undisclosed	Ionis, USA; Roche, Switzerland
Cognition enhancer	ASK-005	Arachidonic acid inhibitor	ASDERA, USA
Cognition enhancer, neuroprotectant	Huntington's disease therapy, MitoRx	Reverses mitochondrial dysfunction	MitoRx Therapeutics, UK
	NP-001	Oxidizing agent	Neuraltus, USA; Neuvivo, USA
Gene therapy	ET-101	Caveolin stimulant	Eikonoklastes Therapeutics, USA
	Huntington's disease therapy, Hudiagene	DNA editing, CRISPR	HuidaGene Therapeutics, China

Therapy type	Drug	Mechanism	Company, location
	Huntington's disease therapy, Life edit	Genome editing targeting both the T and C alleles of an exonic SNP in the HTT gene utilizing AAV vector	Life Edit Therapeutics, USA
	Huntington's disease therapy, Passage	Deliver functional gene utilizing AAV vector	Passage Bio, USA
	Huntington's disease therapy, Prime	Genome editing	Prime Medicine, USA
	NXL-002	Regenerates neurons	NeuExcell Therapeutics, USA; Spark Therapeutics, USA
	SOL175	Reduces abnormally folded protein	SOLA Biosciences, USA
	INT41	mHTT protein inhibitor, selectively binds to mHTT protein	Vybion, USA
	SOL176	Reduces abnormally folded protein	SOLA Biosciences, USA
	TAK-686	Zinc finger nucleases can target regions of DNA to modify them or stop RNA from being made	Sangamo Therapeutics, USA; Takeda, Japan
	ReS18-H	Restores function and improve survival of medium spiny neurons leading to reactivation of corticostriatal transmission	reMYND, Belgium
Monoclonal antibody, humanized	ATLX-1095	HTT inhibitor	Alchemab Therapeutics, UK
Monoclonal antibody	Huntington’s disease therapy ProMIS	Targeting protein RACK-1, Protein kinase C inhibitor	ProMIS Neurosciences, USA
mRNA therapy	Anima Huntingtin translation inhibitor	Gene expression inhibitor	Anima Biotech, USA; Takeda, Japan
Neuroprotectant	AJ-201	Transcription factor Nrf2 stimulant	Avenue Therapeutics, USA
	Huntington's disease therapy, BPGbio	Undisclosed	BPGbio, USA
	Huntington’s disease therapy LoQus23	DNA damage repair	LoQus23 Therapeutics, USA
	M102	Activates Nrf2 and HSF1	Aclipse Therapeutics, USA
	TQS-168	PPARG coactivator 1 alpha agonist	Tranquis Therapeutics, USA

Therapy type	Drug	Mechanism	Company, location
Peptide therapy	TT-P34	Activates pathways that can bypass mHTT to reactivate CREB	Teitur Trophics, Denmark
	NT-0100	Gene expression inhibitor	NeuBase Therapeutics, USA
PROTAC	Huntington's disease therapy, Arvinas	HTT inhibitor, E3 ubiquitin ligase stimulant, protein degrader	Arvinas, USA
Protein degrader	ORI-113	HTT protein degrader	Origami Therapeutics, USA
Protein conformation correctors	ORI-503	HTT proteins conformation corrector	Origami Therapeutics, USA
Protein degrader	SLS009	Protein targeted autophagy	Seelos Therapeutics, USA
RNA interference	ALN-HTT	Gene expression inhibitor	Alnylam, USA
	ALN-HTT02	Gene expression inhibitor	Alnylam, USA
	Huntington’s disease therapy Atalanta	Gene expression inhibitor	Atalanta Therapeutics, USA; Biogen, USA
	Huntington's disease therapy, novartis	Utilized a small hairpin RNA or short hairpin RNA for gene expression inhibition	Novartis, Switzerland; Voyager Therapeutics, USA
	OCCT-HTT siRNA	Gene expression inhibitor, HTT inhibitor	Ophidion, USA
RNA therapy	SMDG-HD11	Undisclosed	S. M. Discovery Group, UK
Stem cell therapy	Debamestrocel	Glial cell derived neurotrophic growth factor agonist	BrainStorm Cell Therapeutics, USA
	HB AdMSC	Undisclosed	Hope Biosciences, USA
	Huntington's disease therapy, trailhead	Mesenchymal Stem Cells	Trailhead Biosystems, USA
	SC-379	Glial progenitor cells	Sana Biotechnology, USA
Undisclosed	Huntington's disease therapy Aitia	Undisclosed	UCB, Belgium; Aitia, USA
**Myasthenia gravis**			
CAR T-cell therapy	CABA-201	T cell stimulant	Cabaletta Bio, USA
	Equecabtagene autoleucel	Immuno-oncology therapy; T cell stimulant	Nanjing IASO Biotechnology, China

Therapy type	Drug	Mechanism	Company, location
	KYV-101	T cell stimulant	Kyverna Therapeutics, USA
Fusion protein therapy	Myasthenia gravis therapy, Rongchang Pharmaceuticals	Lymphocyte stimulant	Rongchang Pharmaceuticals, China
	TOL-2	Undisclosed	Toleranzia, Sweden
Monoclonal antibody	EA-5	Complement inhibitor	Lan-yi Therapeutics, China
	LP-005	C5a inhibitor	LongBio Pharma, China
	MV-2C2	Survivin inhibitor	MimiVax, USA
Monoclonal antibody, humanized	CAN-106	Complement factor C5 inhibitor	CANbridge Life Sciences, China
	MIL-62	CD20 antagonist; Immuno-oncology therapy	Beijing Mabworks Biotech, China
	Pozelimab	C5a inhibitor	Regeneron, USA
Musculoskeletal therapy	BHV-1310	Protein degrader	Biohaven, British Virgin Islands
	Myasthenia gravis therapy, BioCryst Pharmaceuticals	Complement factor C5 inhibitor	BioCryst Pharmaceuticals, USA
	CNP-MYG	B-cell inhibitor; CD8 antagonist	Cour Pharmaceuticals, USA
	CV-MG-02	Immunostimulant	CuraVac, USA
	Myasthenia gravis vaccine, ImCyse	Immunosuppressant	ImCyse, Belgium
	Myasthenia gravis therapy, NovelMed Therapeutics	Complement inhibitor	NovelMed Therapeutics, USA
	prednisone, Sarcomed AB	Glucocorticoid agonist	Sarcomed AB
	ZP-10068	Complement C3 inhibitor	Alexion, UK;<br>Zealand Pharma, Denmark

Table S5. List of notable patents pertaining to the three rare diseases – ALS, HD, and MG – identified from the CAS Content Collection.

Patent Number	Year	Patent assignee, location	Description
**Amyotrophic lateral sclerosis**			
US20200002723	2020	Deutsches Krebsforschungszentrum, Germany	It describes nucleotide sequences called MSBI (Multiple Sclerosis Brain Isolate) as well as probes, primers, and antibodies against polypeptides encoded by MSBI sequences. These could serve as early markers for the future development of cancer and diseases of the CNS (multiple sclerosis, prion-linked diseases, ALS, transmissible spongiform encephalitis, Parkinson's disease, and Alzheimer's disease).
WO2020010049	2020	The General Hospital Corporation, AZ Therapies, Inc., USA	It describes a composition comprising micronized cromolyn sodium, α-lactose, and salt of fatty acid (preferably magnesium stearate) used to treat certain neurological diseases including Alzheimer’s disease, ALS, and Parkinson’s disease.
CN117050134	2023	Shanghai Institute of Organic Chemistry, Chinese Academy of Sciences, China	It describes a novel oleanamide derivative for activating a KEAP/NRF2/ARE signaling pathway which can be used for treating and preventing various neurological disorders including ALS.
EP4255406A1	2020	Massey Ventures Ltd, New Zealand	It relates to (2S)-2-Aminopentanethioic S-acid or a pharmaceutically acceptable salt as a medication for the treatment of ALS.
WO2022138707	2022	Eisai R&D Management Co., Ltd., Japan	It describes the development of pharmaceutical composition comprising anti-EphA4 antibodies capable of binding to and promoting the cleavage of EphA4, which is used for treating ALS.
**Huntington’s disease**			
WO2023099648	2023	AstraZeneca AB, Sweden	It describes pyrazolo- and triazolo-azinone compounds that inhibit receptor-interacting protein kinase 1 (“RIPK1”) and can be used in the treatment of neurological disorders including HD.
US20240076310A1	2023	Sage Therapeutics Inc, USA	Neuroactive steroids (or their combinations), that target GABA receptor complex (GRC) can be used for the treatment of neurodegenerative disorders including HD.
WO2022235329A1	2022	University of South Carolina, USA	It comprises hydrophilic nanogels based on polyethylene glycol (PEG) copolymers. The nanogels can encapsulate an antibody for delivery to the brain and can include ligands for blood brain barrier (BBB) receptors on the surface. These nanogels can the treatment of neurological disorders including HD.
WO2022132894A1	2022	Rush University Medical Center, USA	It describes a pharmaceutical composition comprising glycerol tribenzoate and glycerol phenylbutyrate which can be used for treating HD.

Patent Number	Year	Patent assignee, location	Description
WO2020068913A1	2020	Chase Therapeutics Corporation, USA	It describes a combination $5HT_3$ antagonist and/or a NK-I antagonist, in combination with 6-propylamino-4,5,6,7-tetrahydro-l,3-benzothiazole-2-amine and with fluoxetine, zonisamide, or a statin to treat protein misfolding neurodegenerative diseases.
**Myasthenia gravis**			
WO2020106724A1	2020	Alexion Pharmaceuticals, Inc., United States	It describes a formulation that specifically binds complement component 5 (C5) and can be used for treating MG in the pediatric population.
WO2020014072A1	2020	GT Biopharma, Inc., USA	It describes the use of an NK1-antagonist (e.g. aprepitant), in combination with neostigmine, to facilitate the treatment of a patient suffering from MG.
WO2023236967A1	2023	RemeGen Co., Ltd., China	This describes the development of the drug, a dosage regimen, an administration interval, and a mode for treating MG using TACI-Fc fusion protein. It is shown that this formulation exhibits good clinical efficacy and safety in the treatment of MG patients.
WO2020086506A1	2020	Ra Pharmaceuticals, Inc., USA	The present disclosure relates methods of treating MG with zilucoplan (complement inhibitor), including devices and kits available for administering zilucoplan.
CN112048565A	2020	Shijiazhuang People's Hospital, China	The invention discloses a microbial marker (comprising *Megamonas hypermegale* and/or *Fusobacterium mortiferum*) for the diagnosis of MG which offers the advantages of good specificity and high sensitivity.

Table S6. Summary of the outlook and perspectives on ALS, HD, and MG.

	**Amyotrophic lateral sclerosis (ALS)**	**Huntington's disease (HD)**	**Myasthenia gravis (MG)**
**Prognosis**	ALS typically progresses rapidly, with most patients surviving 3 to 5 years after diagnosis. However, some individuals may live much longer, with variations in the rate of disease progression.	Huntington's disease typically manifests in mid-adulthood, with symptoms gradually worsening over 10 to 25 years. The progression and severity of the disease can vary widely among individuals. Juvenile Huntington's disease, which occurs before the age of 20, tends to progress more rapidly.	The course of MG varies widely among individuals. With proper treatment, many people can manage their symptoms and lead relatively normal lives. The disease can fluctuate, with periods of worsening (exacerbations) and improvement (remissions).
**Treatment options**	• Currently, there is no cure for ALS. Treatments aim to slow disease progression, manage symptoms, and improve quality of life.<br>• Riluzole and edaravone are FDA-approved drugs that modestly slow disease progression.<br>• Supportive therapies, including physical therapy, occupational therapy, speech therapy, and nutritional support, are crucial for maintaining function and comfort.	• There is no cure for HD, and treatments focus on managing symptoms and improving quality of life.<br>• Medications such as tetrabenazine and deutetrabenazine are used to treat chorea (involuntary movements). The use of valbenazine, a medication that has shown effectiveness in reducing chorea is awaiting FDA approval.<br>• Antipsychotic drugs, antidepressants, and mood-stabilizing medications help manage psychiatric symptoms and mood disorders.<br>• Physical therapy, occupational therapy, and speech therapy can support motor and functional abilities.	• Anticholinesterase agents (e.g., pyridostigmine) improve communication between nerves and muscles.<br>• Immunosuppressive drugs (e.g., prednisone, azathioprine, mycophenolate mofetil) reduce the abnormal immune response.<br>• Surgical removal of the thymus gland (thymectomy) can improve symptoms in some patients, particularly those with thymomas or generalized MG.<br>• Plasmapheresis and Intravenous Immunoglobulin (IVIg) are used to manage severe exacerbations by removing antibodies from the blood or providing normal antibodies to modulate the immune response.<br>• New therapies, such as monoclonal antibodies (e.g., eculizumab and ravulizumab), target specific components of the immune system.
**Ongoing research**	• **Genetic research:** Advances in genetic research have identified	• **Genetic research:** HD is caused by a mutation in the HTT gene, leading to	• **Targeted therapies:** Researchers are exploring monoclonal antibodies that

	Amyotrophic lateral sclerosis (ALS)	Huntington's disease (HD)	Myasthenia gravis (MG)
	several genes associated with familial ALS, such as SOD1, C9orf72, and TARDBP. Understanding these genetic factors is crucial for developing targeted therapies.<br>• **Stem cell therapy:** Research is exploring the potential of stem cell therapy to replace damaged neurons or protect existing neurons from degeneration. Clinical trials are ongoing to evaluate the safety and efficacy of these approaches.<br>• **Gene therapy:** Gene therapy aims to correct or mitigate the effects of defective genes associated with ALS. Techniques like CRISPR-Cas9 are being investigated for their potential to edit genetic mutations.<br>• **Neuroprotective agents:** Scientists are investigating various compounds and drugs that could protect motor neurons from degeneration. These include anti-inflammatory agents, antioxidants, and mitochondrial protectants.<br>• **Biomarkers:** Identifying biomarkers for early diagnosis and monitoring disease progression is a significant area of research. Reliable biomarkers could improve clinical trials and lead to more effective treatments.	the production of an abnormal huntingtin protein. Research aims to understand how this protein causes neurodegeneration. Studies on gene silencing techniques, such as antisense oligonucleotides (ASOs) and RNA interference (RNAi), are exploring ways to reduce or inhibit the production of the mutant huntingtin protein.<br>• **Stem cell therapy:** Researchers are investigating the potential of stem cell therapy to replace damaged neurons or support the survival of existing neurons. Clinical trials are assessing the safety and efficacy of these approaches.<br>• **Neuroprotective agents:** Various compounds and drugs are being studied for their potential to protect neurons from degeneration. These include antioxidants, anti-inflammatory agents, and compounds targeting cellular energy production.<br>• **Biomarkers:** Identifying biomarkers for early diagnosis and monitoring disease progression is a key area of research. Reliable biomarkers could enhance clinical trials and lead to earlier intervention.	target specific components of the immune system. For example, eculizumab, a complement inhibitor, has shown promise in treating MG by blocking the part of the immune system that attacks the neuromuscular junction.<br>• **Biomarkers:** Identifying biomarkers for MG can help diagnose the disease earlier and monitor the effectiveness of treatments. Ongoing research aims to find reliable biomarkers that can predict disease progression and response to therapy.<br>• **Novel immunosuppressive agents:** New immunosuppressive drugs with fewer side effects and improved efficacy are being developed. These drugs aim to provide better control of the immune system while minimizing adverse effects.<br>• **Gene therapy:** Research into gene therapy for MG is in the early stages, focusing on correcting the underlying genetic causes or modulating the immune response at the genetic level.

	Amyotrophic lateral sclerosis (ALS)	Huntington's disease (HD)	Myasthenia gravis (MG)
**Future perspectives**	• **Precision medicine:** Advances in genetic and molecular profiling may enable personalized treatment approaches tailored to the specific genetic and molecular characteristics of an individual's ALS.<br>• **Combination therapies:** Future treatments may involve combinations of drugs and therapies targeting different aspects of the disease, such as neuroprotection, inflammation reduction, and muscle function enhancement.<br>• **Improved diagnostic tools:** Development of advanced diagnostic tools for early detection and accurate monitoring of ALS progression could lead to earlier intervention and better outcomes.	• **Gene therapy:** Advances in gene therapy hold promise for treating HD by targeting the underlying genetic mutation. Techniques like CRISPR-Cas9 are being explored for their potential to edit or correct the HTT gene mutation.<br>• **Precision medicine:** Personalized treatment approaches based on an individual's genetic and molecular profile could lead to more effective therapies tailored to the specific characteristics of their HD.<br>• **Combination therapies:** Future treatments may involve combining different therapeutic strategies, such as gene silencing, neuroprotective agents, and symptomatic treatments, to address multiple aspects of the disease.<br>• **Improved diagnostic tools:** Development of advanced diagnostic tools for early detection and accurate monitoring of HD progression could lead to earlier and more effective interventions.	• **Personalized medicine:** Advances in genetic and molecular profiling may enable personalized treatment approaches tailored to the specific characteristics of an individual's MG. This could lead to more effective and targeted therapies with fewer side effects.<br>• **Combination therapies:** Future treatments may involve combining different therapeutic strategies, such as immunosuppressive drugs, targeted therapies, and lifestyle interventions, to provide comprehensive management of MG.<br>• **Improved diagnostic tools:** Development of advanced diagnostic tools for early detection and accurate monitoring of MG progression could lead to earlier intervention and better outcomes.

Reference

1. Piñero, J., Queralt-Rosinach, N., Bravo, À., Deu-Pons, J., Bauer-Mehren, A., Baron, M., Sanz, F., and Furlong, L. I. (2015) DisGeNET: a discovery platform for the dynamical exploration of human diseases and their genes. Database 2015.
2. Kovalenko, M., Erdin, S., Andrew, M. A., St Claire, J., Shaughnessey, M., Hubert, L., Neto, J. L., Stortchevoi, A., Fass, D. M., Mouro Pinto, R., et al. (2020) Histone deacetylase knockouts modify transcription, CAG instability and nuclear pathology in Huntington disease mice. Elife 9, e55911.
3. Wilton, D. K., Mastro, K., Heller, M. D., Gergits, F. W., Willing, C. R., Fahey, J. B., Frouin, A., Daggett, A., Gu, X., Kim, Y. A., et al. (2023) Microglia and complement mediate early corticostriatal synapse loss and cognitive dysfunction in Huntington's disease. Nat Med 29, 2866-2884.
4. Narayanaswami, P., Sanders, D. B., Thomas, L., Thibault, D., Blevins, J., Desai, R., Krueger, A., Bibeau, K., Liu, B., Guptill, J. T., and Group, P.-M. S. (2024) Comparative effectiveness of azathioprine and mycophenolate mofetil for myasthenia gravis (PROMISE-MG): a prospective cohort study. Lancet Neurol 23, 267-276.
5. Suster, D. I., Craig Mackinnon, A., DiStasio, M., Basu, M. K., Pihan, G., and Suster, S. (2022) Atypical thymomas with squamoid and spindle cell features: clinicopathologic, immunohistochemical and molecular genetic study of 120 cases with long-term follow-up. Mod Pathol 35, 875-894.
6. Xu, D., Jin, T., Zhu, H., Chen, H., Ofengeim, D., Zou, C., Mifflin, L., Pan, L., Amin, P., Li, W., et al. (2018) TBK1 Suppresses RIPK1-Driven Apoptosis and Inflammation during Development and in Aging. Cell 174, 1477-1491 e1419.
7. Paganoni, S., Macklin, E. A., Hendrix, S., Berry, J. D., Elliott, M. A., Maiser, S., Karam, C., Caress, J. B., Owegi, M. A., Quick, A., et al. (2020) Trial of Sodium Phenylbutyrate-Taurursodiol for Amyotrophic Lateral Sclerosis. N Engl J Med 383, 919-930.
8. Raheja, R., Regev, K., Healy, B. C., Mazzola, M. A., Beynon, V., Von Glehn, F., Paul, A., Diaz-Cruz, C., Gholipour, T., Glanz, B. I., et al. (2018) Correlating serum micrornas and clinical parameters in amyotrophic lateral sclerosis. Muscle Nerve 58, 261-269.
9. International Multiple Sclerosis Genetics, C., and Multiple, M. S. C. (2023) Locus for severity implicates CNS resilience in progression of multiple sclerosis. Nature 619, 323-331.
10. Fitzgerald, K. C., Smith, M. D., Kim, S., Sotirchos, E. S., Kornberg, M. D., Douglas, M., Nourbakhsh, B., Graves, J., Rattan, R., Poisson, L., et al. (2021) Multi-omic evaluation of metabolic alterations in multiple sclerosis identifies shifts in aromatic amino acid metabolism. Cell Rep Med 2, 100424.
11. Baughn, M. W., Melamed, Z., Lopez-Erauskin, J., Beccari, M. S., Ling, K., Zuberi, A., Presa, M., Gonzalo-Gil, E., Maimon, R., Vazquez-Sanchez, S., et al. (2023) Mechanism of STMN2 cryptic splice-polyadenylation and its correction for TDP-43 proteinopathies. Science 379, 1140-1149.
12. Bloom, A. J., Mao, X., Strickland, A., Sasaki, Y., Milbrandt, J., and DiAntonio, A. (2022) Constitutively active SARM1 variants that induce neuropathy are enriched in ALS patients. Mol Neurodegener 17, 1.
13. Baxi, E. G., Thompson, T., Li, J., Kaye, J. A., Lim, R. G., Wu, J., Ramamoorthy, D., Lima, L., Vaibhav, V., Matlock, A., et al. (2022) Answer ALS, a large-scale resource for sporadic and familial ALS combining clinical and multi-omics data from induced pluripotent cell lines. Nat Neurosci 25, 226-237.
14. Perez, R. K., Gordon, M. G., Subramaniam, M., Kim, M. C., Hartoularos, G. C., Targ, S., Sun, Y., Ogorodnikov, A., Bueno, R., Lu, A., et al. (2022) Single-cell RNA-seq reveals cell type-specific molecular and genetic associations to lupus. Science 376, eabf1970.
15. Hasni, S. A., Gupta, S., Davis, M., Poncio, E., Temesgen-Oyelakin, Y., Carlucci, P. M., Wang, X., Naqi, M., Playford, M. P., Goel, R. R., et al. (2021) Phase 1 double-blind randomized safety trial of the Janus kinase inhibitor tofacitinib in systemic lupus erythematosus. Nat Commun 12, 3391.
16. Li, C., Georgakopoulou, A., Newby, G. A., Chen, P. J., Everette, K. A., Paschoudi, K., Vlachaki, E., Gil, S., Anderson, A. K., Koob, T., et al. (2023) In vivo HSC prime editing rescues sickle cell disease in a mouse model. Blood 141, 2085-2099.
17. Jones, R. J., and DeBaun, M. R. (2021) Leukemia after gene therapy for sickle cell disease: insertional mutagenesis, busulfan, both, or neither. Blood 138, 942-947.

18. Richardson, P. G., Oriol, A., Larocca, A., Blade, J., Cavo, M., Rodriguez-Otero, P., Leleu, X., Nadeem, O., Hiemenz, J. W., Hassoun, H., et al. (2021) Melflufen and Dexamethasone in Heavily Pretreated Relapsed and Refractory Multiple Myeloma. J Clin Oncol 39, 757-767.
19. Hideshima, T., and Anderson, K. C. (2021) Signaling Pathway Mediating Myeloma Cell Growth and Survival. Cancers (Basel) 13, 216.
20. Breen, W. G., Hathcock, M. A., Young, J. R., Kowalchuk, R. O., Bansal, R., Khurana, A., Bennani, N. N., Paludo, J., Villasboas Bisneto, J. C., Wang, Y., et al. (2022) Metabolic characteristics and prognostic differentiation of aggressive lymphoma using one-month post-CAR-T FDG PET/CT. J Hematol Oncol 15, 36.
21. Wagner-Johnston, N. D., Sharman, J., Furman, R. R., Salles, G., Brown, J. R., Robak, T., Gu, L., Xing, G., Chan, R. J., Rajakumaraswamy, N., and Gopal, A. K. (2021) Idelalisib immune-related toxicity is associated with improved treatment response. Leuk Lymphoma 62, 2915-2920.
22. Jhelum, P., Santos-Nogueira, E., Teo, W., Haumont, A., Lenoel, I., Stys, P. K., and David, S. (2020) Ferroptosis Mediates Cuprizone-Induced Loss of Oligodendrocytes and Demyelination. J Neurosci 40, 9327-9341.
23. Rojas, O. L., Probstel, A. K., Porfilio, E. A., Wang, A. A., Charabati, M., Sun, T., Lee, D. S. W., Galicia, G., Ramaglia, V., Ward, L. A., et al. (2019) Recirculating Intestinal IgA-Producing Cells Regulate Neuroinflammation via IL-10. Cell 176, 610-624 e618.
24. Cortese, R., Tur, C., Prados, F., Schneider, T., Kanber, B., Moccia, M., Wheeler-Kingshott, C. A. G., Thompson, A. J., Barkhof, F., and Ciccarelli, O. (2021) Ongoing microstructural changes in the cervical cord underpin disability progression in early primary progressive multiple sclerosis. Mult Scler 27, 28-38.
25. Desai, R. A., Davies, A. L., Del Rossi, N., Tachrount, M., Dyson, A., Gustavson, B., Kaynezhad, P., Mackenzie, L., van der Putten, M. A., McElroy, D., et al. (2020) Nimodipine Reduces Dysfunction and Demyelination in Models of Multiple Sclerosis. Ann Neurol 88, 123-136.
26. Caron, N. S., Banos, R., Yanick, C., Aly, A. E., Byrne, L. M., Smith, E. D., Xie, Y., Smith, S. E. P., Potluri, N., Findlay Black, H., et al. (2021) Mutant Huntingtin Is Cleared from the Brain via Active Mechanisms in Huntington Disease. J Neurosci 41, 780-796.
27. O'Day, D. H. (2022) Calmodulin Binding Domains in Critical Risk Proteins Involved in Neurodegeneration. Curr Issues Mol Biol 44, 5802-5814.
28. O'Regan, G. C., Farag, S. H., Casey, C. S., Wood-Kaczmar, A., Pocock, J. M., Tabrizi, S. J., and Andre, R. (2021) Human Huntington's disease pluripotent stem cell-derived microglia develop normally but are abnormally hyper-reactive and release elevated levels of reactive oxygen species. J Neuroinflammation 18, 94.
29. Scahill, R. I., Zeun, P., Osborne-Crowley, K., Johnson, E. B., Gregory, S., Parker, C., Lowe, J., Nair, A., O'Callaghan, C., Langley, C., et al. (2020) Biological and clinical characteristics of gene carriers far from predicted onset in the Huntington's disease Young Adult Study (HD-YAS): a cross-sectional analysis. Lancet Neurol 19, 502-512.
30. Mai, L., Asaduzzaman, A., Noamani, B., Fortin, P. R., Gladman, D. D., Touma, Z., Urowitz, M. B., and Wither, J. (2021) The baseline interferon signature predicts disease severity over the subsequent 5 years in systemic lupus erythematosus. Arthritis Res Ther 23, 29.
31. Munoz-Grajales, C., Prokopec, S. D., Johnson, S. R., Touma, Z., Ahmad, Z., Bonilla, D., Hiraki, L., Bookman, A., Boutros, P. C., Chruscinski, A., and Wither, J. (2022) Serological abnormalities that predict progression to systemic autoimmune rheumatic diseases in antinuclear antibody-positive individuals. Rheumatology (Oxford) 61, 1092-1105.
32. Bradford, H. F., Haljasmagi, L., Menon, M., McDonnell, T. C. R., Sarekannu, K., Vanker, M., Peterson, P., Wincup, C., Abida, R., Gonzalez, R. F., et al. (2023) Inactive disease in patients with lupus is linked to autoantibodies to type I interferons that normalize blood IFNalpha and B cell subsets. Cell Rep Med 4, 100894.
33. Isenberg, D., Furie, R., Jones, N. S., Guibord, P., Galanter, J., Lee, C., McGregor, A., Toth, B., Rae, J., Hwang, O., et al. (2021) Efficacy, Safety, and Pharmacodynamic Effects of the Bruton's Tyrosine Kinase Inhibitor Fenebrutinib (GDC-0853) in Systemic Lupus Erythematosus: Results of a Phase II, Randomized, Double-Blind, Placebo-Controlled Trial. Arthritis Rheumatol 73, 1835-1846.
34. Paulson, V. A., Shivdasani, P., Angell, T. E., Cibas, E. S., Krane, J. F., Lindeman, N. I., Alexander, E. K., and Barletta, J. A. (2017) Noninvasive Follicular Thyroid Neoplasm with Papillary-Like Nuclear Features Accounts for More Than Half of "Carcinomas" Harboring RAS Mutations. Thyroid 27, 506-511.

35. Chu, Y. H., Wirth, L. J., Farahani, A. A., Nose, V., Faquin, W. C., Dias-Santagata, D., and Sadow, P. M. (2020) Clinicopathologic features of kinase fusion-related thyroid carcinomas: an integrative analysis with molecular characterization. Mod Pathol 33, 2458-2472.
36. Snyder, A., Makarov, V., Merghoub, T., Yuan, J., Zaretsky, J. M., Desrichard, A., Walsh, L. A., Postow, M. A., Wong, P., Ho, T. S., et al. (2014) Genetic basis for clinical response to CTLA-4 blockade in melanoma. N Engl J Med 371, 2189-2199.
37. Curtin, J. A., Fridlyand, J., Kageshita, T., Patel, H. N., Busam, K. J., Kutzner, H., Cho, K. H., Aiba, S., Brocker, E. B., LeBoit, P. E., et al. (2005) Distinct sets of genetic alterations in melanoma. N Engl J Med 353, 2135-2147.
38. Richardson, P. G., Sonneveld, P., Schuster, M. W., Irwin, D., Stadtmauer, E. A., Facon, T., Harousseau, J. L., Ben-Yehuda, D., Lonial, S., Goldschmidt, H., et al. (2005) Bortezomib or high-dose dexamethasone for relapsed multiple myeloma. N Engl J Med 352, 2487-2498.
39. Hauser, S. L., Waubant, E., Arnold, D. L., Vollmer, T., Antel, J., Fox, R. J., Bar-Or, A., Panzara, M., Sarkar, N., Agarwal, S., et al. (2008) B-cell depletion with rituximab in relapsing-remitting multiple sclerosis. N Engl J Med 358, 676-688.
40. Kappos, L., Radue, E. W., O'Connor, P., Polman, C., Hohlfeld, R., Calabresi, P., Selmaj, K., Agoropoulou, C., Leyk, M., Zhang-Auberson, L., et al. (2010) A placebo-controlled trial of oral fingolimod in relapsing multiple sclerosis. N Engl J Med 362, 387-401.
41. Patel, J. P., Gonen, M., Figueroa, M. E., Fernandez, H., Sun, Z., Racevskis, J., Van Vlierberghe, P., Dolgalev, I., Thomas, S., Aminova, O., et al. (2012) Prognostic relevance of integrated genetic profiling in acute myeloid leukemia. N Engl J Med 366, 1079-1089.
42. Tabrizi, S. J., Leavitt, B. R., Landwehrmeyer, G. B., Wild, E. J., Saft, C., Barker, R. A., Blair, N. F., Craufurd, D., Priller, J., Rickards, H., et al. (2019) Targeting Huntingtin Expression in Patients with Huntington's Disease. N Engl J Med 380, 2307-2316.
43. Liddelow, S. A., Guttenplan, K. A., Clarke, L. E., Bennett, F. C., Bohlen, C. J., Schirmer, L., Bennett, M. L., Munch, A. E., Chung, W. S., Peterson, T. C., et al. (2017) Neurotoxic reactive astrocytes are induced by activated microglia. Nature 541, 481-487.
44. Gilhus, N. E. (2016) Myasthenia Gravis. N Engl J Med 375, 2570-2581.
45. Guglielmetti, C., Levi, J., Huynh, T. L., Tiret, B., Blecha, J., Tang, R., VanBrocklin, H., and Chaumeil, M. M. (2022) Longitudinal Imaging of T Cells and Inflammatory Demyelination in a Preclinical Model of Multiple Sclerosis Using (18)F-FAraG PET and MRI. J Nucl Med 63, 140-146.
46. Hauser, S. L., Zielman, R., Das Gupta, A., Xi, J., Stoneman, D., Karlsson, G., Robertson, D., Cohen, J. A., and Kappos, L. (2023) Efficacy and safety of four-year ofatumumab treatment in relapsing multiple sclerosis: The ALITHIOS open-label extension. Mult Scler 29, 1452-1464.
47. Rojo, D., Dal Cengio, L., Badner, A., Kim, S., Sakai, N., Greene, J., Dierckx, T., Mehl, L. C., Eisinger, E., Ransom, J., et al. (2023) BMAL1 loss in oligodendroglia contributes to abnormal myelination and sleep. Neuron 111, 3604-3618 e3611.
48. Baghdassarian, H., Blackstone, S. A., Clay, O. S., Philips, R., Matthiasardottir, B., Nehrebecky, M., Hua, V. K., McVicar, R., Liu, Y., Tucker, S. M., et al. (2023) Variant STAT4 and Response to Ruxolitinib in an Autoinflammatory Syndrome. N Engl J Med 388, 2241-2252.
49. Hasanov, E., and Jonasch, E. (2021) MK-6482 as a potential treatment for von Hippel-Lindau disease-associated clear cell renal cell carcinoma. Expert Opin Investig Drugs 30, 495-504.
50. Compositions and methods for preserving and/or restoring neural function. WO2023086603, 2023.
51. Ire1alpha inhibitors and uses thereof. WO2022104148, 2022.
52. Viral delivery of therapeutics to the central nervous system. WO2022165538A1, 2022.
53. Enhancing gaba's ability to modulate immune responses. WO2018236955, 2018.
54. Fairclough, R. H., and Trinh, V. B. Peptides and uses thereof for diagnosing and treating myasthenia gravis. WO2018049053, 2018.
55. Multiple sclerosis. https://www.mayoclinic.org/diseases-conditions/multiple-sclerosis/symptoms-causes/syc-20350269#:~:text=Multiple%20sclerosis%20(MS)%20is%20a,the%20rest%20of%20your%20body. (accessed 11th March).
56. Systemic Lupus Erythematosus (SLE). https://www.cdc.gov/lupus/facts/detailed.html (accessed 11th March).
57. Familial Mediterranean fever. https://www.mayoclinic.org/diseases-conditions/familial-mediterranean-fever/symptoms-causes/syc-20372470 (accessed 11th March).

58. Rett syndrome. https://www.mayoclinic.org/diseases-conditions/rett-syndrome/symptoms-causes/syc-20377227 (accessed 11th March).
59. Rizzolo, K., Beck, N. M., and Ambruso, S. L. (2022) Syndromes of Pseudo-Hyperaldosteronism. Clin J Am Soc Nephrol 17, 581-584.
60. Kidney cancer. https://www.mayoclinic.org/diseases-conditions/kidney-cancer/symptoms-causes/syc-20352664 (accessed 11th March).
61. Thyroid cancer. https://www.mayoclinic.org/diseases-conditions/thyroid-cancer/symptoms-causes/syc-20354161 (accessed 11th March).
62. Melanoma. https://www.mayoclinic.org/diseases-conditions/melanoma/symptoms-causes/syc-20374884#:~:text=deeper%20skin%20layers.-,Melanoma%20is%20a%20kind%20of%20skin%20cancer%20that%20starts%20in,often%20exposed%20to%20the%20sun. (accessed 11th March).
63. Multiple myeloma. https://www.mayoclinic.org/diseases-conditions/multiple-myeloma/symptoms-causes/syc-20353378#:~:text=Multiple%20myeloma%20is%20a%20cancer,build%20up%20in%20bone%20marrow. (accessed 11th March).
64. Nasopharyngeal carcinoma. https://www.mayoclinic.org/diseases-conditions/nasopharyngeal-carcinoma/symptoms-causes/syc-20375529 (accessed 11th March).
65. Cuglievan, B., Connors, J., He, J., Khazal, S., Yedururi, S., Dai, J., Garces, S., Quesada, A. E., Roth, M., Garcia, M., et al. (2023) Blastic plasmacytoid dendritic cell neoplasm: a comprehensive review in pediatrics, adolescents, and young adults (AYA) and an update of novel therapies. Leukemia 37, 1767-1778.
66. Freedman, A. S. A., J. C.; Dearden, C. B cell prolymphocytic leukemia. https://www.uptodate.com/contents/b-cell-prolymphocytic-leukemia#:~:text=B%20cell%20prolymphocytic%20leukemia%20(B,are%20mature%20activated%20B%20cells. (accessed 11th March).
67. Bindra, B. S., Kaur, H., Portillo, S., Emiloju, O., and Garcia de de Jesus, K. (2019) B-cell Prolymphocytic Leukemia: Case Report and Challenges on a Diagnostic and Therapeutic Forefront. Cureus 11, e5629.
68. Keon, M., Musrie, B., Dinger, M., Brennan, S. E., Santos, J., and Saksena, N. K. (2021) Destination Amyotrophic Lateral Sclerosis. Front Neurol 12, 596006.
69. Rojas, P., Ramírez, A. I., Fernández-Albarral, J. A., López-Cuenca, I., Salobrar-García, E., Cadena, M., Elvira-Hurtado, L., Salazar, J. J., de Hoz, R., and Ramírez, J. M. (2020) Amyotrophic Lateral Sclerosis: A Neurodegenerative Motor Neuron Disease With Ocular Involvement. Front Neurosci 14, 566858.
70. Amyotrophic lateral sclerosis (ALS). https://www.mayoclinic.org/diseases-conditions/amyotrophic-lateral-sclerosis/symptoms-causes/syc-20354022#:~:text=ALS%20often%20begins%20with%20muscle,cure%20for%20this%20fatal%20disease . (accessed Feb 26, 2024).
71. Ling, S. C., Polymenidou, M., and Cleveland, D. W. (2013) Converging mechanisms in ALS and FTD: disrupted RNA and protein homeostasis. Neuron 79, 416-438.
72. Lattante, S., Ciura, S., Rouleau, G. A., and Kabashi, E. (2015) Defining the genetic connection linking amyotrophic lateral sclerosis (ALS) with frontotemporal dementia (FTD). Trends Genet 31, 263-273.
73. Fecto, F., and Siddique, T. (2011) Making connections: pathology and genetics link amyotrophic lateral sclerosis with frontotemporal lobe dementia. J Mol Neurosci 45, 663-675.
74. Chiò, A., Borghero, G., Restagno, G., Mora, G., Drepper, C., Traynor, B. J., Sendtner, M., Brunetti, M., Ossola, I., Calvo, A., et al. (2012) Clinical characteristics of patients with familial amyotrophic lateral sclerosis carrying the pathogenic GGGGCC hexanucleotide repeat expansion of C9ORF72. Brain 135, 784-793.
75. Mejzini, R., Flynn, L. L., Pitout, I. L., Fletcher, S., Wilton, S. D., and Akkari, P. A. (2019) ALS Genetics, Mechanisms, and Therapeutics: Where Are We Now? Front Neurosci 13, 1310.
76. ALS Genes and Mutations. https://www.als.org/research/als-research-topics/genetics (accessed Feb 26, 2024).
77. Paganoni, S., Macklin, E. A., Lee, A., Murphy, A., Chang, J., Zipf, A., Cudkowicz, M., and Atassi, N. (2014) Diagnostic timelines and delays in diagnosing amyotrophic lateral sclerosis (ALS). Amyotroph Lateral Scler Frontotemporal Degener 15, 453-456.

78. Hardiman, O., van den Berg, L. H., and Kiernan, M. C. (2011) Clinical diagnosis and management of amyotrophic lateral sclerosis. Nat Rev Neurol 7, 639-649.
79. Majmudar, S., Wu, J., and Paganoni, S. (2014) Rehabilitation in amyotrophic lateral sclerosis: why it matters. Muscle Nerve 50, 4-13.
80. FDA-Approved Drugs for Treating ALS. https://www.als.org/navigating-als/living-with-als/fda-approved-drugs (accessed Mar 18, 2024).
81. Kaylor, A. Exploring FDA-Approved Drugs and Potential Treatment Options for ALS. https://pharmanewsintel.com/features/exploring-fda-approved-drugs-and-potential-treatment-options-for-als#:~:text=Covis%20Pharma's%20Rilutek%20(riluzole)%20was,has%20been%20available%20since%202003. (accessed Mar 18, 2024).
82. RILUTEK (riluzole) tablets, for oral use https://www.accessdata.fda.gov/drugsatfda_docs/label/2016/020599s017lbl.pdf (accessed Mar 18, 2024).
83. Riluzole. https://medlineplus.gov/druginfo/meds/a696013.html#:~:text=Riluzole%20is%20used%20to%20treat,that%20affect%20nerves%20and%20muscles. (accessed Mar 26, 2024).
84. FDA approves drug to treat ALS. https://www.fda.gov/news-events/press-announcements/fda-approves-drug-treat-als (accessed Mar 18, 2024).
85. RELYVRIO (sodium phenylbutyrate and taurursodiol), for oral suspension. https://www.accessdata.fda.gov/drugsatfda_docs/label/2022/216660s000lbledt.pdf (accessed Mar 18, 2024).
86. Sodium Phenylbutyrate and Taurursodiol. https://medlineplus.gov/druginfo/meds/a623014.html (accessed Mar 26, 2024).
87. McKenzie, H. Future of Amylyx's ALS Drug in Question After Phase III Failure. https://www.biospace.com/article/future-of-amylyx-s-als-drug-in-question-after-phase-iii-failure-/ (accessed Mar 26, 2024).
88. Bryson, S. MTPA to stop Phase 3 trial of approved Radicava ORS for ALS. https://alsnewstoday.com/news/mtpa-to-stop-phase-3-trial-of-approved-radicava-ors-for-als/ (accessed Mar 26, 2024).
89. Ferrer reports top-line results from Phase III ADORE study in ALS. https://www.ferrer.com/en/results-study-ADORE-ALS (accessed Mar 26, 2024).
90. Bali, T., and Miller, T. M. (2013) Management of amyotrophic lateral sclerosis. Mo Med 110, 417-421.
91. Phukan, J., and Hardiman, O. (2009) The management of amyotrophic lateral sclerosis. J Neurol 256, 176-186.
92. Brooks, B. R. (2009) Managing amyotrophic lateral sclerosis: Slowing disease progression and improving patient quality of life. Annals of Neurology 65, S17-S23.
93. Ferraiuolo, L., Kirby, J., Grierson, A. J., Sendtner, M., and Shaw, P. J. (2011) Molecular pathways of motor neuron injury in amyotrophic lateral sclerosis. Nat Rev Neurol 7, 616-630.
94. van den Bos, M. A. J., Geevasinga, N., Higashihara, M., Menon, P., and Vucic, S. (2019) Pathophysiology and Diagnosis of ALS: Insights from Advances in Neurophysiological Techniques. Int J Mol Sci 20, 2818.
95. Warner, T. T., Chapter 10 - Motor Neuron Diseases. In *Practical Guide to Neurogenetics*, Warner, T. T.; Hammans, S. R., Eds. W.B. Saunders: Philadelphia, 2009; pp 150-174.
96. Siddique, T., Deng, H. X., and Ajroud-Driss, S., Chapter 132 - Motor Neuron Disease. In *Emery and Rimoin's Principles and Practice of Medical Genetics (Sixth Edition)*, Rimoin, D.; Pyeritz, R.; Korf, B., Eds. Academic Press: Oxford, 2013; pp 1-22.
97. Ragagnin, A. M. G., Shadfar, S., Vidal, M., Jamali, M. S., and Atkin, J. D. (2019) Motor Neuron Susceptibility in ALS/FTD. Front Neurosci 13, 532.
98. Boillée, S., Vande Velde, C., and Cleveland, Don W. (2006) ALS: A Disease of Motor Neurons and Their Nonneuronal Neighbors. Neuron 52, 39-59.
99. Foran, E., and Trotti, D. (2009) Glutamate transporters and the excitotoxic path to motor neuron degeneration in amyotrophic lateral sclerosis. Antioxid Redox Signal 11, 1587-1602.
100. Jiang, L. L., Zhu, B., Zhao, Y., Li, X., Liu, T., Pina-Crespo, J., Zhou, L., Xu, W., Rodriguez, M. J., Yu, H., et al. (2019) Membralin deficiency dysregulates astrocytic glutamate homeostasis leading to ALS-like impairment. J Clin Invest 129, 3103-3120.

101. Xie, M., Pallegar, P. N., Parusel, S., Nguyen, A. T., and Wu, L.-J. (2023) Regulation of cortical hyperexcitability in amyotrophic lateral sclerosis: focusing on glial mechanisms. Molecular Neurodegeneration 18, 75.
102. Singh, A., Kukreti, R., Saso, L., and Kukreti, S. (2019) Oxidative Stress: A Key Modulator in Neurodegenerative Diseases. Molecules 24, 1583.
103. Liu, Z., Zhou, T., Ziegler, A. C., Dimitrion, P., and Zuo, L. (2017) Oxidative Stress in Neurodegenerative Diseases: From Molecular Mechanisms to Clinical Applications. Oxid Med Cell Longev 2017, 2525967.
104. Duranti, E., and Villa, C. (2022) Molecular Investigations of Protein Aggregation in the Pathogenesis of Amyotrophic Lateral Sclerosis. Int J Mol Sci 24, 704.
105. Blokhuis, A. M., Groen, E. J., Koppers, M., van den Berg, L. H., and Pasterkamp, R. J. (2013) Protein aggregation in amyotrophic lateral sclerosis. Acta Neuropathol 125, 777-794.
106. Malik, R., and Wiedau, M. (2020) Therapeutic Approaches Targeting Protein Aggregation in Amyotrophic Lateral Sclerosis. Frontiers in Molecular Neuroscience 13, 98.
107. Cicardi, M. E., Marrone, L., Azzouz, M., and Trotti, D. (2021) Proteostatic imbalance and protein spreading in amyotrophic lateral sclerosis. The EMBO Journal 40, e106389.
108. Zhao, J., Wang, X., Huo, Z., Chen, Y., Liu, J., Zhao, Z., Meng, F., Su, Q., Bao, W., Zhang, L., et al. (2022) The Impact of Mitochondrial Dysfunction in Amyotrophic Lateral Sclerosis. Cells 11, 2049.
109. Muyderman, H., and Chen, T. (2014) Mitochondrial dysfunction in amyotrophic lateral sclerosis - a valid pharmacological target? Br J Pharmacol 171, 2191-2205.
110. Smith, E. F., Shaw, P. J., and De Vos, K. J. (2019) The role of mitochondria in amyotrophic lateral sclerosis. Neuroscience Letters 710, 132933.
111. Provenzano, F., Torazza, C., Bonifacino, T., Bonanno, G., and Milanese, M. (2023) The Key Role of Astrocytes in Amyotrophic Lateral Sclerosis and Their Commitment to Glutamate Excitotoxicity. International Journal of Molecular Sciences 24, 15430.
112. Lee, J., Hyeon, S. J., Im, H., Ryu, H., Kim, Y., and Ryu, H. (2016) Astrocytes and Microglia as Non-cell Autonomous Players in the Pathogenesis of ALS. Exp Neurobiol 25, 233-240.
113. You, J., Youssef, M. M. M., Santos, J. R., Lee, J., and Park, J. (2023) Microglia and Astrocytes in Amyotrophic Lateral Sclerosis: Disease-Associated States, Pathological Roles, and Therapeutic Potential. Biology (Basel) 12, 1307.
114. Van Harten, A. C. M., Phatnani, H., and Przedborski, S. (2021) Non-cell-autonomous pathogenic mechanisms in amyotrophic lateral sclerosis. Trends in Neurosciences 44, 658-668.
115. Hartzfeld, D. FYI: Familial Amyotrophic Lateral Sclerosis (FALS) and Genetic Testing. https://www.als.org/navigating-als/resources/familial-amyotrophic-lateral-sclerosis-fals-and-genetic#:~:text=About%2010%25%20of%20cases%20are,is%20most%20often%20autosomal%20dominant. (accessed Feb 26, 2024).
116. Siddique, T., and Ajroud-Driss, S. (2011) Familial amyotrophic lateral sclerosis, a historical perspective. Acta Myol 30, 117-120.
117. Oskarsson, B., Horton, D. K., and Mitsumoto, H. (2015) Potential Environmental Factors in Amyotrophic Lateral Sclerosis. Neurol Clin 33, 877-888.
118. Bozzoni, V., Pansarasa, O., Diamanti, L., Nosari, G., Cereda, C., and Ceroni, M. (2016) Amyotrophic lateral sclerosis and environmental factors. Funct Neurol 31, 7-19.
119. Andrew, A. S., Bradley, W. G., Peipert, D., Butt, T., Amoako, K., Pioro, E. P., Tandan, R., Novak, J., Quick, A., Pugar, K. D., et al. (2021) Risk factors for amyotrophic lateral sclerosis: A regional United States case-control study. Muscle & Nerve 63, 52-59.
120. Rosenfeld, J., and Strong, M. J. (2015) Challenges in the Understanding and Treatment of Amyotrophic Lateral Sclerosis/Motor Neuron Disease. Neurotherapeutics 12, 317-325.
121. Zipprich, B. ALS Is Not a Singular Disease. Stop Treating It Like One. https://www.biospace.com/article/opinion-als-is-not-a-singular-disease-stop-treating-it-like-one/ (accessed Mar 26, 2024).
122. Sari, Y. (2011) Huntington's Disease: From Mutant Huntingtin Protein to Neurotrophic Factor Therapy. Int J Biomed Sci 7, 89-100.
123. Bates, G. P. (2005) History of genetic disease: the molecular genetics of Huntington disease - a history. Nat Rev Genet 6, 766-773.

124. Imarisio, S., Carmichael, J., Korolchuk, V., Chen, C. W., Saiki, S., Rose, C., Krishna, G., Davies, J. E., Ttofi, E., Underwood, B. R., and Rubinsztein, D. C. (2008) Huntington's disease: from pathology and genetics to potential therapies. Biochem J 412, 191-209.
125. Sturrock, A., and Leavitt, B. R. (2010) The clinical and genetic features of Huntington disease. J Geriatr Psychiatry Neurol 23, 243-259.
126. Groves, M., Vonsattel, J.-P., Mazzoni, P., and Marder, K. (2003) Huntington's Disease. Science of Aging Knowledge Environment 2003, dn3-dn3.
127. Mahalingam, S., and Levy, L. M. (2014) Genetics of Huntington Disease. American Journal of Neuroradiology 35, 1070-1072.
128. Jurcau, A. (2022) Molecular Pathophysiological Mechanisms in Huntington's Disease. Biomedicines 10, 1432.
129. Barron, J. C., Hurley, E. P., and Parsons, M. P. (2021) Huntingtin and the Synapse. Frontiers in Cellular Neuroscience 15, 689332.
130. Huntington's Disease. https://www.ninds.nih.gov/health-information/disorders/huntingtons-disease#:~:text=Early%20signs%20of%20HD%20can,increases%20the%20chances%20of%20falling. (accessed Feb 26, 2024).
131. Roos, R. A. C. (2010) Huntington's disease: a clinical review. Orphanet Journal of Rare Diseases 5, 40.
132. Tampi, R. R., and Weber, M. Early Warnings: Neuropsychiatric Manifestations of Huntington Disease. https://www.psychiatrictimes.com/view/early-warnings-neuropsychiatric-manifestations-huntington-disease (accessed Feb 26, 2024).
133. Paulsen, J. S. (2011) Cognitive impairment in Huntington disease: diagnosis and treatment. Curr Neurol Neurosci Rep 11, 474-483.
134. Friedman, N. P., and Robbins, T. W. (2022) The role of prefrontal cortex in cognitive control and executive function. Neuropsychopharmacology 47, 72-89.
135. McAllister, B., Gusella, J. F., Landwehrmeyer, G. B., Lee, J.-M., MacDonald, M. E., Orth, M., Rosser, A. E., Williams, N. M., Holmans, P., Jones, L., et al. (2021) Timing and Impact of Psychiatric, Cognitive, and Motor Abnormalities in Huntington Disease. Neurology 96, e2395-e2406.
136. Peixoto, C., Rego, D., Bicho, M., Coelho, J., and Medeiros, H., *Psychiatric symptoms in huntington's disease*. Eur Psychiatry. 2021 Aug 13;64(Suppl 1):S254-5. doi: 10.1192/j.eurpsy.2021.682. eCollection 2021 Apr.
137. Parsons, M. P., and Raymond, L. A., Chapter 20 - Huntington Disease. In *Neurobiology of Brain Disorders*, Zigmond, M. J.; Rowland, L. P.; Coyle, J. T., Eds. Academic Press: San Diego, 2015; pp 303-320.
138. Douglas, I., Evans, S., Rawlins, M. D., Smeeth, L., Tabrizi, S. J., and Wexler, N. S. (2013) Juvenile Huntington's disease: a population-based study using the General Practice Research Database. BMJ Open 3, 002085.
139. Yero, T., and Rey, J. A. (2008) Tetrabenazine (Xenazine), An FDA-Approved Treatment Option For Huntington's Disease-Related Chorea. P t 33, 690-694.
140. AUSTEDO™ (deutetrabenazine) tablets, for oral use. https://www.accessdata.fda.gov/drugsatfda_docs/label/2017/208082s000lbl.pdf (accessed Mar 18, 2024).
141. TEVA announces FDA approval of AUSTEDO® XR (Deutetrabenazine) extended-release tablets, a new once-daily formulation of AUSTEDO® (Deutetrabenazine) tablets. https://hdsa.org/news/eva-announces-fda-approval-of-austedo-xr-deutetrabenazine-extended-release-tablets-a-new-once-daily-formulation-of-austedo-deutetrabenazine-tablets/ (accessed Mar 18, 2024).
142. Dhingra, H., and Gaidhane, S. A. (2023) Huntington's Disease: Understanding Its Novel Drugs and Treatments. Cureus 15, e47526.
143. Kim, A., Lalonde, K., Truesdell, A., Gomes Welter, P., Brocardo, P. S., Rosenstock, T. R., and Gil-Mohapel, J. (2021) New Avenues for the Treatment of Huntington's Disease. International journal of molecular sciences 22, 8363.
144. Muthane, U. (2011) Predictive genetic testing in Huntington's disease. Ann Indian Acad Neurol 14, S29-30.
145. Andrew, K. M., and Fox, L. M. (2023) Supporting Huntington's Disease Families Through the Ups and Downs of Clinical Trials. J Huntingtons Dis 12, 71-76.
146. Current HSG Clinical Trials & Studies. https://huntingtonstudygroup.org/current-clinical-trials/ (accessed Fer 26, 2024).

147. Rocha, N. P., Colpo, G. D., Teixeira, A. L., and Stimming, E. F. Clinical Trials for Huntington Disease. https://practicalneurology.com/articles/2020-june/clinical-trials-for-huntington-disease (accessed Feb 26, 2024).
148. Jones, L., and Hughes, A. (2011) Pathogenic mechanisms in Huntington's disease. Int Rev Neurobiol 98, 373-418.
149. Irfan, Z., Khanam, S., Karmakar, V., Firdous, S. M., El Khier, B., Khan, I., Rehman, M. U., and Khan, A. (2022) Pathogenesis of Huntington's Disease: An Emphasis on Molecular Pathways and Prevention by Natural Remedies. Brain Sci 12, 1389.
150. Jimenez-Sanchez, M., Licitra, F., Underwood, B. R., and Rubinsztein, D. C. (2017) Huntington's Disease: Mechanisms of Pathogenesis and Therapeutic Strategies. Cold Spring Harb Perspect Med 7, a024240.
151. The Huntington Gene. https://health.ucdavis.edu/huntingtons/genetic-change.html (accessed Feb 26, 2024).
152. Daldin, M., Fodale, V., Cariulo, C., Azzollini, L., Verani, M., Martufi, P., Spiezia, M. C., Deguire, S. M., Cherubini, M., Macdonald, D., et al. (2017) Polyglutamine expansion affects huntingtin conformation in multiple Huntington's disease models. Sci Rep 7, 5070.
153. Finkbeiner, S. (2011) Huntington's Disease. Cold Spring Harb Perspect Biol 3, a007476.
154. Kuang, X., Nunn, K., Jiang, J., Castellano, P., Hardikar, U., Horgan, A., Kong, J., Tan, Z., and Dai, W. (2021) Structural insight into transmissive mutant huntingtin species by correlative light and electron microscopy and cryo-electron tomography. Biochemical and Biophysical Research Communications 560, 99-104.
155. Johri, A., and Beal, M. F. (2012) Mitochondrial dysfunction in neurodegenerative diseases. J Pharmacol Exp Ther 342, 619-630.
156. Kamitsuka, P. J., Ghanem, M. M., Ziar, R., McDonald, S. E., Thomas, M. G., and Kwakye, G. F. (2023) Defective Mitochondrial Dynamics and Protein Degradation Pathways Underlie Cadmium-Induced Neurotoxicity and Cell Death in Huntington's Disease Striatal Cells. Int J Mol Sci 24, 7178.
157. Dai, Y., Wang, H., Lian, A., Li, J., Zhao, G., Hu, S., and Li, B. (2023) A comprehensive perspective of Huntington’s disease and mitochondrial dysfunction. Mitochondrion 70, 8-19.
158. Miladinovic, T., Nashed, M. G., and Singh, G. (2015) Overview of Glutamatergic Dysregulation in Central Pathologies. Biomolecules 5, 3112-3141.
159. Henningsen, J. B., Soylu-Kucharz, R., Björkqvist, M., and Petersén, Å. (2021) Effects of excitotoxicity in the hypothalamus in transgenic mouse models of Huntington disease. Heliyon 7, e07808.
160. Anglada-Huguet, M., Laura, V.-S., Nuria, C.-L., Jordi, A., and Xavier, X., Pathogenesis of Huntington’s Disease: How to Fight Excitotoxicity and Transcriptional Dysregulation. In *Huntington's Disease*, Nagehan Ersoy, T., Ed. IntechOpen: Rijeka, 2017; pp 37-73.
161. Lewerenz, J., and Maher, P. (2015) Chronic Glutamate Toxicity in Neurodegenerative Diseases—What is the Evidence? Frontiers in Neuroscience 9, 00469.
162. Trushina, E., Dyer, R. B., Badger, J. D., 2nd, Ure, D., Eide, L., Tran, D. D., Vrieze, B. T., Legendre-Guillemin, V., McPherson, P. S., Mandavilli, B. S., et al. (2004) Mutant huntingtin impairs axonal trafficking in mammalian neurons in vivo and in vitro. Mol Cell Biol 24, 8195-8209.
163. Berth, S. H., and Lloyd, T. E. (2023) Disruption of axonal transport in neurodegeneration. J Clin Invest 133, e168554.
164. Migazzi, A., Scaramuzzino, C., Anderson, E. N., Tripathy, D., Hernández, I. H., Grant, R. A., Roccuzzo, M., Tosatto, L., Virlogeux, A., Zuccato, C., et al. (2021) Huntingtin-mediated axonal transport requires arginine methylation by PRMT6. Cell Reports 35, 108980.
165. Jia, Q., Li, S., Li, X. J., and Yin, P. (2022) Neuroinflammation in Huntington's disease: From animal models to clinical therapeutics. Front Immunol 13, 1088124.
166. Rocha, N. P., Ribeiro, F. M., Furr-Stimming, E., and Teixeira, A. L. (2016) Neuroimmunology of Huntington's Disease: Revisiting Evidence from Human Studies. Mediators Inflamm 2016, 8653132.
167. Zhang, W., Xiao, D., Mao, Q., and Xia, H. (2023) Role of neuroinflammation in neurodegeneration development. Signal Transduction and Targeted Therapy 8, 267.
168. Kumar, A., Vaish, M., and Ratan, R. R. (2014) Transcriptional dysregulation in Huntington's disease: a failure of adaptive transcriptional homeostasis. Drug Discov Today 19, 956-962.
169. Pradhan, S., Gao, R., Bush, K., Zhang, N., Wairkar, Y. P., and Sarkar, P. S. (2022) Polyglutamine Expansion in Huntingtin and Mechanism of DNA Damage Repair Defects in Huntington's Disease. Front Cell Neurosci 16, 837576.

170. Hervás-Corpión, I., Guiretti, D., Alcaraz-Iborra, M., Olivares, R., Campos-Caro, A., Barco, Á., and Valor, L. M. (2018) Early alteration of epigenetic-related transcription in Huntington's disease mouse models. Scientific Reports 8, 9925.
171. Smith-Dijak, A. I., Sepers, M. D., and Raymond, L. A. (2019) Alterations in synaptic function and plasticity in Huntington disease. J Neurochem 150, 346-365.
172. Cepeda, C., and Levine, M. S. (2022) Synaptic Dysfunction in Huntington's Disease: Lessons from Genetic Animal Models. Neuroscientist 28, 20-40.
173. Morigaki, R., and Goto, S. (2017) Striatal Vulnerability in Huntington's Disease: Neuroprotection Versus Neurotoxicity. Brain Sci 7, 63.
174. Han, I., You, Y., Kordower, J. H., Brady, S. T., and Morfini, G. A. (2010) Differential vulnerability of neurons in Huntington's disease: the role of cell type-specific features. J Neurochem 113, 1073-1091.
175. Mätlik, K., Baffuto, M., Kus, L., Deshmukh, A. L., Davis, D. A., Paul, M. R., Carroll, T. S., Caron, M.-C., Masson, J.-Y., Pearson, C. E., and Heintz, N. (2024) Cell-type-specific CAG repeat expansions and toxicity of mutant Huntingtin in human striatum and cerebellum. Nature Genetics 56, 383-394.
176. Koneczny, I., Martinez, P. M., and De Baets, M., Myasthenia Gravis. In *Encyclopedia of Immunobiology*, Ratcliffe, M. J. H., Ed. Academic Press: Oxford, 2016; pp 168-179.
177. Verschuuren, J., Strijbos, E., and Vincent, A., Chapter 24 - Neuromuscular junction disorders. In *Handbook of Clinical Neurology*, Pittock, S. J.; Vincent, A., Eds. Elsevier: 2016; Vol. 133, pp 447-466.
178. Szilagyi, E., Sundivakkam, P., Nunez, T., Premenand, K., Kenyon, N., and Bartholomew, A., Chapter 37 - Clinical Aspects of Regenerative Medicine: Immune System. In *Translational Regenerative Medicine*, Atala, A.; Allickson, J. G., Eds. Academic Press: Boston, 2015; pp 507-526.
179. Pal, J., Rozsa, C., Komoly, S., and Illes, Z. (2011) Clinical and biological heterogeneity of autoimmune myasthenia gravis. J Neuroimmunol 231, 43-54.
180. Nguyen, A. Myasthenia Gravis: Causes, Risk Factors, Diagnosis, Treatment and Current/Future Research. https://app.scientist.com/blog/2023/06/08/myasthenia-gravis-causes-risk-factors-diagnosis-treatment-and-current-future-research (accessed Feb 26, 2024).
181. Shah, A. K. Myasthenia Gravis Medication. https://emedicine.medscape.com/article/1171206-medication?form=fpf&scode=msp&st=fpf&socialSite=google&icd=login_success_gg_match_fpf (accessed Feb 26, 2024).
182. Farmakidis, C., Pasnoor, M., Dimachkie, M. M., and Barohn, R. J. (2018) Treatment of Myasthenia Gravis. Neurol Clin 36, 311-337.
183. Alhaidar, M. K., Abumurad, S., Soliven, B., and Rezania, K. (2022) Current Treatment of Myasthenia Gravis. Journal of clinical medicine 11, 1597.
184. Aydin, Y., Ulas, A. B., Mutlu, V., Colak, A., and Eroglu, A. (2017) Thymectomy in Myasthenia Gravis. Eurasian J Med 49, 48-52.
185. Mantegazza, R., and Cavalcante, P. (2019) Diagnosis and treatment of myasthenia gravis. Curr Opin Rheumatol 31, 623-633.
186. Bird, S. J. Overview of the treatment of myasthenia gravis. https://www.uptodate.com/contents/overview-of-the-treatment-of-myasthenia-gravis (accessed Feb 26, 2024).
187. Hehir, M. K., 2nd, and Li, Y. (2022) Diagnosis and Management of Myasthenia Gravis. Continuum (Minneap Minn) 28, 1615-1642.
188. Angelini, C. (2011) Diagnosis and management of autoimmune myasthenia gravis. Clin Drug Investig 31, 1-14.
189. Carr, A. S., Cardwell, C. R., McCarron, P. O., and McConville, J. (2010) A systematic review of population based epidemiological studies in Myasthenia Gravis. BMC Neurol 10, 46.
190. Mao, Z. F., Mo, X. A., Qin, C., Lai, Y. R., and Olde Hartman, T. C. (2010) Course and prognosis of myasthenia gravis: a systematic review. Eur J Neurol 17, 913-921.
191. Sieb, J. P. (2014) Myasthenia gravis: an update for the clinician. Clin Exp Immunol 175, 408-418.
192. Phillips, W. D., and Vincent, A. (2016) Pathogenesis of myasthenia gravis: update on disease types, models, and mechanisms. F1000Res 5.
193. Dresser, L., Wlodarski, R., Rezania, K., and Soliven, B. (2021) Myasthenia Gravis: Epidemiology, Pathophysiology and Clinical Manifestations. J Clin Med 10, 2235.
194. Meriggioli, M. N., and Sanders, D. B. (2009) Autoimmune myasthenia gravis: emerging clinical and biological heterogeneity. Lancet Neurol 8, 475-490.

195. Berrih-Aknin, S., and Le Panse, R. (2014) Myasthenia gravis: a comprehensive review of immune dysregulation and etiological mechanisms. J Autoimmun 52, 90-100.
196. Ha, J. C., and Richman, D. P. (2015) Myasthenia gravis and related disorders: Pathology and molecular pathogenesis. Biochim Biophys Acta 1852, 651-657.
197. Lazaridis, K., and Tzartos, S. J. (2020) Autoantibody Specificities in Myasthenia Gravis; Implications for Improved Diagnostics and Therapeutics. Frontiers in Immunology 11, 00212.
198. Gilhus, N. E., Tzartos, S., Evoli, A., Palace, J., Burns, T. M., and Verschuuren, J. J. G. M. (2019) Myasthenia gravis. Nature Reviews Disease Primers 5, 30.
199. Fichtner, M. L., Jiang, R., Bourke, A., Nowak, R. J., and O'Connor, K. C. (2020) Autoimmune Pathology in Myasthenia Gravis Disease Subtypes Is Governed by Divergent Mechanisms of Immunopathology. Frontiers in Immunology 11, 00776.
200. Wang, Z., and Yan, Y. (2017) Immunopathogenesis in Myasthenia Gravis and Neuromyelitis Optica. Front Immunol 8, 1785.
201. Serra, A., Ruff, R. L., and Leigh, R. J. (2012) Neuromuscular transmission failure in myasthenia gravis: decrement of safety factor and susceptibility of extraocular muscles. Ann N Y Acad Sci 1275, 129-135.
202. Ruff, R. L., and Lennon, V. A. (2008) How myasthenia gravis alters the safety factor for neuromuscular transmission. J Neuroimmunol 201-202, 13-20.
203. Howard Jr., J. F. (2018) Myasthenia gravis: the role of complement at the neuromuscular junction. Annals of the New York Academy of Sciences 1412, 113-128.
204. Tireli, H., Yuksel, G., Okay, T., and Tutkavul, K. (2020) Role of thymus on prognosis of myasthenia gravis in Turkish population. North Clin Istanb 7, 452-459.
205. Sanders, D. B., and Massey, J. M., Chapter 7 Clinical features of myasthenia gravis. In *Handbook of Clinical Neurology*, Elsevier: 2008; Vol. 91, pp 229-252.
206. Abbas, M. Decoding the Thymus Gland: Unveiling Its Role in Immune Function and Myasthenia Gravis. https://bnnbreaking.com/breaking-news/health/decoding-the-thymus-gland-unveiling-its-role-in-immune-function-and-myasthenia-gravis (accessed Feb 26, 2024).
207. Feigin, A., Evans, E. E., Fisher, T. L., Leonard, J. E., Smith, E. S., Reader, A., Mishra, V., Manber, R., Walters, K. A., Kowarski, L., et al. (2022) Pepinemab antibody blockade of SEMA4D in early Huntington's disease: a randomized, placebo-controlled, phase 2 trial. Nature Medicine 28, 2183-2193.
208. Vaccinex Pipeline. https://www.vaccinex.com/pipeline/ (accessed Apr 4, 2024).
209. Cytokinetics Pipeline. https://cytokinetics.com/medicines-research/pipeline/ (accessed Apr 4, 2024).
210. Sangamo: Neurology Conference Presentations Hub. https://www.sangamo.com/neurology-conference-presentations-hub/ (accessed Apr 4, 2024).
211. SOD1 superoxide dismutase 1 [Homo sapiens (human)]. https://www.ncbi.nlm.nih.gov/gene/6647 (accessed Apr 6, 2024).
212. Tainer, J. A., Getzoff, E. D., Richardson, J. S., and Richardson, D. C. (1983) Structure and mechanism of copper, zinc superoxide dismutase. Nature 306, 284-287.
213. Perry, J. J. P., Shin, D. S., Getzoff, E. D., and Tainer, J. A. (2010) The structural biochemistry of the superoxide dismutases. Biochimica et Biophysica Acta (BBA) - Proteins and Proteomics 1804, 245-262.
214. Chidambaram, S. B., Anand, N., Varma, S. R., Ramamurthy, S., Vichitra, C., Sharma, A., Mahalakshmi, A. M., and Essa, M. M. (2024) Superoxide dismutase and neurological disorders. IBRO Neuroscience Reports 16, 373-394.
215. Bruijn, L. I., Houseweart, M. K., Kato, S., Anderson, K. L., Anderson, S. D., Ohama, E., Reaume, A. G., Scott, R. W., and Cleveland, D. W. (1998) Aggregation and Motor Neuron Toxicity of an ALS-Linked SOD1 Mutant Independent from Wild-Type SOD1. Science (New York, N.Y.) 281, 1851-1854.
216. Sau, D., De Biasi, S., Vitellaro-Zuccarello, L., Riso, P., Guarnieri, S., Porrini, M., Simeoni, S., Crippa, V., Onesto, E., Palazzolo, I., et al. (2007) Mutation of SOD1 in ALS: a gain of a loss of function. Human Molecular Genetics 16, 1604-1618.
217. Alemasov, N. A., Ivanisenko, N. V., Ramachandran, S., and Ivanisenko, V. A. (2018) Molecular mechanisms underlying the impact of mutations in SOD1 on its conformational properties associated with amyotrophic lateral sclerosis as revealed with molecular modelling. BMC Structural Biology 18, 1.
218. Keskin, I., Forsgren, E., Lehmann, M., Andersen, P. M., Brännström, T., Lange, D. J., Synofzik, M., Nordström, U., Zetterström, P., Marklund, S. L., and Gilthorpe, J. D. (2019) The molecular pathogenesis of

superoxide dismutase 1-linked ALS is promoted by low oxygen tension. Acta Neuropathologica 138, 85-101.
219. Baek, Y., Woo, T.-G., Ahn, J., Lee, D., Kwon, Y., Park, B.-J., and Ha, N.-C. (2022) Structural analysis of the overoxidized Cu/Zn-superoxide dismutase in ROS-induced ALS filament formation. Communications Biology 5, 1085.
220. Berdyński, M., Miszta, P., Safranow, K., Andersen, P. M., Morita, M., Filipek, S., Żekanowski, C., and Kuźma-Kozakiewicz, M. (2022) SOD1 mutations associated with amyotrophic lateral sclerosis analysis of variant severity. Scientific Reports 12, 103.
221. SETX senataxin [Homo sapiens (human)]. https://www.ncbi.nlm.nih.gov/gene/23064 (accessed Apr 6, 2024).
222. Hasanova, Z., Klapstova, V., Porrua, O., Stefl, R., and Sebesta, M. (2023) Human senataxin is a bona fide R-loop resolving enzyme and transcription termination factor. Nucleic Acids Res 51, 2818-2837.
223. Moreira, M.-C., Klur, S., Watanabe, M., Németh, A. H., Ber, I. L., Moniz, J.-C., Tranchant, C., Aubourg, P., Tazir, M., Schöls, L., et al. (2004) Senataxin, the ortholog of a yeast RNA helicase, is mutant in ataxia-ocular apraxia 2. Nature Genetics 36, 225-227.
224. Asaka, T., Yokoji, H., Ito, J., Yamaguchi, K., and Matsushima, A. (2006) Autosomal recessive ataxia with peripheral neuropathy and elevated AFP: Novel mutations in <i>SETX</i>. Neurology 66, 1580-1581.
225. Datta, N., and Hohler, A. (2013) A new SETX mutation producing AOA2 in two siblings. International Journal of Neuroscience 123, 670-673.
226. Nanetti, L., Cavalieri, S., Pensato, V., Erbetta, A., Pareyson, D., Panzeri, M., Zorzi, G., Antozzi, C., Moroni, I., Gellera, C., et al. (2013) SETX mutations are a frequent genetic cause of juvenile and adult onset cerebellar ataxia with neuropathy and elevated serum alpha-fetoprotein. Orphanet Journal of Rare Diseases 8, 123.
227. AMYOTROPHIC LATERAL SCLEROSIS 4, JUVENILE; ALS4. https://www.omim.org/entry/602433 (accessed Apr 6, 2024).
228. Chen, Y.-Z., Bennett, C. L., Huynh, H. M., Blair, I. P., Puls, I., Irobi, J., Dierick, I., Abel, A., Kennerson, M. L., Rabin, B. A., et al. (2004) DNA/RNA Helicase Gene Mutations in a Form of Juvenile Amyotrophic Lateral Sclerosis (ALS4). The American Journal of Human Genetics 74, 1128-1135.
229. Bennett, C. L., Chen, Y., Vignali, M., Lo, R. S., Mason, A. G., Unal, A., Huq Saifee, N. P., Fields, S., and La Spada, A. R. (2013) Protein Interaction Analysis of Senataxin and the ALS4 L389S Mutant Yields Insights into Senataxin Post-Translational Modification and Uncovers Mutant-Specific Binding with a Brain Cytoplasmic RNA-Encoded Peptide. PloS one 8, e78837.
230. Tsui, A., Kouznetsova, V. L., Kesari, S., Fiala, M., and Tsigelny, I. F. (2023) Role of Senataxin in Amyotrophic Lateral Sclerosis. Journal of Molecular Neuroscience 73, 996-1009.
231. Giannini, M., and Porrua, O. (2024) Senataxin: A key actor in RNA metabolism, genome integrity and neurodegeneration. Biochimie 217, 10-19.
232. TARDBP TAR DNA binding protein [Homo sapiens (human)]. https://www.ncbi.nlm.nih.gov/gene/23435 (accessed Apr 6, 2024).
233. Jo, M., Lee, S., Jeon, Y.-M., Kim, S., Kwon, Y., and Kim, H.-J. (2020) The role of TDP-43 propagation in neurodegenerative diseases: integrating insights from clinical and experimental studies. Experimental & Molecular Medicine 52, 1652-1662.
234. François-Moutal, L., Perez-Miller, S., Scott, D. D., Miranda, V. G., Mollasalehi, N., and Khanna, M. (2019) Structural Insights Into TDP-43 and Effects of Post-translational Modifications. Frontiers in Molecular Neuroscience 12.
235. Boer, E. M. J. d., Orie, V. K., Williams, T., Baker, M. R., Oliveira, H. M. D., Polvikoski, T., Silsby, M., Menon, P., Bos, M. v. d., Halliday, G. M., et al. (2021) TDP-43 proteinopathies: a new wave of neurodegenerative diseases. Journal of Neurology, Neurosurgery & Psychiatry 92, 86-95.
236. Zhang, N., Gu, D., Meng, M., and Gordon, M. L. (2020) TDP-43 Is Elevated in Plasma Neuronal-Derived Exosomes of Patients With Alzheimer’s Disease. Frontiers in Aging Neuroscience 12.
237. Shih, Y.-H., Tu, L.-H., Chang, T.-Y., Ganesan, K., Chang, W.-W., Chang, P.-S., Fang, Y.-S., Lin, Y.-T., Jin, L.-W., and Chen, Y.-R. (2020) TDP-43 interacts with amyloid-β, inhibits fibrillization, and worsens pathology in a model of Alzheimer’s disease. Nature Communications 11, 5950.
238. Huang, W., Zhou, Y., Tu, L., Ba, Z., Huang, J., Huang, N., and Luo, Y. (2020) TDP-43: From Alzheimer’s Disease to Limbic-Predominant Age-Related TDP-43 Encephalopathy. Frontiers in Molecular Neuroscience 13.

239. Meneses, A., Koga, S., O'Leary, J., Dickson, D. W., Bu, G., and Zhao, N. (2021) TDP-43 Pathology in Alzheimer's Disease. Molecular Neurodegeneration 16, 84.
240. Tiloca, C., Goldwurm, S., Calcagno, N., Verde, F., Peverelli, S., Calini, D., Zecchinelli, A. L., Sangalli, D., Ratti, A., Pezzoli, G., et al. (2022) TARDBP mutations in a cohort of Italian patients with Parkinson's disease and atypical parkinsonisms. Frontiers in Aging Neuroscience 14.
241. Neumann, M., Sampathu, D. M., Kwong, L. K., Truax, A. C., Micsenyi, M. C., Chou, T. T., Bruce, J., Schuck, T., Grossman, M., Clark, C. M., et al. (2006) Ubiquitinated TDP-43 in Frontotemporal Lobar Degeneration and Amyotrophic Lateral Sclerosis. Science (New York, N.Y.) 314, 130-133.
242. Hu, W. T., and Grossman, M. (2009) TDP-43 and frontotemporal dementia. Current Neurology and Neuroscience Reports 9, 353-358.
243. Katisko, K., Huber, N., Kokkola, T., Hartikainen, P., Krüger, J., Heikkinen, A.-L., Paananen, V., Leinonen, V., Korhonen, V. E., Helisalmi, S., et al. (2022) Serum total TDP-43 levels are decreased in frontotemporal dementia patients with C9orf72 repeat expansion or concomitant motoneuron disease phenotype. Alzheimer's Research & Therapy 14, 151.
244. Van Deerlin, V. M., Leverenz, J. B., Bekris, L. M., Bird, T. D., Yuan, W., Elman, L. B., Clay, D., Wood, E. M., Chen-Plotkin, A. S., Martinez-Lage, M., et al. (2008) TARDBP mutations in amyotrophic lateral sclerosis with TDP-43 neuropathology: a genetic and histopathological analysis. The Lancet Neurology 7, 409-416.
245. Pesiridis, G. S., Lee, V. M.-Y., and Trojanowski, J. Q. (2009) Mutations in TDP-43 link glycine-rich domain functions to amyotrophic lateral sclerosis. Human Molecular Genetics 18, R156-R162.
246. Sreedharan, J., Blair, I. P., Tripathi, V. B., Hu, X., Vance, C., Rogelj, B., Ackerley, S., Durnall, J. C., Williams, K. L., Buratti, E., et al. (2008) TDP-43 Mutations in Familial and Sporadic Amyotrophic Lateral Sclerosis. Science (New York, N.Y.) 319, 1668-1672.
247. Prasad, A., Bharathi, V., Sivalingam, V., Girdhar, A., and Patel, B. K. (2019) Molecular Mechanisms of TDP-43 Misfolding and Pathology in Amyotrophic Lateral Sclerosis. Frontiers in Molecular Neuroscience 12.
248. Nilaver, B. I., and Urbanski, H. F. (2023) Mechanisms underlying TDP-43 pathology and neurodegeneration: An updated Mini-Review. Frontiers in Aging Neuroscience 15.
249. Arseni, D., Hasegawa, M., Murzin, A. G., Kametani, F., Arai, M., Yoshida, M., and Ryskeldi-Falcon, B. (2022) Structure of pathological TDP-43 filaments from ALS with FTLD. Nature 601, 139-143.
250. Arseni, D., Chen, R., Murzin, A. G., Peak-Chew, S. Y., Garringer, H. J., Newell, K. L., Kametani, F., Robinson, A. C., Vidal, R., Ghetti, B., et al. (2023) TDP-43 forms amyloid filaments with a distinct fold in type A FTLD-TDP. Nature 620, 898-903.
251. Oiwa, K., Watanabe, S., Onodera, K., Iguchi, Y., Kinoshita, Y., Komine, O., Sobue, A., Okada, Y., Katsuno, M., and Yamanaka, K. (2023) Monomerization of TDP-43 is a key determinant for inducing TDP-43 pathology in amyotrophic lateral sclerosis. Science Advances 9, eadf6895.
252. OPTN optineurin [Homo sapiens (human)]. https://www.ncbi.nlm.nih.gov/gene/10133 (accessed Apr 6, 2024).
253. Zhao, S., Chen, R., Gao, Y., Lu, Y., Bai, X., and Zhang, J. (2023) Fundamental roles of the Optineurin gene in the molecular pathology of Amyotrophic Lateral Sclerosis. Frontiers in Neuroscience 17.
254. Ryan, T. A., and Tumbarello, D. A. (2018) Optineurin: A Coordinator of Membrane-Associated Cargo Trafficking and Autophagy. Frontiers in Immunology 9.
255. O'Loughlin, T., Kruppa, A. J., Ribeiro, A. L. R., Edgar, J. R., Ghannam, A., Smith, A. M., and Buss, F. (2020) OPTN recruitment to a Golgi-proximal compartment regulates immune signalling and cytokine secretion. Journal of Cell Science 133.
256. Osawa, T., Mizuno, Y., Fujita, Y., Takatama, M., Nakazato, Y., and Okamoto, K. (2011) Optineurin in neurodegenerative diseases. Neuropathology 31, 569-574.
257. Wise, J. P., Jr., and Cannon, J. (2016) From the Cover: Alterations in Optineurin Expression and Localization in Pre-clinical Parkinson's Disease Models. Toxicological Sciences 153, 372-381.
258. Moharir, S. C., Raghawan, A. K., and Swarup, G. (2020) Optineurin promotes aggregation of mutant huntingtin and mutant ataxin-3, and reduces cytotoxicity of aggregates. bioRxiv, 2020.2008.2013.249201.
259. Xu, Y., Liu, Y., Chen, X., Xu, Q., Liu, L., Liu, H., Guo, R., and Qin, Y. (2022) OPTN attenuates the neurotoxicity of abnormal Tau protein by restoring autophagy. Translational Psychiatry 12, 230.
260. Maruyama, H., Morino, H., Ito, H., Izumi, Y., Kato, H., Watanabe, Y., Kinoshita, Y., Kamada, M., Nodera, H., Suzuki, H., et al. (2010) Mutations of optineurin in amyotrophic lateral sclerosis. Nature 465, 223-226.

261. Bo, R. D., Tiloca, C., Pensato, V., Corrado, L., Ratti, A., Ticozzi, N., Corti, S., Castellotti, B., Mazzini, L., Soraràù, G., et al. (2011) Novel optineurin mutations in patients with familial and sporadic amyotrophic lateral sclerosis. Journal of Neurology, Neurosurgery & Psychiatry 82, 1239-1243.
262. Weishaupt, J. H., Waibel, S., Birve, A., Volk, A. E., Mayer, B., Meyer, T., Ludolph, A. C., and Andersen, P. M. (2013) A novel optineurin truncating mutation and three glaucoma-associated missense variants in patients with familial amyotrophic lateral sclerosis in Germany. Neurobiology of Aging 34, 1516.e1519-1516.e1515.
263. Feng, S.-M., Che, C.-H., Feng, S.-Y., Liu, C.-Y., Li, L.-Y., Li, Y.-X., Huang, H.-P., and Zou, Z.-Y. (2019) Novel mutation in optineurin causing aggressive ALS+/−frontotemporal dementia. Annals of Clinical and Translational Neurology 6, 2377-2383.
264. Stezin, A., Chaithra, S. P., Holla, V. V., Kamble, N., Yadav, R., and Pal, P. K. (2019) A novel OPTN variant causing PSP-CBS-like phenotype in familial amyotrophic lateral sclerosis. Parkinsonism & Related Disorders 69, 147-149.
265. Liu, Z., Li, H., Hong, C., Yue, T., Chen, C., Wang, Z., You, Q., Li, C., Xie, H., and Hu, R. (2018) ALS-Associated E478G Mutation in Human OPTN (Optineurin) Promotes Inflammation and Induces Neuronal Cell Death. Frontiers in Immunology 9.
266. Wong, Y. C., and Holzbaur, E. L. F. (2014) Optineurin is an autophagy receptor for damaged mitochondria in parkin-mediated mitophagy that is disrupted by an ALS-linked mutation. Proceedings of the National Academy of Sciences 111, E4439-E4448.
267. Wen, D., Ji, Y., Li, Y., Duan, W., Wang, Y., Li, Z., Tao, M., and Liu, Y. (2024) OPTN gene therapy increases autophagy and protects mitochondria in SOD1-G93A-expressing transgenic mice and cells. The FEBS journal 291, 795-813.
268. SQSTM1 sequestosome 1 [Homo sapiens (human)]. https://www.ncbi.nlm.nih.gov/gene/8878 (accessed Apr 6, 2024).
269. Kumar, A. V., Mills, J., and Lapierre, L. R. (2022) Selective Autophagy Receptor p62/SQSTM1, a Pivotal Player in Stress and Aging. Frontiers in Cell and Developmental Biology 10.
270. Lin, X., Li, S., Zhao, Y., Ma, X., Zhang, K., He, X., and Wang, Z. (2013) Interaction Domains of p62: A Bridge Between p62 and Selective Autophagy. DNA and Cell Biology 32, 220-227.
271. Rubino, E., Rainero, I., Chiò, A., Rogaeva, E., Galimberti, D., Fenoglio, P., Grinberg, Y., Isaia, G., Calvo, A., Gentile, S., et al. (2012) <i>SQSTM1</i> mutations in frontotemporal lobar degeneration and amyotrophic lateral sclerosis. Neurology 79, 1556-1562.
272. Le Ber, I., Camuzat, A., Guerreiro, R., Bouya-Ahmed, K., Bras, J., Nicolas, G., Gabelle, A., Didic, M., De Septenville, A., Millecamps, S., et al. (2013) SQSTM1 Mutations in French Patients With Frontotemporal Dementia or Frontotemporal Dementia With Amyotrophic Lateral Sclerosis. JAMA Neurology 70, 1403-1410.
273. Sun, L., Rong, Z., Li, W., Zheng, H., Xiao, S., and Li, X. (2018) Identification of a Novel Hemizygous SQSTM1 Nonsense Mutation in Atypical Behavioral Variant Frontotemporal Dementia. Frontiers in Aging Neuroscience 10.
274. Benotmane, H. (2021) Dual pathogenic mutations in SQSTM1 and C9orf72 as a cause of frontotemporal dementia. Alzheimer's & Dementia 17, e052078.
275. Dong, W., Cui, M.-C., Hu, W.-Z., Zeng, Q., Wang, Y.-L., Zhang, W., and Huang, Y. (2022) Genetic and Molecular Evaluation of SQSTM1/p62 on the Neuropathologies of Alzheimer’s Disease. Frontiers in Aging Neuroscience 14.
276. Hirano, M., Nakamura, Y., Saigoh, K., Sakamoto, H., Ueno, S., Isono, C., Miyamoto, K., Akamatsu, M., Mitsui, Y., and Kusunoki, S. (2013) Mutations in the gene encoding p62 in Japanese patients with amyotrophic lateral sclerosis. Neurology 80, 458-463.
277. Fecto, F., Yan, J., Vemula, S. P., Liu, E., Yang, Y., Chen, W., Zheng, J. G., Shi, Y., Siddique, N., Arrat, H., et al. (2011) SQSTM1 Mutations in Familial and Sporadic Amyotrophic Lateral Sclerosis. Archives of Neurology 68, 1440-1446.
278. Chen, Y., Zheng, Z.-Z., Chen, X., Huang, R., Yang, Y., Yuan, L., Pan, L., Hadano, S., and Shang, H.-F. (2014) SQSTM1 mutations in Han Chinese populations with sporadic amyotrophic lateral sclerosis. Neurobiology of Aging 35, 726.e727-726.e729.
279. Kwok, C. T., Morris, A., and de Belleroche, J. S. (2014) Sequestosome-1 (SQSTM1) sequence variants in ALS cases in the UK: prevalence and coexistence of SQSTM1 mutations in ALS kindred with PDB. European Journal of Human Genetics 22, 492-496.

280. Brennan, A., Layfield, R., Long, J., Williams, H. E. L., Oldham, N. J., Scott, D., and Searle, M. S. (2022) An ALS-associated variant of the autophagy receptor SQSTM1/p62 reprograms binding selectivity toward the autophagy-related hATG8 proteins. Journal of Biological Chemistry 298.
281. Ma, S., Attarwala, I. Y., and Xie, X.-Q. (2019) SQSTM1/p62: A Potential Target for Neurodegenerative Disease. ACS Chemical Neuroscience 10, 2094-2114.
282. ANG angiogenin [Homo sapiens (human)]. https://www.ncbi.nlm.nih.gov/gene/283 (accessed Apr 6, 2024).
283. Fett, J. W., Strydom, D. J., Lobb, R. R., Alderman, E. M., Bethune, J. L., Riordan, J. F., and Vallee, B. L. (1985) Isolation and characterization of angiogenin, an angiogenic protein from human carcinoma cells. Biochemistry 24, 5480-5486.
284. Kishimoto, K., Liu, S., Tsuji, T., Olson, K. A., and Hu, G.-f. (2005) Endogenous angiogenin in endothelial cells is a general requirement for cell proliferation and angiogenesis. Oncogene 24, 445-456.
285. Lyons, S. M., Fay, M. M., Akiyama, Y., Anderson, P. J., and Ivanov, P. (2017) RNA biology of angiogenin: Current state and perspectives. RNA biology 14, 171-178.
286. Sheng, J., and Xu, Z. (2016) Three decades of research on angiogenin: a review and perspective. Acta Biochimica et Biophysica Sinica 48, 399-410.
287. Wu, D., Yu, W., Kishikawa, H., Folkerth, R. D., Iafrate, A. J., Shen, Y., Xin, W., Sims, K., and Hu, G.-f. (2007) Angiogenin loss-of-function mutations in amyotrophic lateral sclerosis. Annals of Neurology 62, 609-617.
288. Greenway, M. J., Andersen, P. M., Russ, C., Ennis, S., Cashman, S., Donaghy, C., Patterson, V., Swingler, R., Kieran, D., Prehn, J., et al. (2006) ANG mutations segregate with familial and 'sporadic' amyotrophic lateral sclerosis. Nature Genetics 38, 411-413.
289. Thiyagarajan, N., Ferguson, R., Subramanian, V., and Acharya, K. R. (2012) Structural and molecular insights into the mechanism of action of human angiogenin-ALS variants in neurons. Nature Communications 3, 1121.
290. Bradshaw, W. J., Rehman, S., Pham, T. T. K., Thiyagarajan, N., Lee, R. L., Subramanian, V., and Acharya, K. R. (2017) Structural insights into human angiogenin variants implicated in Parkinson's disease and Amyotrophic Lateral Sclerosis. Scientific reports 7, 41996.
291. Aluri, K. C., Salisbury, J. P., Prehn, J. H. M., and Agar, J. N. (2020) Loss of angiogenin function is related to earlier ALS onset and a paradoxical increase in ALS duration. Scientific reports 10, 3715.
292. VAPB VAMP associated protein B and C [Homo sapiens (human)]. https://www.ncbi.nlm.nih.gov/gene/9217 (accessed Apr 6, 2024).
293. Skehel, P. A., Fabian-Fine, R., and Kandel, E. R. (2000) Mouse VAP33 is associated with the endoplasmic reticulum and microtubules. Proceedings of the National Academy of Sciences 97, 1101-1106.
294. Kors, S., Costello, J. L., and Schrader, M. (2022) VAP Proteins – From Organelle Tethers to Pathogenic Host Interactors and Their Role in Neuronal Disease. Frontiers in Cell and Developmental Biology 10.
295. Loewen, C. J. R., Roy, A., and Levine, T. P. (2003) A conserved ER targeting motif in three families of lipid binding proteins and in Opi1p binds VAP. The EMBO Journal 22, 2025-2035.
296. Rao, M., Song, W., Jiang, A., Shyr, Y., Lev, S., Greenstein, D., Brantley-Sieders, D., and Chen, J. (2012) VAMP-Associated Protein B (VAPB) Promotes Breast Tumor Growth by Modulation of Akt Activity. PloS one 7, e46281.
297. Faria Assoni, A., Giove Mitsugi, T., Wardenaar, R., Oliveira Ferreira, R., Farias Jandrey, E. H., Machado Novaes, G., Fonseca de Oliveira Granha, I., Bakker, P., Kaid, C., Zatz, M., et al. (2023) Neurodegeneration-associated protein VAPB regulates proliferation in medulloblastoma. Scientific reports 13, 19481.
298. Nishimura, A. L., Al-Chalabi, A., and Zatz, M. (2005) A common founder for amyotrophic lateral sclerosis type 8 (ALS8) in the Brazilian population. Human Genetics 118, 499-500.
299. Chadi, G., Maximino, J. R., Jorge, F. M. d. H., Borba, F. C. d., Gilio, J. M., Callegaro, D., Lopes, C. G., Santos, S. N. D., and Rebelo, G. N. S. (2017) Genetic analysis of patients with familial and sporadic amyotrophic lateral sclerosis in a Brazilian Research Center. Amyotrophic Lateral Sclerosis and Frontotemporal Degeneration 18, 249-255.
300. Kanekura, K., Nishimoto, I., Aiso, S., and Matsuoka, M. (2006) Characterization of Amyotrophic Lateral Sclerosis-linked P56S Mutation of Vesicle-associated Membrane Protein-associated Protein B (VAPB/ALS8)*. Journal of Biological Chemistry 281, 30223-30233.

301. Suzuki, H., and Matsuoka, M. (2011) Amyotrophic lateral sclerosis-linked mutant VAPB enhances TDP-43-induced motor neuronal toxicity. Journal of Neurochemistry 119, 1099-1107.
302. Funke, A., Esser, M., Krüttgen, A., Weis, J., Mitne-Neto, M., Lazar, M., Nishimura, A., Sperfeld, A., Trillenberg, P., Senderek, J., et al. (2010) The p.P56S mutation in the VAPB gene is not due to a single founder: the first European case. Clinical Genetics 77, 302-303.
303. Aliaga, L., Lai, C., Yu, J., Chub, N., Shim, H., Sun, L., Xie, C., Yang, W.-J., Lin, X., O'Donovan, M. J., and Cai, H. (2013) Amyotrophic lateral sclerosis-related VAPB P56S mutation differentially affects the function and survival of corticospinal and spinal motor neurons. Human Molecular Genetics 22, 4293-4305.
304. Moustaqim-Barrette, A., Lin, Y. Q., Pradhan, S., Neely, G. G., Bellen, H. J., and Tsuda, H. (2013) The amyotrophic lateral sclerosis 8 protein, VAP, is required for ER protein quality control. Human Molecular Genetics 23, 1975-1989.
305. Di, L., Chen, H., Da, Y., Wang, S., and Shen, X.-M. (2016) Atypical familial amyotrophic lateral sclerosis with initial symptoms of pain or tremor in a Chinese family harboring VAPB-P56S mutation. Journal of Neurology 263, 263-268.
306. Guber, R. D., Schindler, A. B., Budron, M. S., Chen, K.-l., Li, Y., Fischbeck, K. H., and Grunseich, C. (2018) Nucleocytoplasmic transport defect in a North American patient with ALS8. Annals of Clinical and Translational Neurology 5, 369-375.
307. Tripathi, P., Guo, H., Dreser, A., Yamoah, A., Sechi, A., Jesse, C. M., Katona, I., Doukas, P., Nikolin, S., Ernst, S., et al. (2021) Pathomechanisms of ALS8: altered autophagy and defective RNA binding protein (RBP) homeostasis due to the VAPB P56S mutation. Cell Death & Disease 12, 466.
308. Fasana, E., Fossati, M., Ruggiano, A., Brambillasca, S., Hoogenraad, C. C., Navone, F., Francolini, M., and Borgese, N. (2010) A VAPB mutant linked to amyotrophic lateral sclerosis generates a novel form of organized smooth endoplasmic reticulum. The FASEB Journal 24, 1419-1430.
309. Sun, Y.-m., Dong, Y., Wang, J., Lu, J.-h., Chen, Y., and Wu, J.-j. (2017) A novel mutation of VAPB in one Chinese familial amyotrophic lateral sclerosis pedigree and its clinical characteristics. Journal of Neurology 264, 2387-2393.
310. Chen, H.-J., Anagnostou, G., Chai, A., Withers, J., Morris, A., Adhikaree, J., Pennetta, G., and de Belleroche, J. S. (2010) Characterization of the Properties of a Novel Mutation in VAPB in Familial Amyotrophic Lateral Sclerosis*. Journal of Biological Chemistry 285, 40266-40281.
311. van Blitterswijk, M., van Es, M. A., Koppers, M., van Rheenen, W., Medic, J., Schelhaas, H. J., van der Kooi, A. J., de Visser, M., Veldink, J. H., and van den Berg, L. H. (2012) VAPB and C9orf72 mutations in 1 familial amyotrophic lateral sclerosis patient. Neurobiology of Aging 33, 2950.e2951-2950.e2954.
312. Landers, J. E., Leclerc, A. L., Shi, L., Virkud, A., Cho, T., Maxwell, M. M., Henry, A. F., Polak, M., Glass, J. D., Kwiatkowski, T. J., et al. (2008) New <i>VAPB</i> deletion variant and exclusion of <i>VAPB</i> mutations in familial ALS. Neurology 70, 1179-1185.
313. PRPH peripherin [Homo sapiens (human)]. https://www.ncbi.nlm.nih.gov/gene/5630 (accessed Apr 6, 2024).
314. Hol, E. M., and Capetanaki, Y. (2017) Type III Intermediate Filaments Desmin, Glial Fibrillary Acidic Protein (GFAP), Vimentin, and Peripherin. Cold Spring Harbor Perspectives in Biology 9.
315. Parysek, L., and Goldman, R. (1988) Distribution of a novel 57 kDa intermediate filament (IF) protein in the nervous system. The Journal of Neuroscience 8, 555-563.
316. Troy, C. M., Muma, N. A., Greene, L. A., Price, D. L., and Shelanski, M. L. (1990) Regulation of peripherin and neurofilament expression in regenerating rat motor neurons. Brain Research 529, 232-238.
317. Helfand, B. T., Mendez, M. G., Pugh, J., Delsert, C., and Goldman, R. D. (2003) A Role for Intermediate Filaments in Determining and Maintaining the Shape of Nerve Cells. Molecular Biology of the Cell 14, 5069-5081.
318. Sabbatini, D., Raggi, F., Ruggero, S., Seguso, M., Mandrioli, J., Cagnin, A., Briani, C., Toffanin, E., Gizzi, M., Fortuna, A., et al. (2021) Evaluation of peripherin in biofluids of patients with motor neuron diseases. Annals of Clinical and Translational Neurology 8, 1750-1754.
319. Keddie, S., Smyth, D., Keh, R. Y. S., Chou, M. K. L., Grant, D., Surana, S., Heslegrave, A., Zetterberg, H., Wieske, L., Michael, M., et al. (2023) Peripherin is a biomarker of axonal damage in peripheral nervous system disease. Brain 146, 4562-4573.
320. Xiao, S., Tjostheim, S., Sanelli, T., McLean, J. R., Horne, P., Fan, Y., Ravits, J., Strong, M. J., and Robertson, J. (2008) An Aggregate-Inducing Peripherin Isoform Generated through Intron Retention Is Upregulated in Amyotrophic Lateral Sclerosis and Associated with Disease Pathology. The Journal of Neuroscience 28, 1833-1840.

321. Robertson , J., Doroudchi , M. M., Nguyen , M. D., Durham , H. D., Strong , M. J., Shaw , G., Julien , J.-P., and Mushynski , W. E. (2003) A neurotoxic peripherin splice variant in a mouse model of ALS. Journal of Cell Biology 160, 939-949.
322. Corrado, L., Carlomagno, Y., Falasco, L., Mellone, S., Godi, M., Cova, E., Cereda, C., Testa, L., Mazzini, L., and D'Alfonso, S. (2011) A novel peripherin gene (PRPH) mutation identified in one sporadic amyotrophic lateral sclerosis patient. Neurobiology of Aging 32, 552.e551-552.e556.
323. CHCHD10 coiled-coil-helix-coiled-coil-helix domain containing 10 [Homo sapiens (human)]. https://www.ncbi.nlm.nih.gov/gene/400916 (accessed Apr 6, 2024).
324. Modjtahedi, N., Tokatlidis, K., Dessen, P., and Kroemer, G. (2016) Mitochondrial Proteins Containing Coiled-Coil-Helix-Coiled-Coil-Helix (CHCH) Domains in Health and Disease. Trends in Biochemical Sciences 41, 245-260.
325. Q8WYQ3 · CHC10_HUMAN. https://www.uniprot.org/uniprotkb/Q8WYQ3/entry (accessed Apr 9, 2024).
326. Penttilä, S., Jokela, M., Bouquin, H., Saukkonen, A. M., Toivanen, J., and Udd, B. (2015) Late onset spinal motor neuronopathy is caused by mutation in CHCHD10. Annals of Neurology 77, 163-172.
327. Ajroud-Driss, S., Fecto, F., Ajroud, K., Lalani, I., Calvo, S. E., Mootha, V. K., Deng, H.-X., Siddique, N., Tahmoush, A. J., Heiman-Patterson, T. D., and Siddique, T. (2015) Mutation in the novel nuclear-encoded mitochondrial protein CHCHD10 in a family with autosomal dominant mitochondrial myopathy. neurogenetics 16, 1-9.
328. Chaussenot, A., Le Ber, I., Ait-El-Mkadem, S., Camuzat, A., de Septenville, A., Bannwarth, S., Genin, E. C., Serre, V., Augé, G., Brice, A., et al. (2014) Screening of CHCHD10 in a French cohort confirms the involvement of this gene in frontotemporal dementia with amyotrophic lateral sclerosis patients. Neurobiology of Aging 35, 2884.e2881-2884.e2884.
329. Bannwarth, S., Ait-El-Mkadem, S., Chaussenot, A., Genin, E. C., Lacas-Gervais, S., Fragaki, K., Berg-Alonso, L., Kageyama, Y., Serre, V., Moore, D. G., et al. (2014) A mitochondrial origin for frontotemporal dementia and amyotrophic lateral sclerosis through CHCHD10 involvement. Brain 137, 2329-2345.
330. Chiò, A., Mora, G., Sabatelli, M., Caponnetto, C., Traynor, B. J., Johnson, J. O., Nalls, M. A., Calvo, A., Moglia, C., Borghero, G., et al. (2015) CHCH10 mutations in an Italian cohort of familial and sporadic amyotrophic lateral sclerosis patients. Neurobiology of Aging 36, 1767.e1763-1767.e1766.
331. Jiao, B., Xiao, T., Hou, L., Gu, X., Zhou, Y., Zhou, L., Tang, B., Xu, J., and Shen, L. (2015) High prevalence of CHCHD10 mutation in patients with frontotemporal dementia from China. Brain 139, e21-e21.
332. TBK1 TANK binding kinase 1 [Homo sapiens (human)]. https://www.ncbi.nlm.nih.gov/gene/29110 (accessed Apr 6, 2024).
333. Runde, A. P., Mack, R., S.J, P. B., and Zhang, J. (2022) The role of TBK1 in cancer pathogenesis and anticancer immunity. Journal of Experimental & Clinical Cancer Research 41, 135.
334. Larabi, A., Devos, Juliette M., Ng, S.-L., Nanao, Max H., Round, A., Maniatis, T., and Panne, D. (2013) Crystal Structure and Mechanism of Activation of TANK-Binding Kinase 1. Cell Reports 3, 734-746.
335. Zhang, C., Shang, G., Gui, X., Zhang, X., Bai, X.-c., and Chen, Z. J. (2019) Structural basis of STING binding with and phosphorylation by TBK1. Nature 567, 394-398.
336. Ma, X., Helgason, E., Phung, Q. T., Quan, C. L., Iyer, R. S., Lee, M. W., Bowman, K. K., Starovasnik, M. A., and Dueber, E. C. (2012) Molecular basis of Tank-binding kinase 1 activation by transautophosphorylation. Proceedings of the National Academy of Sciences 109, 9378-9383.
337. Revach, O.-Y., Liu, S., and Jenkins, R. W. (2020) Targeting TANK-binding kinase 1 (TBK1) in cancer. Expert Opinion on Therapeutic Targets 24, 1065-1078.
338. Sun, Y., Revach, O.-y., Anderson, S., Kessler, E. A., Wolfe, C. H., Jenney, A., Mills, C. E., Robitschek, E. J., Davis, T. G. R., Kim, S., et al. (2023) Targeting TBK1 to overcome resistance to cancer immunotherapy. Nature 615, 158-167.
339. Hasan, M., and Yan, N. (2016) Therapeutic potential of targeting TBK1 in autoimmune diseases and interferonopathies. Pharmacological Research 111, 336-342.
340. Bodewes, I. L. A., Huijser, E., van Helden-Meeuwsen, C. G., Tas, L., Huizinga, R., Dalm, V. A. S. H., van Hagen, P. M., Groot, N., Kamphuis, S., van Daele, P. L. A., and Versnel, M. A. (2018) TBK1: A key regulator and potential treatment target for interferon positive Sjögren's syndrome, systemic lupus erythematosus and systemic sclerosis. Journal of Autoimmunity 91, 97-102.

341. Ding, C., Song, Z., Shen, A., Chen, T., and Zhang, A. (2020) Small molecules targeting the innate immune cGAS–STING–TBK1 signaling pathway. Acta Pharmaceutica Sinica B 10, 2272-2298.
342. Thomson, D. W., and Bergamini, G. (2021) Recent progress in small molecule TBK1 inhibitors: a patent review (2015– 2020). Expert Opinion on Therapeutic Patents 31, 785-794.
343. Cirulli, E. T., Lasseigne, B. N., Petrovski, S., Sapp, P. C., Dion, P. A., Leblond, C. S., Couthouis, J., Lu, Y.-F., Wang, Q., Krueger, B. J., et al. (2015) Exome sequencing in amyotrophic lateral sclerosis identifies risk genes and pathways. Science (New York, N.Y.) 347, 1436-1441.
344. Tsai, P.-C., Liu, Y.-C., Lin, K.-P., Liu, Y.-T., Liao, Y.-C., Hsiao, C.-T., Soong, B.-W., Yip, P.-K., and Lee, Y.-C. (2016) Mutational analysis of TBK1 in Taiwanese patients with amyotrophic lateral sclerosis. Neurobiology of Aging 40, 191.e111-191.e116.
345. Piaceri, I., Bessi, V., Matà, S., Polito, C., Tedde, A., Berti, V., Bagnoli, S., Braccia, A., Del Mastio, M., Pignone, A. M., et al. (2018) Association of the New Variant Tyr424Asp at TBK1 Gene with Amyotrophic Lateral Sclerosis and Cognitive Decline. Journal of Alzheimer's Disease 61, 41-46.
346. Oakes, J. A., Davies, M. C., and Collins, M. O. (2017) TBK1: a new player in ALS linking autophagy and neuroinflammation. Molecular Brain 10, 5.
347. Ye, J., Cheung, J., Gerbino, V., Ahlsén, G., Zimanyi, C., Hirsh, D., and Maniatis, T. (2019) Effects of ALS-associated TANK binding kinase 1 mutations on protein–protein interactions and kinase activity. Proceedings of the National Academy of Sciences 116, 24517-24526.
348. Le Ber, I., De Septenville, A., Millecamps, S., Camuzat, A., Caroppo, P., Couratier, P., Blanc, F., Lacomblez, L., Sellal, F., Fleury, M.-C., et al. (2015) TBK1 mutation frequencies in French frontotemporal dementia and amyotrophic lateral sclerosis cohorts. Neurobiology of Aging 36, 3116.e3115-3116.e3118.
349. Gijselinck, I., Van Mossevelde, S., van der Zee, J., Sieben, A., Philtjens, S., Heeman, B., Engelborghs, S., Vandenbulcke, M., De Baets, G., Bäumer, V., et al. (2015) Loss of <i>TBK1</i> is a frequent cause of frontotemporal dementia in a Belgian cohort. Neurology 85, 2116-2125.
350. NEFH neurofilament heavy chain [Homo sapiens (human)]. https://www.ncbi.nlm.nih.gov/gene/4744 (accessed Apr 6, 2024).
351. Yuan, A., Rao, M. V., Veeranna, and Nixon, R. A. (2017) Neurofilaments and Neurofilament Proteins in Health and Disease. Cold Spring Harbor Perspectives in Biology 9.
352. Lee, M., Xu, Z., Wong, P., and Cleveland, D. (1993) Neurofilaments are obligate heteropolymers in vivo. Journal of Cell Biology 122, 1337-1350.
353. Hoffman, P. N., Cleveland, D. W., Griffin, J. W., Landes, P. W., Cowan, N. J., and Price, D. L. (1987) Neurofilament gene expression: a major determinant of axonal caliber. Proceedings of the National Academy of Sciences 84, 3472-3476.
354. Jacquier, A., Delorme, C., Belotti, E., Juntas-Morales, R., Solé, G., Dubourg, O., Giroux, M., Maurage, C.-A., Castellani, V., Rebelo, A., et al. (2017) Cryptic amyloidogenic elements in mutant NEFH causing Charcot-Marie-Tooth 2 trigger aggresome formation and neuronal death. Acta Neuropathologica Communications 5, 55.
355. Ikenberg, E., Reilich, P., Abicht, A., Heller, C., Schoser, B., and Walter, M. C. (2019) Charcot-Marie-Tooth disease type 2CC due to a frameshift mutation of the neurofilament heavy polypeptide gene in an Austrian family. Neuromuscular Disorders 29, 392-397.
356. Pipis, M., Cortese, A., Polke, J. M., Poh, R., Vandrovcova, J., Laura, M., Skorupinska, M., Jacquier, A., Juntas-Morales, R., Latour, P., et al. (2022) Charcot-Marie-Tooth disease type 2CC due to NEFH variants causes a progressive, non-length-dependent, motor-predominant phenotype. Journal of Neurology, Neurosurgery & Psychiatry 93, 48-56.
357. Figlewicz, D. A., Krizus, A., Martinoli, M. G., Meininger, V., Dib, M., Rouleau, G. A., and Julien, J.-P. (1994) Variants of the heavy neurofilament subunit are associated with the development of amyotrophic lateral sclerosis. Human Molecular Genetics 3, 1757-1761.
358. Tomkins, J., Usher, P., Slade, J. Y., Ince, P. G., Curtis, A., Bushby, K., and Shaw, P. J. (1998) Novel insertion in the KSP region of the neurofilament heavy gene in amyotrophic lateral sclerosis (ALS). NeuroReport 9, 3967-3970.
359. Al-Chalabi, A., Andersen, P. M., Nilsson, P., Chioza, B., Andersson, J. L., Russ, C., Shaw, C. E., Powell, J. F., and Leigh, P. N. (1999) Deletions of the heavy neurofilament subunit tail in amyotrophic lateral sclerosis. Hum Mol Genet 8, 157-164.
360. Lin, F., Lin, W., Zhu, C., Lin, J., Zhu, J., Li, X.-Y., Wang, Z., Wang, C., and Huang, H. (2021) Sequencing of neurofilament genes identified NEFH Ser787Arg as a novel risk variant of sporadic amyotrophic lateral sclerosis in Chinese subjects. BMC Medical Genomics 14, 222.

361. FUS FUS RNA binding protein [Homo sapiens (human)]. https://www.ncbi.nlm.nih.gov/gene/2521 (accessed Apr 6, 2024).
362. Dormann, D., and Haass, C. (2013) Fused in sarcoma (FUS): An oncogene goes awry in neurodegeneration. Molecular and Cellular Neuroscience 56, 475-486.
363. Jia, W., Kim, S. H., Scalf, M. A., Tonzi, P., Millikin, R. J., Guns, W. M., Liu, L., Mastrocola, A. S., Smith, L. M., Huang, T. T., and Tibbetts, R. S. (2021) Fused in sarcoma regulates DNA replication timing and kinetics. Journal of Biological Chemistry 297.
364. Kodavati, M., Wang, H., Guo, W., Mitra, J., Hegde, P. M., Provasek, V., Rao, V. H. M., Vedula, I., Zhang, A., Mitra, S., et al. (2024) FUS unveiled in mitochondrial DNA repair and targeted ligase-1 expression rescues repair-defects in FUS-linked motor neuron disease. Nature Communications 15, 2156.
365. Deng, H., Gao, K., and Jankovic, J. (2014) The role of FUS gene variants in neurodegenerative diseases. Nature Reviews Neurology 10, 337-348.
366. Neumann, M., Rademakers, R., Roeber, S., Baker, M., Kretzschmar, H. A., and Mackenzie, I. R. A. (2009) A new subtype of frontotemporal lobar degeneration with FUS pathology. Brain 132, 2922-2931.
367. Kwiatkowski, T. J., Bosco, D. A., LeClerc, A. L., Tamrazian, E., Vanderburg, C. R., Russ, C., Davis, A., Gilchrist, J., Kasarskis, E. J., Munsat, T., et al. (2009) Mutations in the <i>FUS/TLS</i> Gene on Chromosome 16 Cause Familial Amyotrophic Lateral Sclerosis. Science (New York, N.Y.) 323, 1205-1208.
368. Vance, C., Rogelj, B., Hortobágyi, T., De Vos, K. J., Nishimura, A. L., Sreedharan, J., Hu, X., Smith, B., Ruddy, D., Wright, P., et al. (2009) Mutations in FUS, an RNA Processing Protein, Cause Familial Amyotrophic Lateral Sclerosis Type 6. Science (New York, N.Y.) 323, 1208-1211.
369. Chiò, A., Restagno, G., Brunetti, M., Ossola, I., Calvo, A., Mora, G., Sabatelli, M., Monsurrò, M. R., Battistini, S., Mandrioli, J., et al. (2009) Two Italian kindreds with familial amyotrophic lateral sclerosis due to FUS mutation. Neurobiology of Aging 30, 1272-1275.
370. Ticozzi, N., Silani, V., LeClerc, A. L., Keagle, P., Gellera, C., Ratti, A., Taroni, F., Kwiatkowski, T. J., Jr., McKenna-Yasek, D. M., Sapp, P. C., et al. (2009) Analysis of FUS gene mutation in familial amyotrophic lateral sclerosis within an Italian cohort. Neurology 73, 1180-1185.
371. Millecamps, S., Salachas, F., Cazeneuve, C., Gordon, P., Bricka, B., Camuzat, A., Guillot-Noël, L., Russaouen, O., Bruneteau, G., Pradat, P.-F., et al. (2010) SOD1, ANG, VAPB, TARDBP, and FUS mutations in familial amyotrophic lateral sclerosis: genotype–phenotype correlations. Journal of Medical Genetics 47, 554-560.
372. Hou, L., Jiao, B., Xiao, T., Zhou, L., Zhou, Z., Du, J., Yan, X., Wang, J., Tang, B., and Shen, L. (2016) Screening of SOD1, FUS and TARDBP genes in patients with amyotrophic lateral sclerosis in central-southern China. Scientific reports 6, 32478.
373. Liu, Z.-J., Lin, H.-X., Liu, G.-L., Tao, Q.-Q., Ni, W., Xiao, B.-G., and Wu, Z.-Y. (2017) The investigation of genetic and clinical features in Chinese patients with juvenile amyotrophic lateral sclerosis. Clinical Genetics 92, 267-273.
374. Dodd, K. C., Power, R., Ealing, J., and Hamdalla, H. (2019) FUS-ALS presenting with myoclonic jerks in a 17-year-old man. Amyotrophic Lateral Sclerosis and Frontotemporal Degeneration 20, 278-280.
375. Mboukou, A., Rajendra, V., Kleinova, R., Tisné, C., Jantsch, M. F., and Barraud, P. (2021) Transportin-1: A Nuclear Import Receptor with Moonlighting Functions. Frontiers in Molecular Biosciences 8.
376. Dormann, D., Rodde, R., Edbauer, D., Bentmann, E., Fischer, I., Hruscha, A., Than, M. E., Mackenzie, I. R. A., Capell, A., Schmid, B., et al. (2010) ALS‐associated fused in sarcoma (<i>FUS</i>) mutations disrupt Transportin‐mediated nuclear import. The EMBO Journal 29, 2841-2857.
377. Nakaya, T., and Maragkakis, M. (2018) Amyotrophic Lateral Sclerosis associated FUS mutation shortens mitochondria and induces neurotoxicity. Scientific reports 8, 15575.
378. PFN1 profilin 1 [Homo sapiens (human)]. https://www.ncbi.nlm.nih.gov/gene/5216 (accessed Apr 6, 2024).
379. Alkam, D., Feldman, E. Z., Singh, A., and Kiaei, M. (2017) Profilin1 biology and its mutation, actin(g) in disease. Cellular and Molecular Life Sciences 74, 967-981.
380. Karlsson, R., and Lindberg, U., Profilin, an Essential Control Element for Actin Polymerization. In *Actin-Monomer-Binding Proteins*, Lappalainen, P., Ed. Springer New York: New York, NY, 2007; pp 29-44.
381. Witke, W., Sutherland, J. D., Sharpe, A., Arai, M., and Kwiatkowski, D. J. (2001) Profilin I is essential for cell survival and cell division in early mouse development. Proceedings of the National Academy of Sciences 98, 3832-3836.

382. Witke, W. (2004) The role of profilin complexes in cell motility and other cellular processes. Trends in Cell Biology 14, 461-469.
383. Jockusch, B. M., Murk, K., and Rothkegel, M., The profile of profilins. In *Reviews of Physiology, Biochemistry and Pharmacology*, Amara, S. G.; Bamberg, E.; Fleischmann, B.; Gudermann, T.; Hebert, S. C.; Jahn, R.; Lederer, W. J.; Lill, R.; Miyajima, A.; Offermanns, S.; Zechner, R., Eds. Springer Berlin Heidelberg: Berlin, Heidelberg, 2007; pp 131-149.
384. Wang, Y., Wang, Y., Wan, R., Hu, C., and Lu, Y. (2021) Profilin 1 Protein and Its Implications for Cancers. Oncology (Williston Park) 35, 402-409.
385. Zou, L., Jaramillo, M., Whaley, D., Wells, A., Panchapakesa, V., Das, T., and Roy, P. (2007) Profilin-1 is a negative regulator of mammary carcinoma aggressiveness. British Journal of Cancer 97, 1361-1371.
386. George, L., Winship, A., Sorby, K., Dimitriadis, E., and Menkhorst, E. (2020) Profilin-1 is dysregulated in endometroid (type I) endometrial cancer promoting cell proliferation and inhibiting pro-inflammatory cytokine production. Biochemical and Biophysical Research Communications 531, 459-464.
387. Wang, Y., Lu, Y., Wan, R., Wang, Y., Zhang, C., Li, M., Deng, P., Cao, L., and Hu, C. (2022) Profilin 1 Induces Tumor Metastasis by Promoting Microvesicle Secretion Through the ROCK 1/p-MLC Pathway in Non-Small Cell Lung Cancer. Frontiers in Pharmacology 13.
388. Wu, C.-H., Fallini, C., Ticozzi, N., Keagle, P. J., Sapp, P. C., Piotrowska, K., Lowe, P., Koppers, M., McKenna-Yasek, D., Baron, D. M., et al. (2012) Mutations in the profilin 1 gene cause familial amyotrophic lateral sclerosis. Nature 488, 499-503.
389. Tanaka, Y., Nonaka, T., Suzuki, G., Kametani, F., and Hasegawa, M. (2016) Gain-of-function profilin 1 mutations linked to familial amyotrophic lateral sclerosis cause seed-dependent intracellular TDP-43 aggregation. Human Molecular Genetics 25, 1420-1433.
390. Chi, J., Chen, J., Li, Y., Huang, Z., Wang, L., and Zhang, Y. (2020) A Familial Phenotypic and Genetic Study of Mutations in PFN1 Associated with Amyotrophic Lateral Sclerosis. Neuroscience Bulletin 36, 174-178.
391. Ingre, C., Landers, J. E., Rizik, N., Volk, A. E., Akimoto, C., Birve, A., Hübers, A., Keagle, P. J., Piotrowska, K., Press, R., et al. (2013) A novel phosphorylation site mutation in profilin 1 revealed in a large screen of US, Nordic, and German amyotrophic lateral sclerosis/frontotemporal dementia cohorts. Neurobiology of Aging 34, 1708.e1701-1708.e1706.
392. Pereira, G. R. C., Tellini, G. H. A. S., and De Mesquita, J. F. (2019) In silico analysis of PFN1 related to amyotrophic lateral sclerosis. PloS one 14, e0215723.
393. MATR3 matrin 3 [Homo sapiens (human)]. https://www.ncbi.nlm.nih.gov/gene/9782 (accessed Apr 6, 2024).
394. P43243 · MATR3_HUMAN. https://www.uniprot.org/uniprotkb/P43243/entry (accessed Apr 6, 2024).
395. Malik, A. M., and Barmada, S. J. (2021) Matrin 3 in neuromuscular disease: physiology and pathophysiology. JCI Insight 6.
396. Salem, A., Wilson, C. J., Rutledge, B. S., Dilliott, A., Farhan, S., Choy, W.-Y., and Duennwald, M. L. (2022) Matrin3: Disorder and ALS Pathogenesis. Frontiers in Molecular Biosciences 8.
397. Salton, M., Elkon, R., Borodina, T., Davydov, A., Yaspo, M.-L., Halperin, E., and Shiloh, Y. (2011) Matrin 3 Binds and Stabilizes mRNA. PloS one 6, e23882.
398. Coelho, M. B., Attig, J., Bellora, N., König, J., Hallegger, M., Kayikci, M., Eyras, E., Ule, J., and Smith, C. W. (2015) Nuclear matrix protein Matrin3 regulates alternative splicing and forms overlapping regulatory networks with PTB. The EMBO Journal 34, 653-668.
399. Salton, M., Lerenthal, Y., Wang, S.-Y., Chen, D. J., and Shiloh, Y. (2010) Involvement of Matrin 3 and SFPQ/NONO in the DNA damage response. Cell Cycle 9, 1568-1576.
400. Johnson, J. O., Pioro, E. P., Boehringer, A., Chia, R., Feit, H., Renton, A. E., Pliner, H. A., Abramzon, Y., Marangi, G., Winborn, B. J., et al. (2014) Mutations in the Matrin 3 gene cause familial amyotrophic lateral sclerosis. Nature Neuroscience 17, 664-666.
401. Lin, K.-P., Tsai, P.-C., Liao, Y.-C., Chen, W.-T., Tsai, C.-P., Soong, B.-W., and Lee, Y.-C. (2015) Mutational analysis of MATR3 in Taiwanese patients with amyotrophic lateral sclerosis. Neurobiology of Aging 36, 2005.e2001-2005.e2004.
402. Origone, P., Verdiani, S., Bandettini Di Poggio, M., Zuccarino, R., Vignolo, M., Caponnetto, C., and Mandich, P. (2015) A novel Arg147Trp MATR3 missense mutation in a slowly progressive ALS Italian patient. Amyotrophic Lateral Sclerosis and Frontotemporal Degeneration 16, 530-531.

403. Tripolszki, K., Gampawar, P., Schmidt, H., Nagy, Z. F., Nagy, D., Klivényi, P., Engelhardt, J. I., and Széll, M. (2019) Comprehensive Genetic Analysis of a Hungarian Amyotrophic Lateral Sclerosis Cohort. Frontiers in Genetics 10.
404. Narain, P., Padhi, A. K., Dave, U., Mishra, D., Bhatia, R., Vivekanandan, P., and Gomes, J. (2019) Identification and characterization of novel and rare susceptible variants in Indian amyotrophic lateral sclerosis patients. neurogenetics 20, 197-208.
405. UBQLN2 ubiquilin 2 [Homo sapiens (human)]. https://www.ncbi.nlm.nih.gov/gene/29978 (accessed Apr 6, 2024).
406. Zheng, T., Yang, Y., and Castañeda, C. A. (2020) Structure, dynamics and functions of UBQLNs: at the crossroads of protein quality control machinery. Biochemical Journal 477, 3471-3497.
407. Zhang, K. Y., Yang, S., Warraich, S. T., and Blair, I. P. (2014) Ubiquilin 2: A component of the ubiquitin–proteasome system with an emerging role in neurodegeneration. The International Journal of Biochemistry & Cell Biology 50, 123-126.
408. Deng, H.-X., Chen, W., Hong, S.-T., Boycott, K. M., Gorrie, G. H., Siddique, N., Yang, Y., Fecto, F., Shi, Y., Zhai, H., et al. (2011) Mutations in UBQLN2 cause dominant X-linked juvenile and adult-onset ALS and ALS/dementia. Nature 477, 211-215.
409. Synofzik, M., Maetzler, W., Grehl, T., Prudlo, J., vom Hagen, J. M., Haack, T., Rebassoo, P., Munz, M., Schöls, L., and Biskup, S. (2012) Screening in ALS and FTD patients reveals 3 novel UBQLN2 mutations outside the PXX domain and a pure FTD phenotype. Neurobiology of Aging 33, 2949.e2913-2949.e2917.
410. Williams, K. L., Warraich, S. T., Yang, S., Solski, J. A., Fernando, R., Rouleau, G. A., Nicholson, G. A., and Blair, I. P. (2012) UBQLN2/ubiquilin 2 mutation and pathology in familial amyotrophic lateral sclerosis. Neurobiology of Aging 33, 2527.e2523-2527.e2510.
411. Xia, Y., Yan, L. H., Huang, B., Liu, M., Liu, X., and Huang, C. (2014) Pathogenic mutation of UBQLN2 impairs its interaction with UBXD8 and disrupts endoplasmic reticulum-associated protein degradation. Journal of Neurochemistry 129, 99-106.
412. Renaud, L., Picher-Martel, V., Codron, P., and Julien, J.-P. (2019) Key role of UBQLN2 in pathogenesis of amyotrophic lateral sclerosis and frontotemporal dementia. Acta Neuropathologica Communications 7, 103.
413. ALS2 alsin Rho guanine nucleotide exchange factor ALS2 [Homo sapiens (human)]. https://www.ncbi.nlm.nih.gov/gene/57679 (accessed Apr 6, 2024).
414. Topp, J. D., Gray, N. W., Gerard, R. D., and Horazdovsky, B. F. (2004) Alsin Is a Rab5 and Rac1 Guanine Nucleotide Exchange Factor *. Journal of Biological Chemistry 279, 24612-24623.
415. Hadano, S., Otomo, A., Suzuki-Utsunomiya, K., Kunita, R., Yanagisawa, Y., Showguchi-Miyata, J., Mizumura, H., and Ikeda, J.-E. (2004) ALS2CL, the novel protein highly homologous to the carboxy-terminal half of ALS2, binds to Rab5 and modulates endosome dynamics. FEBS Letters 575, 64-70.
416. Devon, R. S., Schwab, C., Topp, J. D., Orban, P. C., Yang, Y.-z., Pape, T. D., Helm, J. R., Davidson, T.-L., Rogers, D. A., Gros-Louis, F., et al. (2005) Cross-species characterization of the ALS2 gene and analysis of its pattern of expression in development and adulthood. Neurobiology of Disease 18, 243-257.
417. Jacquier, A., Buhler, E., Schäfer, M. K. E., Bohl, D., Blanchard, S., Beclin, C., and Haase, G. (2006) Alsin/Rac1 signaling controls survival and growth of spinal motoneurons. Annals of Neurology 60, 105-117.
418. Yang, Y., Hentati, A., Deng, H.-X., Dabbagh, O., Sasaki, T., Hirano, M., Hung, W.-Y., Ouahchi, K., Yan, J., Azim, A. C., et al. (2001) The gene encoding alsin, a protein with three guanine-nucleotide exchange factor domains, is mutated in a form of recessive amyotrophic lateral sclerosis. Nature Genetics 29, 160-165.
419. Kress, J. A., Kühnlein, P., Winter, P., Ludolph, A. C., Kassubek, J., Müller, U., and Sperfeld, A.-D. (2005) Novel mutation in the ALS2 gene in juvenile amyotrophic lateral sclerosis. Annals of Neurology 58, 800-803.
420. Luigetti, M., Lattante, S., Conte, A., Romano, A., Zollino, M., Marangi, G., and Sabatelli, M. (2013) A novel compound heterozygous ALS2 mutation in two Italian siblings with juvenile amyotrophic lateral sclerosis. Amyotrophic Lateral Sclerosis and Frontotemporal Degeneration 14, 470-472.
421. Farrugia Wismayer, M., Farrugia Wismayer, A., Borg, R., Bonavia, K., Abela, A., Chircop, C., Aquilina, J., Soler, D., Pace, A., Vella, M., et al. (2023) Genetic landscape of ALS in Malta based on a quinquennial analysis. Neurobiology of Aging 123, 200-207.
422. FIG4 FIG4 phosphoinositide 5-phosphatase [Homo sapiens (human)]. https://www.ncbi.nlm.nih.gov/gene/9896 (accessed Apr 6, 2024).

423. Chow, C. Y., Zhang, Y., Dowling, J. J., Jin, N., Adamska, M., Shiga, K., Szigeti, K., Shy, M. E., Li, J., Zhang, X., et al. (2007) Mutation of FIG4 causes neurodegeneration in the pale tremor mouse and patients with CMT4J. Nature 448, 68-72.
424. Sbrissa, D., Ikonomov, O. C., Fu, Z., Ijuin, T., Gruenberg, J., Takenawa, T., and Shisheva, A. (2007) Core Protein Machinery for Mammalian Phosphatidylinositol 3,5-Bisphosphate Synthesis and Turnover That Regulates the Progression of Endosomal Transport: NOVEL SAC PHOSPHATASE JOINS THE ArPIKfyve-PIKfyve COMPLEX *. Journal of Biological Chemistry 282, 23878-23891.
425. Takasuga, S., and Sasaki, T. (2013) Phosphatidylinositol-3,5-bisphosphate: metabolism and physiological functions. The Journal of Biochemistry 154, 211-218.
426. Jin, N., Lang, Michael J., and Weisman, Lois S. (2016) Phosphatidylinositol 3,5-bisphosphate: regulation of cellular events in space and time. Biochemical Society Transactions 44, 177-184.
427. Lenk, G. M., Ferguson, C. J., Chow, C. Y., Jin, N., Jones, J. M., Grant, A. E., Zolov, S. N., Winters, J. J., Giger, R. J., Dowling, J. J., et al. (2011) Pathogenic Mechanism of the FIG4 Mutation Responsible for Charcot-Marie-Tooth Disease CMT4J. PLOS Genetics 7, e1002104.
428. Charcot-Marie-Tooth Disease. https://www.ninds.nih.gov/health-information/disorders/charcot-marie-tooth-disease (accessed Apr 9, 2024).
429. Martyn, C., and Li, J. (2013) Fig4 deficiency: A newly emerged lysosomal storage disorder? Progress in Neurobiology 101-102, 35-45.
430. Chow, C. Y., Landers, J. E., Bergren, S. K., Sapp, P. C., Grant, A. E., Jones, J. M., Everett, L., Lenk, G. M., McKenna-Yasek, D. M., Weisman, L. S., et al. (2009) Deleterious Variants of FIG4, a Phosphoinositide Phosphatase, in Patients with ALS. The American Journal of Human Genetics 84, 85-88.
431. Cady, J., Allred, P., Bali, T., Pestronk, A., Goate, A., Miller, T. M., Mitra, R. D., Ravits, J., Harms, M. B., and Baloh, R. H. (2015) Amyotrophic lateral sclerosis onset is influenced by the burden of rare variants in known amyotrophic lateral sclerosis genes. Annals of Neurology 77, 100-113.
432. Nakamura, R., Sone, J., Atsuta, N., Tohnai, G., Watanabe, H., Yokoi, D., Nakatochi, M., Watanabe, H., Ito, M., Senda, J., et al. (2016) Next-generation sequencing of 28 ALS-related genes in a Japanese ALS cohort. Neurobiology of Aging 39, 219.e211-219.e218.
433. Krüger, S., Battke, F., Sprecher, A., Munz, M., Synofzik, M., Schöls, L., Gasser, T., Grehl, T., Prudlo, J., and Biskup, S. (2016) Rare Variants in Neurodegeneration Associated Genes Revealed by Targeted Panel Sequencing in a German ALS Cohort. Frontiers in Molecular Neuroscience 9.
434. Osmanovic, A., Rangnau, I., Kosfeld, A., Abdulla, S., Janssen, C., Auber, B., Raab, P., Preller, M., Petri, S., and Weber, R. G. (2017) FIG4 variants in central European patients with amyotrophic lateral sclerosis: a whole-exome and targeted sequencing study. European Journal of Human Genetics 25, 324-331.
435. Zhang, H., Cai, W., Chen, S., Liang, J., Wang, Z., Ren, Y., Liu, W., Zhang, X., Sun, Z., and Huang, X. (2018) Screening for possible oligogenic pathogenesis in Chinese sporadic ALS patients. Amyotrophic Lateral Sclerosis and Frontotemporal Degeneration 19, 419-425.
436. Liu, C.-Y., Lin, J.-L., Feng, S.-Y., Che, C.-H., Huang, H.-P., and Zou, Z.-Y. (2022) Novel Variants in the FIG4 Gene Associated With Chinese Sporadic Amyotrophic Lateral Sclerosis With Slow Progression. J Clin Neurol 18, 41-47.
437. Zhang, X., Chow, C. Y., Sahenk, Z., Shy, M. E., Meisler, M. H., and Li, J. (2008) Mutation of FIG4 causes a rapidly progressive, asymmetric neuronal degeneration. Brain 131, 1990-2001.
438. KIF5A kinesin family member 5A [Homo sapiens (human)]. https://www.ncbi.nlm.nih.gov/gene/3798 (accessed Apr 6, 2024).
439. Miki, H., Setou, M., Kaneshiro, K., and Hirokawa, N. (2001) All kinesin superfamily protein, KIF, genes in mouse and human. Proceedings of the National Academy of Sciences 98, 7004-7011.
440. Wang, N., and Xu, J. (2015) Functions of Kinesin Superfamily Proteins in Neuroreceptor Trafficking. BioMed Research International 2015, 639301.
441. Hirokawa, N., Noda, Y., Tanaka, Y., and Niwa, S. (2009) Kinesin superfamily motor proteins and intracellular transport. Nature Reviews Molecular Cell Biology 10, 682-696.
442. Nakagawa, T., Tanaka, Y., Matsuoka, E., Kondo, S., Okada, Y., Noda, Y., Kanai, Y., and Hirokawa, N. (1997) Identification and classification of 16 new kinesin superfamily (KIF) proteins in mouse genome. Proceedings of the National Academy of Sciences 94, 9654-9659.
443. Nakajima, K., Yin, X., Takei, Y., Seog, D.-H., Homma, N., and Hirokawa, N. (2012) Molecular Motor KIF5A Is Essential for GABA_A Receptor Transport, and KIF5A Deletion Causes Epilepsy. Neuron 76, 945-961.

444. Kaan, H. Y. K., Hackney, D. D., and Kozielski, F. (2011) The Structure of the Kinesin-1 Motor-Tail Complex Reveals the Mechanism of Autoinhibition. Science (New York, N.Y.) 333, 883-885.
445. Blasius , T. L., Cai , D., Jih , G. T., Toret , C. P., and Verhey , K. J. (2007) Two binding partners cooperate to activate the molecular motor Kinesin-1. Journal of Cell Biology 176, 11-17.
446. Hares, K., Miners, J. S., Cook, A. J., Rice, C., Scolding, N., Love, S., and Wilkins, A. (2017) Overexpression of Kinesin Superfamily Motor Proteins in Alzheimer's Disease. Journal of Alzheimer's Disease 60, 1511-1524.
447. Wang, Q., Tian, J., Chen, H., Du, H., and Guo, L. (2019) Amyloid beta-mediated KIF5A deficiency disrupts anterograde axonal mitochondrial movement. Neurobiology of Disease 127, 410-418.
448. Wang, L., and Brown, A. (2010) A hereditary spastic paraplegia mutation in kinesin-1A/KIF5A disrupts neurofilament transport. Molecular Neurodegeneration 5, 52.
449. Cuchanski, M., and Baldwin, K. (2018) Novel Mutation in KIF5A Causing Hereditary Spastic Paraplegia with Axonal Sensorimotor Neuropathy (P2.444). Neurology 90, P2.444.
450. Qiu, Y., Zhong, S., Cong, L., Xin, L., Gao, X., Zhang, J., and Hong, D. (2018) A novel KIF5A gene variant causes spastic paraplegia and cerebellar ataxia. Annals of Clinical and Translational Neurology 5, 1415-1420.
451. Simone, M., Trabacca, A., Panzeri, E., Losito, L., Citterio, A., and Bassi, M. T. (2018) KIF5A and ALS2 Variants in a Family With Hereditary Spastic Paraplegia and Amyotrophic Lateral Sclerosis. Frontiers in Neurology 9.
452. Filosto, M., Piccinelli, S. C., Palmieri, I., Necchini, N., Valente, M., Zanella, I., Biasiotto, G., Lorenzo, D. D., Cereda, C., and Padovani, A. (2019) A Novel Mutation in the Stalk Domain of KIF5A Causes a Slowly Progressive Atypical Motor Syndrome. Journal of clinical medicine 8, 17.
453. Faruq, M., Kumar, D., Wadhwa, S., Shamim, U., Mathur, A., Parveen, S., Garg, A., and Srivastava, A. K. (2019) Intrafamilial variable spastic paraplegia/ataxia/ALS phenotype linked to a novel KIF5A mutation. Clin Genet 96, 271-273.
454. Zhang, K., Liu, Q., Shen, D., Tai, H., Liu, S., Wang, Z., Shi, J., Fu, H., Wu, S., Ding, Q., et al. (2019) Mutation analysis of KIF5A in Chinese amyotrophic lateral sclerosis patients. Neurobiology of Aging 73, 229.e221-229.e224.
455. Naruse, H., Ishiura, H., Mitsui, J., Takahashi, Y., Matsukawa, T., Sakuishi, K., Nakamagoe, K., Miyake, Z., Tamaoka, A., Goto, J., et al. (2021) Splice-site mutations in KIF5A in the Japanese case series of amyotrophic lateral sclerosis. neurogenetics 22, 11-17.
456. Nakamura, R., Tohnai, G., Atsuta, N., Nakatochi, M., Hayashi, N., Watanabe, H., Yokoi, D., Watanabe, H., Katsuno, M., Izumi, Y., et al. (2021) Genetic and functional analysis of KIF5A variants in Japanese patients with sporadic amyotrophic lateral sclerosis. Neurobiology of Aging 97, 147.e111-147.e117.
457. Baron, D. M., Fenton, A. R., Saez-Atienzar, S., Giampetruzzi, A., Sreeram, A., Shankaracharya, Keagle, P. J., Doocy, V. R., Smith, N. J., Danielson, E. W., et al. (2022) ALS-associated KIF5A mutations abolish autoinhibition resulting in a toxic gain of function. Cell Reports 39.
458. Nakano, J., Chiba, K., and Niwa, S. (2022) An ALS-associated KIF5A mutant forms oligomers and aggregates and induces neuronal toxicity. Genes to Cells 27, 421-435.
459. Nicolas, A., Kenna, K. P., Renton, A. E., Ticozzi, N., Faghri, F., Chia, R., Dominov, J. A., Kenna, B. J., Nalls, M. A., Keagle, P., et al. (2018) Genome-wide Analyses Identify KIF5A as a Novel ALS Gene. Neuron 97, 1267-1288.
460. ERBB4 erb-b2 receptor tyrosine kinase 4 [Homo sapiens (human)]. https://www.ncbi.nlm.nih.gov/gene/2066 (accessed Apr 6, 2024).
461. Wieduwilt, M. J., and Moasser, M. M. (2008) The epidermal growth factor receptor family: Biology driving targeted therapeutics. Cellular and Molecular Life Sciences 65, 1566-1584.
462. Bouyain, S., Longo, P. A., Li, S., Ferguson, K. M., and Leahy, D. J. (2005) The extracellular region of ErbB4 adopts a tethered conformation in the absence of ligand. Proceedings of the National Academy of Sciences 102, 15024-15029.
463. El-Gamal, M. I., Mewafi, N. H., Abdelmotteleb, N. E., Emara, M. A., Tarazi, H., Sbenati, R. M., Madkour, M. M., Zaraei, S.-O., Shahin, A. I., and Anbar, H. S. (2021) A Review of HER4 (ErbB4) Kinase, Its Impact on Cancer, and Its Inhibitors. Molecules 26, 7376.
464. Lucas, L. M., Dwivedi, V., Senfeld, J. I., Cullum, R. L., Mill, C. P., Piazza, J. T., Bryant, I. N., Cook, L. J., Miller, S. T., IV, J. H. L., et al. (2022) The Yin and Yang of ERBB4: Tumor Suppressor and Oncoprotein. Pharmacological Reviews 74, 18-47.

465. Borg, R., Farrugia Wismayer, M., Bonavia, K., Farrugia Wismayer, A., Vella, M., van Vugt, J. J. F. A., Kenna, B. J., Kenna, K. P., Vassallo, N., Veldink, J. H., and Cauchi, R. J. (2021) Genetic analysis of ALS cases in the isolated island population of Malta. European Journal of Human Genetics 29, 604-614.
466. Takahashi, Y., Fukuda, Y., Yoshimura, J., Toyoda, A., Kurppa, K., Moritoyo, H., Belzil, Veronique V., Dion, Patrick A., Higasa, K., Doi, K., et al. (2013) ERBB4 Mutations that Disrupt the Neuregulin-ErbB4 Pathway Cause Amyotrophic Lateral Sclerosis Type 19. The American Journal of Human Genetics 93, 900-905.
467. Wang, F., Liu, X., He, J., Zhang, N., Chen, L., Tang, L., and Fan, D. (2022) Analysis of ERBB4 Variants in Amyotrophic Lateral Sclerosis Within a Chinese Cohort. Frontiers in Neurology 13.
468. Zhang, N., Chen, K.-L., Huang, Y.-Y., Chen, S.-F., Dong, Q., Tan, L., and Yu, J.-T. (2023) A new ERBB4 variant in amyotrophic lateral sclerosis type 19: Case report and review of the literature. Clinical Neurology and Neurosurgery 227, 107636.
469. Falls, D. L., - Neuregulins: Functions, forms, and signaling strategies. In *The EGF Receptor Family*, Carpenter, G., Ed. Academic Press: Burlington, 2003; pp 15-31.
470. Lemmon, M. A. (2009) Ligand-induced ErbB receptor dimerization. Experimental Cell Research 315, 638-648.
471. CCNF cyclin F [Homo sapiens (human)]. https://www.ncbi.nlm.nih.gov/gene/899 (accessed Apr 6, 2024).
472. Bai, C., Richman, R., and Elledge, S. J. (1994) Human cyclin F. The EMBO Journal 13, 6087-6098.
473. Galper, J., Rayner, S. L., Hogan, A. L., Fifita, J. A., Lee, A., Chung, R. S., Blair, I. P., and Yang, S. (2017) Cyclin F: A component of an E3 ubiquitin ligase complex with roles in neurodegeneration and cancer. The International Journal of Biochemistry & Cell Biology 89, 216-220.
474. Fu, J., Qiu, H., Cai, M., Pan, Y., Cao, Y., Liu, L., Yun, J., and Zhang, C. Z. (2013) Low cyclin F expression in hepatocellular carcinoma associates with poor differentiation and unfavorable prognosis. Cancer Science 104, 508-515.
475. Krajewski, A., Gagat, M., Żuryń, A., Hałas-Wiśniewska, M., Grzanka, D., and Grzanka, A. (2020) Cyclin F is involved in response to cisplatin treatment in melanoma cell lines. Oncol Rep 43, 765-772.
476. Zelong, Y., Han, Y., Ting, G., Yifei, W., Kun, H., Haoran, H., and Yong, C. (2021) Increased expression of Cyclin F in liver cancer predicts poor prognosis: A study based on TCGA database. Medicine 100.
477. Williams, K. L., Topp, S., Yang, S., Smith, B., Fifita, J. A., Warraich, S. T., Zhang, K. Y., Farrawell, N., Vance, C., Hu, X., et al. (2016) CCNF mutations in amyotrophic lateral sclerosis and frontotemporal dementia. Nature Communications 7, 11253.
478. Lee, A., Rayner, S. L., Gwee, S. S. L., De Luca, A., Shahheydari, H., Sundaramoorthy, V., Ragagnin, A., Morsch, M., Radford, R., Galper, J., et al. (2018) Pathogenic mutation in the ALS/FTD gene, CCNF, causes elevated Lys48-linked ubiquitylation and defective autophagy. Cellular and Molecular Life Sciences 75, 335-354.
479. Tian, D., Li, J., Tang, L., Zhang, N., and Fan, D. (2018) Screening for CCNF Mutations in a Chinese Amyotrophic Lateral Sclerosis Cohort. Frontiers in Aging Neuroscience 10.
480. Tsai, P.-C., Liao, Y.-C., Chen, P.-L., Guo, Y.-C., Chen, Y.-H., Jih, K.-Y., Lin, K.-P., Soong, B.-W., Tsai, C.-P., and Lee, Y.-C. (2018) Investigating CCNF mutations in a Taiwanese cohort with amyotrophic lateral sclerosis. Neurobiology of Aging 62, 243.e241-243.e246.
481. Yu, Y., Nakagawa, T., Morohoshi, A., Nakagawa, M., Ishida, N., Suzuki, N., Aoki, M., and Nakayama, K. (2019) Pathogenic mutations in the ALS gene CCNF cause cytoplasmic mislocalization of Cyclin F and elevated VCP ATPase activity. Human Molecular Genetics 28, 3486-3497.
482. ANXA11 annexin A11 [Homo sapiens (human)]. https://www.ncbi.nlm.nih.gov/gene/311 (accessed Apr 6, 2024).
483. Mirsaeidi, M., Gidfar, S., Vu, A., and Schraufnagel, D. (2016) Annexins family: insights into their functions and potential role in pathogenesis of sarcoidosis. Journal of Translational Medicine 14, 89.
484. Shibata, H., Kanadome, T., Sugiura, H., Yokoyama, T., Yamamuro, M., Moss, S. E., and Maki, M. (2015) A New Role for Annexin A11 in the Early Secretory Pathway via Stabilizing Sec31A Protein at the Endoplasmic Reticulum Exit Sites (ERES) *. Journal of Biological Chemistry 290, 4981-4993.
485. Dudas, E. F., Tully, M. D., Foldes, T., Kelly, G., Tartaglia, G. G., and Pastore, A. (2024) The structural properties of full-length annexin A11. Frontiers in Molecular Biosciences 11.
486. Sarcoidosis. https://www.mayoclinic.org/diseases-conditions/sarcoidosis/symptoms-causes/syc-20350358 (accessed Apr 6, 2024).

487. Hubers, L. M., Vos, H., Schuurman, A. R., Erken, R., Elferink, R. P. O., Burgering, B., Graaf, S. F. J. v. d., and Beuers, U. (2018) Annexin A11 is targeted by IgG4 and IgG1 autoantibodies in IgG4-related disease. Gut 67, 728-735.
488. Herta, T., Kersten, R., Chang, J.-C., Hubers, L., Go, S., Tolenaars, D., Paulusma, C. C., Nathanson, M. H., Elferink, R. O., van de Graaf, S. F. J., and Beuers, U. (2022) Role of the IgG4-related cholangitis autoantigen annexin A11 in cholangiocyte protection. Journal of Hepatology 76, 319-331.
489. Smith, B. N., Topp, S. D., Fallini, C., Shibata, H., Chen, H.-J., Troakes, C., King, A., Ticozzi, N., Kenna, K. P., Soragia-Gkazi, A., et al. (2017) Mutations in the vesicular trafficking protein annexin A11 are associated with amyotrophic lateral sclerosis. Science Translational Medicine 9, eaad9157.
490. Zhang, K., Liu, Q., Liu, K., Shen, D., Tai, H., Shu, S., Ding, Q., Fu, H., Liu, S., Wang, Z., et al. (2018) <i>ANXA11</i> mutations prevail in Chinese ALS patients with and without cognitive dementia. Neurology Genetics 4, e237.
491. Sainouchi, M., Hatano, Y., Tada, M., Ishihara, T., Ando, S., Kato, T., Tokunaga, J., Ito, G., Miyahara, H., Toyoshima, Y., et al. (2021) A novel splicing variant of ANXA11 in a patient with amyotrophic lateral sclerosis: histologic and biochemical features. Acta Neuropathologica Communications 9, 106.
492. Teyssou, E., Muratet, F., Amador, M.-D.-M., Ferrien, M., Lautrette, G., Machat, S., Boillée, S., Larmonier, T., Saker, S., Leguern, E., et al. (2021) Genetic screening of ANXA11 revealed novel mutations linked to amyotrophic lateral sclerosis. Neurobiology of Aging 99, 102.e111-102.e120.
493. Wang, Y., Duan, X., Zhou, X., Wang, R., Zhang, X., Cao, Z., Wang, X., Zhou, Z., Sun, Y., and Peng, D. (2022) ANXA11 mutations are associated with amyotrophic lateral sclerosis–frontotemporal dementia. Frontiers in Neurology 13.
494. Nahm, M., Lim, S. M., Kim, Y.-E., Park, J., Noh, M.-Y., Lee, S., Roh, J. E., Hwang, S.-M., Park, C.-K., Kim, Y. H., et al. (2020) <i>ANXA11</i> mutations in ALS cause dysregulation of calcium homeostasis and stress granule dynamics. Science Translational Medicine 12, eaax3993.
495. Wang, H., Guan, L., and Deng, M. (2023) Recent progress of the genetics of amyotrophic lateral sclerosis and challenges of gene therapy. Front Neurosci 17, 1170996.
496. Liao, Y.-C., Fernandopulle, M. S., Wang, G., Choi, H., Hao, L., Drerup, C. M., Patel, R., Qamar, S., Nixon-Abell, J., Shen, Y., et al. (2019) RNA Granules Hitchhike on Lysosomes for Long-Distance Transport, Using Annexin A11 as a Molecular Tether. Cell 179, 147-164.e120.
497. Lillebostad, P. A. G., Raasakka, A., Hjellbrekke, S. J., Patil, S., Røstbø, T., Hollås, H., Sakya, S. A., Szigetvari, P. D., Vedeler, A., and Kursula, P. (2020) Structure of the ALS Mutation Target Annexin A11 Reveals a Stabilising N-Terminal Segment. Biomolecules 10, 660.
498. Shihora, A., Elias, R. D., Hammond, J. A., Ghirlando, R., and Deshmukh, L. (2023) ALS Variants of Annexin A11's Proline-Rich Domain Impair Its S100A6-Mediated Fibril Dissolution. ACS Chemical Neuroscience 14, 2583-2589.
499. NEK1 NIMA related kinase 1 [Homo sapiens (human)]. https://www.ncbi.nlm.nih.gov/gene/4750 (accessed Apr 6, 2024).
500. Letwin, K., Mizzen, L., Motro, B., Ben-David, Y., Bernstein, A., and Pawson, T. (1992) A mammalian dual specificity protein kinase, Nek1, is related to the NIMA cell cycle regulator and highly expressed in meiotic germ cells. Embo j 11, 3521-3531.
501. Arama, E., Yanai, A., Kilfin, G., Bernstein, A., and Motro, B. (1998) Murine NIMA-related kinases are expressed in patterns suggesting distinct functions in gametogenesis and a role in the nervous system. Oncogene 16, 1813-1823.
502. Peres de Oliveira, A., Kazuo Issayama, L., Betim Pavan, I. C., Riback Silva, F., Diniz Melo-Hanchuk, T., Moreira Simabuco, F., and Kobarg, J. (2020) Checking NEKs: Overcoming a Bottleneck in Human Diseases. Molecules 25, 1778.
503. Chen, Y., Gaczynska, M., Osmulski, P., Polci, R., and Riley, D. J. (2010) Phosphorylation by Nek1 regulates opening and closing of voltage dependent anion channel 1. Biochemical and Biophysical Research Communications 394, 798-803.
504. Monroe, G. R., Kappen, I. F. P. M., Stokman, M. F., Terhal, P. A., van den Boogaard, M.-J. H., Savelberg, S. M. C., van der Veken, L. T., van Es, R. J. J., Lens, S. M., Hengeveld, R. C., et al. (2016) Compound heterozygous NEK1 variants in two siblings with oral-facial-digital syndrome type II (Mohr syndrome). European Journal of Human Genetics 24, 1752-1760.
505. Thiel, C., Kessler, K., Giessl, A., Dimmler, A., Shalev, S. A., von der Haar, S., Zenker, M., Zahnleiter, D., Stöss, H., Beinder, E., et al. (2011) NEK1 Mutations Cause Short-Rib Polydactyly Syndrome Type Majewski. The American Journal of Human Genetics 88, 106-114.

506. Wang, Z., Horemuzova, E., Iida, A., Guo, L., Liu, Y., Matsumoto, N., Nishimura, G., Nordgren, A., Miyake, N., Tham, E., et al. (2017) Axial spondylometaphyseal dysplasia is also caused by NEK1 mutations. Journal of Human Genetics 62, 503-506.
507. Lattante, S., Doronzio, P. N., Conte, A., Marangi, G., Martello, F., Bisogni, G., Meleo, E., Colavito, D., Del Giudice, E., Patanella, A. K., et al. (2021) Novel variants and cellular studies on patients' primary fibroblasts support a role for NEK1 missense variants in ALS pathogenesis. Hum Mol Genet 30, 65-71.
508. Riva, N., Pozzi, L., Russo, T., Pipitone, G. B., Schito, P., Domi, T., Agosta, F., Quattrini, A., Carrera, P., and Filippi, M. (2022) NEK1 Variants in a Cohort of Italian Patients With Amyotrophic Lateral Sclerosis. Frontiers in Neuroscience 16.
509. Yao, L., He, X., Cui, B., Zhao, F., and Zhou, C. (2021) NEK1 mutations and the risk of amyotrophic lateral sclerosis (ALS): a meta-analysis. Neurol Sci 42, 1277-1285.
510. Kenna, K. P., van Doormaal, P. T. C., Dekker, A. M., Ticozzi, N., Kenna, B. J., Diekstra, F. P., van Rheenen, W., van Eijk, K. R., Jones, A. R., Keagle, P., et al. (2016) NEK1 variants confer susceptibility to amyotrophic lateral sclerosis. Nature Genetics 48, 1037-1042.
511. Nguyen, H. P., Van Mossevelde, S., Dillen, L., De Bleecker, J. L., Moisse, M., Van Damme, P., Van Broeckhoven, C., and van der Zee, J. (2018) NEK1 genetic variability in a Belgian cohort of ALS and ALS-FTD patients. Neurobiol Aging 61, 255.e251-255.e257.
512. BDNF brain derived neurotrophic factor [Homo sapiens (human)]. https://www.ncbi.nlm.nih.gov/gene/627 (accessed Apr 4, 2024).
513. Hofer, M., Pagliusi, S. R., Hohn, A., Leibrock, J., and Barde, Y. A. (1990) Regional distribution of brain-derived neurotrophic factor mRNA in the adult mouse brain. The EMBO Journal 9, 2459-2464-2464.
514. Miranda, M., Morici, J. F., Zanoni, M. B., and Bekinschtein, P. (2019) Brain-Derived Neurotrophic Factor: A Key Molecule for Memory in the Healthy and the Pathological Brain. Frontiers in Cellular Neuroscience 13.
515. Cattaneo, A., Cattane, N., Begni, V., Pariante, C. M., and Riva, M. A. (2016) The human BDNF gene: peripheral gene expression and protein levels as biomarkers for psychiatric disorders. Translational Psychiatry 6, e958-e958.
516. Zuccato, C., Liber, D., Ramos, C., Tarditi, A., Rigamonti, D., Tartari, M., Valenza, M., and Cattaneo, E. (2005) Progressive loss of BDNF in a mouse model of Huntington's disease and rescue by BDNF delivery. Pharmacological Research 52, 133-139.
517. Zuccato, C., and Cattaneo, E. (2007) Role of brain-derived neurotrophic factor in Huntington's disease. Progress in Neurobiology 81, 294-330.
518. Giralt, A., Carretón, O., Lao-Peregrin, C., Martín, E. D., and Alberch, J. (2011) Conditional BDNF release under pathological conditions improves Huntington's disease pathology by delaying neuronal dysfunction. Molecular Neurodegeneration 6, 71.
519. Couly, S., Paucard, A., Bonneaud, N., Maurice, T., Benigno, L., Jourdan, C., Cohen-Solal, C., Vignes, M., and Maschat, F. (2018) Improvement of BDNF signalling by P42 peptide in Huntington's disease. Human Molecular Genetics 27, 3012-3028.
520. Borrell-Pagès, M., Canals, J. M., Cordelières, F. P., Parker, J. A., Pineda, J. R., Grange, G., Bryson, E. A., Guillermier, M., Hirsch, E., Hantraye, P., et al. (2006) Cystamine and cysteamine increase brain levels of BDNF in Huntington disease via HSJ1b and transglutaminase. The Journal of Clinical Investigation 116, 1410-1424.
521. Conforti, P., Zuccato, C., Gaudenzi, G., Ieraci, A., Camnasio, S., Buckley, N. J., Mutti, C., Cotelli, F., Contini, A., and Cattaneo, E. (2013) Binding of the repressor complex REST-mSIN3b by small molecules restores neuronal gene transcription in Huntington's disease models. Journal of Neurochemistry 127, 22-35.
522. Gutierrez, A., Corey-Bloom, J., Thomas, E. A., and Desplats, P. (2020) Evaluation of Biochemical and Epigenetic Measures of Peripheral Brain-Derived Neurotrophic Factor (BDNF) as a Biomarker in Huntington's Disease Patients. Frontiers in Molecular Neuroscience 12.
523. Giampà, C., Montagna, E., Dato, C., Melone, M. A. B., Bernardi, G., and Fusco, F. R. (2013) Systemic Delivery of Recombinant Brain Derived Neurotrophic Factor (BDNF) in the R6/2 Mouse Model of Huntington's Disease. PloS one 8, e64037.
524. Ou, Z.-Y. A., Byrne, L. M., Rodrigues, F. B., Tortelli, R., Johnson, E. B., Foiani, M. S., Arridge, M., De Vita, E., Scahill, R. I., Heslegrave, A., et al. (2021) Brain-derived neurotrophic factor in cerebrospinal fluid and plasma is not a biomarker for Huntington's disease. Scientific reports 11, 3481.

525. SIRT1 sirtuin 1 [Homo sapiens (human)]. https://www.ncbi.nlm.nih.gov/gene/23411 (accessed Apr 4, 2024).
526. Rahman, S., and Islam, R. (2011) Mammalian Sirt1: insights on its biological functions. Cell Communication and Signaling 9, 11.
527. Elibol, B., and Kilic, U. (2018) High Levels of SIRT1 Expression as a Protective Mechanism Against Disease-Related Conditions. Frontiers in Endocrinology 9.
528. Clark, S. J., Falchi, M., Olsson, B., Jacobson, P., Cauchi, S., Balkau, B., Marre, M., Lantieri, O., Andersson, J. C., Jernås, M., et al. (2012) Association of Sirtuin 1 (SIRT1) Gene SNPs and Transcript Expression Levels With Severe Obesity. Obesity 20, 178-185.
529. de Kreutzenberg, S. V., Ceolotto, G., Papparella, I., Bortoluzzi, A., Semplicini, A., Man, C. D., Cobelli, C., Fadini, G. P., and Avogaro, A. (2010) Downregulation of the Longevity-Associated Protein Sirtuin 1 in Insulin Resistance and Metabolic Syndrome: Potential Biochemical Mechanisms. Diabetes 59, 1006-1015.
530. Wang, W., Sun, W., Cheng, Y., Xu, Z., and Cai, L. (2019) Role of sirtuin-1 in diabetic nephropathy. Journal of Molecular Medicine 97, 291-309.
531. Kong, S., McBurney, M. W., and Fang, D. (2012) Sirtuin 1 in immune regulation and autoimmunity. Immunology & Cell Biology 90, 6-13.
532. Jiang, M., Wang, J., Fu, J., Du, L., Jeong, H., West, T., Xiang, L., Peng, Q., Hou, Z., Cai, H., et al. (2012) Neuroprotective role of Sirt1 in mammalian models of Huntington's disease through activation of multiple Sirt1 targets. Nature Medicine 18, 153-158.
533. Duan, W. (2013) Targeting Sirtuin-1 in Huntington’s Disease: Rationale and Current Status. CNS Drugs 27, 345-352.
534. Neo, S. H., and Tang, B. L., Chapter Four - Sirtuins as Modifiers of Huntington's Disease (HD) Pathology. In *Prog Mol Biol Transl Sci*, Zheng, W., Ed. Academic Press: 2018; Vol. 154, pp 105-145.
535. Donmez, G., and Outeiro, T. F. (2013) SIRT1 and SIRT2: emerging targets in neurodegeneration. EMBO Molecular Medicine 5, 344-352.
536. Naia, L., and Rego, A. C. (2015) Sirtuins: double players in Huntington's disease. Biochimica et Biophysica Acta (BBA) - Molecular Basis of Disease 1852, 2183-2194.
537. Jiang, M., Zheng, J., Peng, Q., Hou, Z., Zhang, J., Mori, S., Ellis, J. L., Vlasuk, G. P., Fries, H., Suri, V., and Duan, W. (2014) Sirtuin 1 activator SRT2104 protects Huntington's disease mice. Annals of Clinical and Translational Neurology 1, 1047-1052.
538. Pallos, J., Bodai, L., Lukacsovich, T., Purcell, J. M., Steffan, J. S., Thompson, L. M., and Marsh, J. L. (2008) Inhibition of specific HDACs and sirtuins suppresses pathogenesis in a Drosophila model of Huntington’s disease. Human Molecular Genetics 17, 3767-3775.
539. Westerberg, G., Chiesa, J. A., Andersen, C. A., Diamanti, D., Magnoni, L., Pollio, G., Darpo, B., and Zhou, M. (2015) Safety, pharmacokinetics, pharmacogenomics and QT concentration−effect modelling of the SirT1 inhibitor selisistat in healthy volunteers. British Journal of Clinical Pharmacology 79, 477-491.
540. Süssmuth, S. D., Haider, S., Landwehrmeyer, G. B., Farmer, R., Frost, C., Tripepi, G., Andersen, C. A., Di Bacco, M., Lamanna, C., Diodato, E., et al. (2015) An exploratory double-blind, randomized clinical trial with selisistat, a SirT1 inhibitor, in patients with Huntington's disease. British Journal of Clinical Pharmacology 79, 465-476.
541. HAP1 huntingtin associated protein 1 [Homo sapiens (human)]. https://www.ncbi.nlm.nih.gov/gene/9001 (accessed Apr 4, 2024).
542. Page, K. J., Potter, L., Aronni, S., Everitt, B. J., and Dunnett, S. B. (1998) The expression of Huntingtin-associated protein (HAP1) mRNA in developing, adult and ageing rat CNS: implications for Huntington's disease neuropathology. European Journal of Neuroscience 10, 1835-1845.
543. Mackenzie, K. D., Lim, Y., Duffield, M. D., Chataway, T., Zhou, X.-F., and Keating, D. J. (2017) Huntingtin-associated protein-1 (HAP1) regulates endocytosis and interacts with multiple trafficking-related proteins. Cellular Signalling 35, 176-187.
544. Wu, L. L.-y., and Zhou, X.-F. (2009) Huntingtin associated protein 1 and its functions. Cell Adhesion & Migration 3, 71-76.
545. Chen, X., He, E., Su, C., Zeng, Y., and Xu, J. (2023) Huntingtin-associated protein 1-associated intracellular trafficking in neurodegenerative diseases. Frontiers in Aging Neuroscience 15.
546. Wu, L. L.-y., Fan, Y., Li, S., Li, X.-J., and Zhou, X.-F. (2010) Huntingtin-associated Protein-1 Interacts with Pro-brain-derived Neurotrophic Factor and Mediates Its Transport and Release *. Journal of Biological Chemistry 285, 5614-5623.

547. Rong, J., McGuire, J. R., Fang, Z.-H., Sheng, G., Shin, J.-Y., Li, S.-H., and Li, X.-J. (2006) Regulation of Intracellular Trafficking of Huntingtin-Associated Protein-1 Is Critical for TrkA Protein Levels and Neurite Outgrowth. The Journal of Neuroscience 26, 6019-6030.
548. Metzger, S., Rong, J., Nguyen, H.-P., Cape, A., Tomiuk, J., Soehn, A. S., Propping, P., Freudenberg-Hua, Y., Freudenberg, J., Tong, L., et al. (2008) Huntingtin-associated protein-1 is a modifier of the age-at-onset of Huntington's disease. Human Molecular Genetics 17, 1137-1146.
549. Karadima, G., Dimovasili, C., Koutsis, G., Vassilopoulos, D., and Panas, M. (2012) Age at onset in Huntington's disease: Replication study on the association of HAP1. Parkinsonism & Related Disorders 18, 1027-1028.
550. PPARGC1A PPARG coactivator 1 alpha [Homo sapiens (human)]. https://www.ncbi.nlm.nih.gov/gene/10891 (accessed Apr 4, 2024).
551. Liang, H., and Ward, W. F. (2006) PGC-1α: a key regulator of energy metabolism. Advances in Physiology Education 30, 145-151.
552. Stefan, N., Thamer, C., Staiger, H., Machicao, F., Machann, J. r., Schick, F., Venter, C., Niess, A., Laakso, M., Fritsche, A., and Häring, H.-U. (2007) Genetic Variations in PPARD and PPARGC1A Determine Mitochondrial Function and Change in Aerobic Physical Fitness and Insulin Sensitivity during Lifestyle Intervention. The Journal of Clinical Endocrinology & Metabolism 92, 1827-1833.
553. Eynon, N., Meckel, Y., Sagiv, M., Yamin, C., Amir, R., Sagiv, M., Goldhammer, E., Duarte, J. A., and Oliveira, J. (2010) Do PPARGC1A and PPARα polymorphisms influence sprint or endurance phenotypes? Scandinavian Journal of Medicine & Science in Sports 20, e145-e150.
554. Tural, E., Kara, N., Agaoglu, S. A., Elbistan, M., Tasmektepligil, M. Y., and Imamoglu, O. (2014) PPAR-α and PPARGC1A gene variants have strong effects on aerobic performance of Turkish elite endurance athletes. Molecular Biology Reports 41, 5799-5804.
555. Tharabenjasin, P., Pabalan, N., and Jarjanazi, H. (2019) Association of PPARGC1A Gly428Ser (rs8192678) polymorphism with potential for athletic ability and sports performance: A meta-analysis. PloS one 14, e0200967.
556. Hall, E. C. R., Lockey, S. J., Heffernan, S. M., Herbert, A. J., Stebbings, G. K., Day, S. H., Collins, M., Pitsiladis, Y. P., Erskine, R. M., and Williams, A. G. (2023) The PPARGC1A Gly482Ser polymorphism is associated with elite long-distance running performance. Journal of Sports Sciences 41, 56-62.
557. Soyal, S. M., Zara, G., Ferger, B., Felder, T. K., Kwik, M., Nofziger, C., Dossena, S., Schwienbacher, C., Hicks, A. A., Pramstaller, P. P., et al. (2019) The PPARGC1A locus and CNS-specific PGC-1α isoforms are associated with Parkinson's Disease. Neurobiology of Disease 121, 34-46.
558. Li, L.-z., Zhao, Y.-w., Pan, H.-x., Xiang, Y.-q., Wang, Y.-g., Xu, Q., Yan, X.-x., Tan, J.-q., Li, J.-c., Tang, B.-s., and Guo, J.-f. (2022) Association of rare PPARGC1A variants with Parkinson’s disease risk. Journal of Human Genetics 67, 687-690.
559. Weydt, P., Pineda, V. V., Torrence, A. E., Libby, R. T., Satterfield, T. F., Lazarowski, Eduardo R., Gilbert, M. L., Morton, G. J., Bammler, T. K., Strand, A. D., et al. (2006) Thermoregulatory and metabolic defects in Huntington's disease transgenic mice implicate PGC-1α in Huntington's disease neurodegeneration. Cell Metabolism 4, 349-362.
560. La Spada, A. R. (2012) PPARGC1A/PGC-1α, TFEB and enhanced proteostasis in Huntington disease. Autophagy 8, 1845-1847.
561. Cui, L., Jeong, H., Borovecki, F., Parkhurst, C. N., Tanese, N., and Krainc, D. (2006) Transcriptional Repression of PGC-1α by Mutant Huntingtin Leads to Mitochondrial Dysfunction and Neurodegeneration. Cell 127, 59-69.
562. Weydt, P., Soyal, S. M., Gellera, C., DiDonato, S., Weidinger, C., Oberkofler, H., Landwehrmeyer, G. B., and Patsch, W. (2009) The gene coding for PGC-1α modifies age at onset in Huntington's Disease. Molecular Neurodegeneration 4, 3.
563. Soyal, S. M., Felder, T. K., Auer, S., Hahne, P., Oberkofler, H., Witting, A., Paulmichl, M., Landwehrmeyer, G. B., Weydt, P., Patsch, W., and Network, F. t. E. H. D. (2012) A greatly extended PPARGC1A genomic locus encodes several new brain-specific isoforms and influences Huntington disease age of onset†. Human Molecular Genetics 21, 3461-3473.
564. CNTF ciliary neurotrophic factor [Homo sapiens (human)]. https://www.ncbi.nlm.nih.gov/gene/1270 (accessed Apr 4, 2024).
565. Ramaswamy, S., and Kordower, J. H., 11 - GENE AND CELLULAR TRANSPLANTATION THERAPIES FOR HUNTINGTON'S DISEASE. In *CNS Regeneration (Second Edition)*, Kordower, J. H.; Tuszynski, M. H., Eds. Academic Press: San Diego, 2008; pp 267-294.

566. CNTF Signaling Pathways. https://www.rndsystems.com/pathways/cntf-signaling-pathways (accessed Apr 4, 2024).
567. Barres, B. A., Burne, J. F., Holtmann, B., Thoenen, H., Sendtner, M., and Raff, M. C. (1996) Ciliary Neurotrophic Factor Enhances the Rate of Oligodendrocyte Generation. Molecular and Cellular Neuroscience 8, 146-156.
568. Linker, R. A., Mäurer, M., Gaupp, S., Martini, R., Holtmann, B., Giess, R., Rieckmann, P., Lassmann, H., Toyka, K. V., Sendtner, M., and Gold, R. (2002) CNTF is a major protective factor in demyelinating CNS disease: A neurotrophic cytokine as modulator in neuroinflammation. Nature Medicine 8, 620-624.
569. Giess, R., Mäurer, M., Linker, R., Gold, R., Warmuth-Metz, M., Toyka, K. V., Sendtner, M., and Rieckmann, P. (2002) Association of a Null Mutation in the CNTF Gene With Early Onset of Multiple Sclerosis. Archives of Neurology 59, 407-409.
570. Kuhlmann, T., Remington, L., Cognet, I., Bourbonniere, L., Zehntner, S., Guilhot, F., Herman, A., Guay-Giroux, A., Antel, J. P., Owens, T., and Gauchat, J.-F. (2006) Continued Administration of Ciliary Neurotrophic Factor Protects Mice from Inflammatory Pathology in Experimental Autoimmune Encephalomyelitis. The American Journal of Pathology 169, 584-598.
571. Masu, Y., Wolf, E., Holtmann, B., Sendtner, M., Brem, G., and Thoenen, H. (1993) Disruption of the CNTF gene results in motor neuron degeneration. Nature 365, 27-32.
572. Alberch, J., Pérez-Navarro, E., and Canals, J. M., Neurotrophic factors in Huntington's disease. In *Progress in Brain Research*, Elsevier: 2004; Vol. 146, pp 197-229.
573. Mittoux, V., Ouary, S., Monville, C., Lisovoski, F., Poyot, T., Condé, F., Escartin, C., Robichon, R., Brouillet, E., Peschanski, M., and Hantraye, P. (2002) Corticostriatopallidal Neuroprotection by Adenovirus-Mediated Ciliary Neurotrophic Factor Gene Transfer in a Rat Model of Progressive Striatal Degeneration. The Journal of Neuroscience 22, 4478-4486.
574. Bloch, J., Bachoud-Lévi, A. C., Déglon, N., Lefaucheur, J. P., Winkel, L., Palfi, S., Nguyen, J. P., Bourdet, C., Remy, P., Brugières, P., et al. (2004) Neuroprotective Gene Therapy for Huntington's Disease, Using Polymer-Encapsulated Cells Engineered to Secrete Human Ciliary Neurotrophic Factor: Results of a Phase I Study. Human Gene Therapy 15, 968-975.
575. Escartin, C., Hantraye, P., and Déglon, N., 22 - Transplants of CNTF-producing Cells for the Treatment of Huntington's Disease. In *Cellular Transplantation*, Halberstadt, C.; Emerich, D., Eds. Academic Press: Burlington, 2007; pp 385-398.
576. Denovan-Wright, E. M., Attis, M., Rodriguez-Lebron, E., and Mandel, R. J. (2008) Sustained striatal ciliary neurotrophic factor expression negatively affects behavior and gene expression in normal and R6/1 mice. Journal of Neuroscience Research 86, 1748-1757.
577. IGF1 insulin like growth factor 1 [Homo sapiens (human)]. https://www.ncbi.nlm.nih.gov/gene/3479 (accessed Apr 4, 2024).
578. Pang, A. L.-Y., and Chan, W.-Y., Chapter 22 - Molecular Basis of Diseases of the Endocrine System. In *Essential Concepts in Molecular Pathology*, Coleman, W. B.; Tsongalis, G. J., Eds. Academic Press: San Diego, 2010; pp 289-307.
579. Li, J., Choi, E., Yu, H., and Bai, X.-c. (2019) Structural basis of the activation of type 1 insulin-like growth factor receptor. Nature Communications 10, 4567.
580. Delafontaine, P., Song, Y.-H., and Li, Y. (2004) Expression, Regulation, and Function of IGF-1, IGF-1R, and IGF-1 Binding Proteins in Blood Vessels. Arteriosclerosis, Thrombosis, and Vascular Biology 24, 435-444.
581. Gigantism and Acromegaly. https://www.ncbi.nlm.nih.gov/books/NBK538261/ (accessed Apr 4, 2024).
582. Junnila, R. K., List, E. O., Berryman, D. E., Murrey, J. W., and Kopchick, J. J. (2013) The GH/IGF-1 axis in ageing and longevity. Nature Reviews Endocrinology 9, 366-376.
583. Wrigley, S., Arafa, D., and Tropea, D. (2017) Insulin-Like Growth Factor 1: At the Crossroads of Brain Development and Aging. Frontiers in Cellular Neuroscience 11.
584. Gil-Polo, C., Martinez-Horta, S.-I., Sampedro Santalo, F., Martín-Palencia, M., Gundín-Menéndez, S., Alvarez-Baños, P., Maza-Pereg, L., Calvo, S., Collazo, C., Alonso-García, E., et al. (2023) Association Between Insulin-Like Growth Factor-1 and Social Cognition in Huntington's Disease. Movement Disorders Clinical Practice 10, 279-284.

585. Salem, L., Saleh, N., Désaméricq, G., Youssov, K., Dolbeau, G., Cleret, L., Bourhis, M.-L., Azulay, J.-P., Krystkowiak, P., Verny, C., et al. (2016) Insulin-Like Growth Factor-1 but Not Insulin Predicts Cognitive Decline in Huntington's Disease. PloS one 11, e0162890.
586. Moll, L., Ben-Gedalya, T., Reuveni, H., and Cohen, E. (2016) The inhibition of IGF-1 signaling promotes proteostasis by enhancing protein aggregation and deposition. The FASEB Journal 30, 1656-1669.
587. Lopes, C., Ribeiro, M., Duarte, A. I., Humbert, S., Saudou, F., Pereira de Almeida, L., Hayden, M., and Rego, A. C. (2014) IGF-1 Intranasal Administration Rescues Huntington's Disease Phenotypes in YAC128 Mice. Molecular Neurobiology 49, 1126-1142.
588. AGRN agrin [Homo sapiens (human)]. https://www.ncbi.nlm.nih.gov/gene/375790 (accessed Apr 4, 2024).
589. Yan, M., Xing, G.-L., Xiong, W.-C., and Mei, L. (2018) Agrin and LRP4 antibodies as new biomarkers of myasthenia gravis. Annals of the New York Academy of Sciences 1413, 126-135.
590. Donahue, J. E., Berzin, T. M., Rafii, M. S., Glass, D. J., Yancopoulos, G. D., Fallon, J. R., and Stopa, E. G. (1999) Agrin in Alzheimer's disease: Altered solubility and abnormal distribution within microvasculature and brain parenchyma. Proceedings of the National Academy of Sciences 96, 6468-6472.
591. Cotman, S. L., Halfter, W., and Cole, G. J. (2000) Agrin Binds to β-Amyloid (Aβ), Accelerates Aβ Fibril Formation, and Is Localized to Aβ Deposits in Alzheimer's Disease Brain. Molecular and Cellular Neuroscience 15, 183-198.
592. Liu, I.-H., Uversky, V. N., Munishkina, L. A., Fink, A. L., Halfter, W., and Cole, G. J. (2005) Agrin binds α-synuclein and modulates α-synuclein fibrillation. Glycobiology 15, 1320-1331.
593. Skeffington, K. L., Jones, F. P., Suleiman, M. S., Caputo, M., Brancaccio, A., and Bigotti, M. G. (2022) Determination of Agrin and Related Proteins Levels as a Function of Age in Human Hearts. Frontiers in Cardiovascular Medicine 9.
594. Jury, E. C., and Kabouridis, P. S. (2010) New role for Agrin in T cells and its potential importance in immune system regulation. Arthritis Research & Therapy 12, 205.
595. Gasperi, C., Melms, A., Schoser, B., Zhang, Y., Meltoranta, J., Risson, V., Schaeffer, L., Schalke, B., and Kröger, S. (2014) Anti-agrin autoantibodies in myasthenia gravis. Neurology 82, 1976-1983.
596. Zhang, B., Shen, C., Bealmear, B., Ragheb, S., Xiong, W.-C., Lewis, R. A., Lisak, R. P., and Mei, L. (2014) Autoantibodies to Agrin in Myasthenia Gravis Patients. PloS one 9, e91816.
597. Rivner, M. H., Quarles, B. M., Pan, J.-X., Yu, Z., Howard Jr, J. F., Corse, A., Dimachkie, M. M., Jackson, C., Vu, T., Small, G., et al. (2020) Clinical features of LRP4/agrin-antibody–positive myasthenia gravis: A multicenter study. Muscle & Nerve 62, 333-343.
598. Yan, M., Liu, Z., Fei, E., Chen, W., Lai, X., Luo, B., Chen, P., Jing, H., Pan, J.-x., Rivner, M. H., et al. (2018) Induction of Anti-agrin Antibodies Causes Myasthenia Gravis in Mice. Neuroscience 373, 113-121.
599. LRP4 LDL receptor related protein 4 [Homo sapiens (human)]. https://www.ncbi.nlm.nih.gov/gene/4038 (accessed Apr 4, 2024).
600. Chen, B.-H., Lin, Z.-Y., Zeng, X.-X., Jiang, Y.-H., and Geng, F. (2024) LRP4-related signalling pathways and their regulatory role in neurological diseases. Brain Research 1825, 148705.
601. Higuchi, O., Hamuro, J., Motomura, M., and Yamanashi, Y. (2011) Autoantibodies to low-density lipoprotein receptor–related protein 4 in myasthenia gravis. Annals of Neurology 69, 418-422.
602. Pevzner, A., Schoser, B., Peters, K., Cosma, N.-C., Karakatsani, A., Schalke, B., Melms, A., and Kröger, S. (2012) Anti-LRP4 autoantibodies in AChR- and MuSK-antibody-negative myasthenia gravis. Journal of Neurology 259, 427-435.
603. Zhang, B., Tzartos, J. S., Belimezi, M., Ragheb, S., Bealmear, B., Lewis, R. A., Xiong, W.-C., Lisak, R. P., Tzartos, S. J., and Mei, L. (2012) Autoantibodies to Lipoprotein-Related Protein 4 in Patients With Double-Seronegative Myasthenia Gravis. Archives of Neurology 69, 445-451.
604. MUSK muscle associated receptor tyrosine kinase [Homo sapiens (human)]. https://www.ncbi.nlm.nih.gov/gene/4593 (accessed Apr 4, 2024).
605. Zong, Y., Zhang, B., Gu, S., Lee, K., Zhou, J., Yao, G., Figueiredo, D., Perry, K., Mei, L., and Jin, R. (2012) Structural basis of agrin–LRP4–MuSK signaling. Genes & Development 26, 247-258.
606. Zong, Y., and Jin, R. (2013) Structural mechanisms of the agrin–LRP4–MuSK signaling pathway in neuromuscular junction differentiation. Cellular and Molecular Life Sciences 70, 3077-3088.
607. Borges, L. S., and Richman, D. P. (2020) Muscle-Specific Kinase Myasthenia Gravis. Frontiers in Immunology 11.

608. Maselli, R. A., Arredondo, J., Cagney, Ó., Ng, J. J., Anderson, J. A., Williams, C., Gerke, B. J., Soliven, B., and Wollmann, R. L. (2010) Mutations in MUSK causing congenital myasthenic syndrome impair MuSK–Dok-7 interaction. Human Molecular Genetics 19, 2370-2379.
609. McLean, A., and Wilson, I. (2023) Congenital myasthenic syndrome from a MUSK gene mutation. Practical Neurology, pn-2023-003945.
610. Tan-Sindhunata, M. B., Mathijssen, I. B., Smit, M., Baas, F., de Vries, J. I., van der Voorn, J. P., Kluijt, I., Hagen, M. A., Blom, E. W., Sistermans, E., et al. (2015) Identification of a Dutch founder mutation in MUSK causing fetal akinesia deformation sequence. European Journal of Human Genetics 23, 1151-1157.
611. Wilbe, M., Ekvall, S., Eurenius, K., Ericson, K., Casar-Borota, O., Klar, J., Dahl, N., Ameur, A., Annerén, G., and Bondeson, M.-L. (2015) MuSK: a new target for lethal fetal akinesia deformation sequence (FADS). Journal of Medical Genetics 52, 195-202.
612. Fetal akinesia deformation sequence. https://www.orpha.net/en/disease/detail/994#:~:text=The%20fetal%20akinesia%2Fhypokinesia%20sequence,sequence%20is%20decreased%20foetal%20activity. (accessed Apr 4, 2024).
613. FETAL AKINESIA DEFORMATION SEQUENCE 1; FADS1. https://www.omim.org/entry/208150 (accessed Apr 4, 2024).
614. Rodolico, C., Bonanno, C., Toscano, A., and Vita, G. (2020) MuSK-Associated Myasthenia Gravis: Clinical Features and Management. Frontiers in Neurology 11.
615. König, N., Stetefeld, H. R., Dohmen, C., Mergenthaler, P., Kohler, S., Schönenberger, S., Bösel, J., Lee, D.-H., Gerner, S. T., Huttner, H. B., et al. (2021) MuSK-antibodies are associated with worse outcome in myasthenic crisis requiring mechanical ventilation. Journal of Neurology 268, 4824-4833.
616. Lavrnic, D., Losen, M., Vujic, A., Baets, M. D., Hajdukovic, L. J., Stojanovic, V., Trikic, R., Djukic, P., and Apostolski, S. (2005) The features of myasthenia gravis with autoantibodies to MuSK. Journal of Neurology, Neurosurgery & Psychiatry 76, 1099-1102.
617. Huang, Q., Li, F., and Zhao, S. (2022) Spotlight on MuSK positive myasthenia gravis: clinical characteristics, treatment and outcomes. BMC Neurology 22, 73.
618. Zhou, Y., Chen, J., Li, Z., Tan, S., Yan, C., Luo, S., Zhou, L., Song, J., Huan, X., Wang, Y., et al. (2022) Clinical Features of Myasthenia Gravis With Antibodies to MuSK Based on Age at Onset: A Multicenter Retrospective Study in China. Frontiers in Neurology 13.
619. Huijbers, M. G., Zhang, W., Klooster, R., Niks, E. H., Friese, M. B., Straasheijm, K. R., Thijssen, P. E., Vrolijk, H., Plomp, J. J., Vogels, P., et al. (2013) MuSK IgG4 autoantibodies cause myasthenia gravis by inhibiting binding between MuSK and Lrp4. Proceedings of the National Academy of Sciences 110, 20783-20788.
620. TTN titin [Homo sapiens (human)]. https://www.ncbi.nlm.nih.gov/gene/7273 (accessed Apr 4, 2024).
621. Molecule of the Month: Titin. https://pdb101.rcsb.org/motm/185#:~:text=Titin%20is%20the%20largest%20protein,organizes%20the%20thin%20actin%20filaments. (accessed Apr 4, 2024).
622. Herzog, W. (2018) The multiple roles of titin in muscle contraction and force production. Biophysical Reviews 10, 1187-1199.
623. Pfeffer, G., Joseph, J. T., Innes, A. M., Frizzell, J. B., Wilson, I. J., Brownell, A. K. W., and Chinnery, P. F. (2014) Titinopathy in a Canadian Family Sharing the British Founder Haplotype. Canadian Journal of Neurological Sciences / Journal Canadien des Sciences Neurologiques 41, 90-94.
624. Oates, E. C., Jones, K. J., Donkervoort, S., Charlton, A., Brammah, S., Smith III, J. E., Ware, J. S., Yau, K. S., Swanson, L. C., Whiffin, N., et al. (2018) Congenital Titinopathy: Comprehensive characterization and pathogenic insights. Annals of Neurology 83, 1105-1124.
625. Huang, K., Duan, H.-Q., Li, Q.-X., Luo, Y.-B., Bi, F.-F., and Yang, H. (2021) Clinicopathological features of titinopathy from a Chinese neuromuscular center. Neuropathology 41, 349-356.
626. Yamamoto, A. M., Gajdos, P., Eymard, B., Tranchant, C., Warter, J.-M., Gomez, L., Bourquin, C., Bach, J.-F., and Garchon, H.-J. (2001) Anti-Titin Antibodies in Myasthenia Gravis: Tight Association With Thymoma and Heterogeneity of Nonthymoma Patients. Archives of Neurology 58, 885-890.
627. Chen, X. J., Qiao, J., Xiao, B. G., and Lu, C. Z. (2004) The significance of titin antibodiesin myasthenia gravis. Journal of Neurology 251, 1006-1011.

628. Kim, K. H., Kim, S. W., Cho, J., Chung, H. Y., and Shin, H. Y. (2022) Anti-titin antibody is associated with more frequent hospitalization to manage thymoma-associated myasthenia gravis. Frontiers in Neurology 13.
